# Mixed evidence for adaptation to environmental pollution

**DOI:** 10.1111/eva.12782

**Published:** 2019-04-09

**Authors:** Alessandra Loria, Melania E. Cristescu, Andrew Gonzalez

**Affiliations:** ^1^ Department of Biology McGill University Montreal Québec Canada

**Keywords:** genetic variation, meta‐analysis, phenotypic response, pollution, population persistence, resistance, selection

## Abstract

Adaptation to pollution has been studied since the first observations of heavy metal tolerance in plants decades ago. To document micro‐evolutionary responses to pollution, researchers have used phenotypic, molecular genetics, and demographic approaches. We reviewed 258 articles and evaluated the evidence for adaptive responses following exposure to a wide range of pollutants, across multiple taxonomic groups. We also conducted a meta‐analysis to calculate the magnitude of phenotypic change in invertebrates in response to metal pollution. The majority of studies that reported differences in responses to pollution were focused on phenotypic responses at the individual level. Most of the studies that used demographic assays in their investigations found that negative effects induced by pollution often worsened over multiple generations. Our meta‐analysis did not reveal a significant relationship between metal pollution intensity and changes in the traits studied, and this was probably due to differences in coping responses among different species, the broad array of abiotic and biotic factors, and the weak statistical power of the analysis. We found it difficult to make broad statements about how likely or how common adaptation is in the presence of environmental contamination. Ecological and evolutionary responses to contamination are complex, and difficult to interpret in the context of taxonomic, and methodological biases, and the inconsistent set of approaches that have been used to study adaptation to pollution in the laboratory and in the field. This review emphasizes the need for: (a) long‐term monitoring programs on exposed populations that link demography to phenotypic, genetic, and selection assays; (b) the use of standardized protocols across studies especially for similar taxa. Approaches that combine field and laboratory studies offer the greatest opportunity to reveal the complex eco‐evolutionary feedback that can occur under selection imposed by pollution.

## 1 INTRODUCTION

Humans have been described as the world's greatest evolutionary force with pollution as one of the most potent forces of ecological and evolutionary change (Palumbi, 2001). However, how often evolution can result in an adaptive response to contaminants remains largely unknown (Brady, Monosson, Matson, & Bickham, 2017). Fossil fuel combustion, the application of synthetic fertilizers and pesticides in agriculture, and the increasing use of complex chemicals are considered the main causes of pollution. For example, the number of complex chemicals is rapidly increasing. In Europe alone, more than 100,000 substances have been recorded in the market (United Nations Environmental Programme [UNEP], 2012). Over the last 40 years, the long‐term effects of pollutants on the sustainability of ecosystem processes have become a significant concern of the scientific community and regulatory agencies (Bickham, Sandhu, Hebert, Chikhi, & Athwal, 2000).

**Figure 1:**
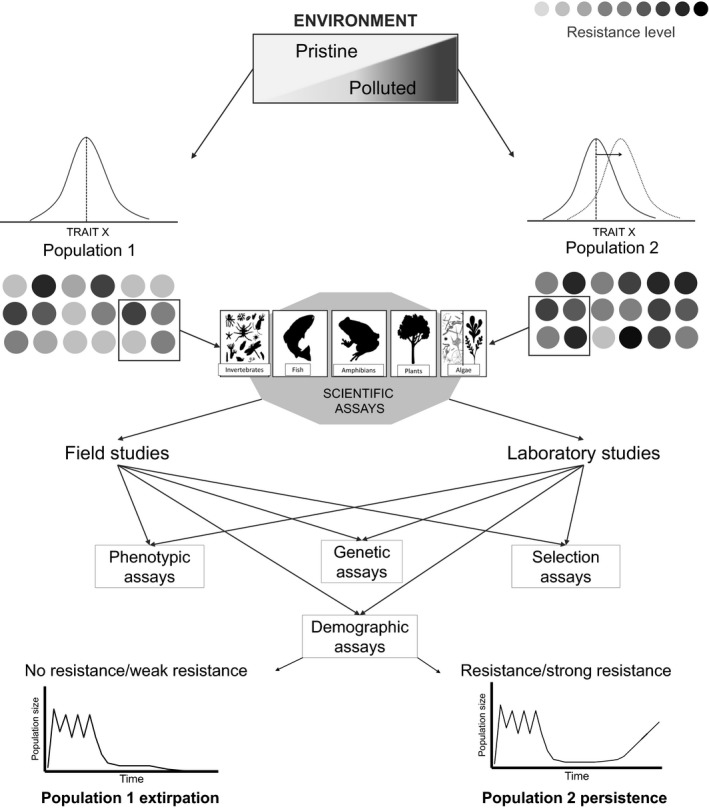
A diagram illustrating two populations that undergo different selection pressures and are used to study their phenotypic, genetic, and selective responses in laboratory and field assays. Pollution acts as a selective force for resistant phenotypes in population 2, which shows higher resistance to pollution than population 1. If the advantageous alleles reach fixation and the population growth rate is positive, then population 2 can recover and persist in the polluted environment by adaptation. However, if the number of selective deaths is too high, or if maladapted phenotypes lower the local absolute fitness below the replacement rate, then population 2 might go extinct. The degree of pollution, phenotypic variation, strength of selection, and population size and the interspecific interactions are all key factors in determining whether a population can persist through genetic adaptation in contaminated locations. Adaptation to pollution has been studied in the laboratory and field. When studied in the field, phenotypic trait variability and population sizes can be jointly monitored over time to reveal covariation that is consistent with increasing fitness. Reciprocal transplant and common garden experiment are possible in the field, which provides greater control over confounding environmental factors. Under laboratory conditions, a large number of repeated tests can be performed (phenotypic, genetic, selection, and population assays) in the short term and long term, either phenotypic and genetic assays with single individuals, or with entire populations, where demographic processes for invertebrates and annual plants are studied over multiple generations

The intensity, extent, and duration of pollution are important factors in determining whether a population can survive in the short term or persist and evolve in the long term. In the presence of reachable alternative habitats, dispersal can enable population persistence. However, when dispersal is limited or suitable habitats are not available, escaping stressful conditions is often not possible. In the short term and in the presence of weak levels of pollution, organisms can adjust their phenotypes (e.g., physiology, behavior) by means of plastic responses without changes in genetic composition (Gienapp, Teplitsky, Alho, Mills, & Merilä, 2008). Moreover, when the level of pollution is persistently elevated and mortality is high, populations can become maladapted because of the presence of phenotypes lacking advantageous traits; standing phenotypes might be so maladapted that the loss of absolute fitness (*W*_abs_) results in population decline (maladaptation in the strict sense; Brandon, 1990; Hendry & Gonzalez, 2008). In many cases, the population will be extirpated; however, in some cases individuals with advantageous traits and genetically inherited resistance to pollution may arise, recovering the absolute fitness (*W*_abs_ > 1) and resulting in population recovery through the process of evolutionary rescue (Gonzalez, Ronce, Ferriere, & Hochberg, 2013; Figure 1). The lack of functionally advantageous variation affecting traits such as survival, reproduction, and other life‐history traits is perhaps one of the most common constraints to evolution in polluted habitats (Blows & Hoffmann, 2005; Bradshaw, 1991; Fisher, 1930). However, the selection of resistant phenotypes alone does not guarantee that a population will persist through adaptation. Small populations may undergo rapid extinction due to demographic and environmental stochasticity before they can recover (Bell & Gonzalez, ; Gomulkiewicz & Holt, 1995; Gonzalez et al., 2013; Lande, ; Lynch & Lande, 1993). Moreover, the effects of induced mutations caused by chronic exposures to mutagens can be exacerbated in small or declining populations, leading to “mutational meltdown,” a process similar to a chain reaction in which the decrease in fitness due to mutations leads to further reduction in population size creating further decrease in fitness (Lynch, Conery, & Burger, 1995).

The assessment of adaptive responses in natural populations should ideally involve field studies focused on phenotypic traits and/or the underlying molecular markers, and population monitoring over time (Figure 1). However, this is often challenging, particularly for species with long generation time. As a result, many studies are restricted to comparing populations living under contrasting environmental conditions (Hansen, Olivieri, Waller, & Nielsen, 2012). This approach, however, gives rise to problems concerning the unknown genetic history of the populations studied and does not take into account the fact that sensitive populations may disappear before investigations are conducted. Artificial selection experiments, and studies of the evolutionary potential in naïve populations, represent another approach to evaluate micro‐evolutionary effect of pollutants (De Coninck, Janssen, & Schamphelaere, 2013). Such studies can provide accurate measurements of heritability, and fitness, including population growth rates, which are required to pinpoint the reasons for population persistence (Figure 1; Klerks, Xie, & Levinton, 2011; Oziolor, Schamphelaere, & Matson, 2016). Regardless of the main approach used, studies aiming to demonstrate adaptive evolutionary change should satisfy certain criteria (Hansen et al., 2012).

To document micro‐evolutionary changes and to demonstrate the genetic basis of adaptation to pollution studies should ideally: (a) identify a trait(s) that can provide a fitness advantage in dealing with the stressor, (b) assess the presence of suitable genetic variation for the particular trait(s); (c) show that selection (as opposed to genetic drift) has taken place; (d) assess the contribution of the advantageous trait(s) to the population fitness by estimating population growth rate (Bell, 2012; Bickham et al., 2000; Gomulkiewicz & Holt, 1995; Hansen et al., 2012; Klerks et al., 2011; Merilä & Hendry, 2014). Although collectively these criteria are quite stringent, many of them can be satisfied by either focusing research efforts on quantitative trait analysis or testing for selection at candidate molecular markers (Hansen et al., 2012).

Several literature reviews have summarized studies focused on the ability of organisms to tolerate pollutants (Klerks et al., 2011; Weis, 2002; Wirgin & Waldman, 2004), on the genetic effects of pollution (Bijlsma & Loeschcke, 2012; DiBattista, 2008; Gillespie & Guttman, 1999; Hoffmann & Daborn, 2007), on genetic resistance to pollution (Roelofs, Morgan, & Stürzenbaum, 2010; Whitehead, Triant, Champlin, & Nacci, 2010), on micro‐evolutionary effects of chemical stressors (De Coninck et al., 2013; Oziolor et al., 2016), and on evolutionary limits of adaptive changes (Bell, 2012; Blows & Hoffmann, 2005; Shaw, 1999; Willi, Buskirk, & Hoffmann, 2006). However, a broad synthesis of strategies and trends in evolutionary toxicology research encompassing multiple levels (e.g., taxonomic, methodological, molecular, demographic) is still lacking.

To get a better understanding of what is currently known about adaptation to pollution, we conducted a literature review that encompasses multiple levels or organization (genetic, individual, and population level), taxonomic groups (algae, plants, invertebrates, and vertebrates), methods (field and laboratory studies), and pollutants (metals, acidification, PAHs, PCBs, etc.). We performed a quantitative meta‐analysis with a subset of the data to evaluate the effect of metal pollution on the magnitude of phenotypic response (e.g., weight, number of offspring, and metal body content) in invertebrates. We also evaluated how shifts in methodological approaches have changed our understanding of micro‐evolutionary responses to pollution. In particular, we assessed molecular evidence for adaptation and the candidate genes potentially involved in the pollution‐induced evolutionary processes (Supporting Information).

## 2 METHODS

In this study, we reviewed articles published since 1992 found by searching on *Google Scholar*. We used the keywords “genetic adaptation,” “adaptation,” “micro‐evolution” in combination with “pollution” or “pollutants” or “contaminants.” These search terms reduced bias to a particular approach or method.

We also searched with “identification of candidate genes AND pollution/pollutants/contaminants” and “genomics OR transcriptomics AND pollution/pollutants/contaminants” to collect papers focused on the identification of candidate genes involved in resistance to pollution for our descriptive compilation (Supporting Information). We also reviewed the articles listed in the bibliographies of the retrieved papers and reviews with titles that pertained to adaptation to pollution. Studies focused only on toxic effects (e.g., deleterious mutations) were excluded. Our search returned a total of 258 papers corresponding to 278 studies (complete references list in Supporting Information). The vast majority of these articles investigated only one species, another twelve articles investigated two species, and one article assessed three species. The number of studies indicated in our figures assumes each species as a different study. The articles were classified based on the type of pollution, the species studied, source populations (e.g., from contaminated and reference sites or laboratory cultures), genetic methods, type of study (field vs. laboratory study), and type of response (Supporting Information Tables S1 and S2). We considered studies on algae, plants, invertebrates, and vertebrates that focused on the effects of metals, acidification (terrestrial and ocean acidification caused by CO_2_ increase), polycyclic aromatic hydrocarbons (PAHs), polychlorinated biphenyls (PCBs), and various other chemical pollutants (identified as “other”; Supporting Information Figure S1) on several phenotypic traits and genetics at both individual and population levels. Some of the articles included investigations on more than one pollutant (Supporting Information Figure S2). We did not consider thermal, visual, and noise pollution. We also excluded studies on simplified agricultural systems where agrochemicals were intentionally applied in the environment.

We identified a set of response variables that are measured in laboratory and field experiments to study the evolution of resistance through molecular markers and/or quantitative traits. Studies were then assessed and classified according to the approach(es) used: (a) analysis of phenotypic responses to pollution leading to resistance (“phenotypic assays”); (b) characterization of the genetic basis/underlying genetic variation of the advantageous phenotypic traits (“genetic assays”); (c) tests for evidence of selection against random genetic drift and gene flow (“selection assays”); (d) assessment of population growth rate (“demographic assays”; Vasemägi & Primmer, 2005; Hansen et al., 2012; Merilä & Hendry, 2014; Table 1, Supporting Information Table S2). Phenotypic assays consisted of laboratory and field phenotypic surveys and were not limited to studies that ruled out plasticity. Genetic assays consisted of laboratory and field common garden experiments as well as molecular assays aiming to explore the genetic basis of advantageous traits, decompose total variation into its components (disentangling genetic and environmental bases for trait variation), and identify and analyze functional DNA polymorphisms. However, heritability estimates, extrapolated within common garden experiments, may not accurately predict the trait's selection response and the ability to evolve resistance (Klerks et al., 2011). Selection assays inferred the adaptive basis of trait change by studying how changing trait values reflected patterns of selection (i.e., animal model analyses, methods that compared differentiations for quantitative traits to those for neutral genetic markers) and changes in allele frequencies (i.e., *F*_ST_‐based outlier tests). In these cases, random genetic drift was ruled out. Since it is common in evolution studies to substitute time for space and use geographic variation in resistance as an alternative for temporal changes (the clean site represents the state of the contaminated site prior to contamination; Byars, Papst, & Hoffmann, 2007; Klerks et al., 2011), these types of investigations were included in the selection category. Some of the standard descriptors of basic experimental designs and analyses included more than one assay category. For example, field and laboratory common garden experiments were represented by both phenotypic and genetic assays since the study of phenotypic trait responses is usually followed by estimates of heritability. Phenotypic selection estimates were represented by both phenotypic and selection assays; genotypic selection estimates by genetic and selection assays. We noted whether a phenotypic response was documented in multiple studies (focused on the same population) and whether (following evidence for selection) further tests were performed to ensure that selection was due to pollution and not to other confounding factors. We grouped studies conducted on the same population(s) and considered them as one composite study while aggregating their methods and outcomes (Supporting Information Table S2). We obtained a subset of 108 articles on invertebrates that focused on metals specifically cadmium, copper, lead, and zinc. We subjected these to a formal meta‐analysis to evaluate the magnitude of the phenotypic response (change in the weight, number of offspring, and metal body content) to different metal concentrations. We focused on the taxonomic groups, pollutants, and response variables that are commonly reported in the literature. Following methods from Collins (1992) and Mondol, Nasrin, and Nahar (2016), we were able to convert length traits into weight measures, which added three more studies (Haimi, Knott, Selonen, & Laurikainen, 2006; Venier et al., 2006; Yap, Cheng, Ong, & Tan, 2013) and 15 additional datapoints. We recorded the metal concentration, the response of treatments and controls at each concentration, the total sample size, and the *SD*. If the study provided only the *SE*, the *SD* was calculated by multiplying the *SE* by the square root of sample size; when only confidence intervals were provided and when the sample size was >60, we applied the formula *SD* = √sample size (upper limit − lower limit)/3.92. When the sample size was <60, we replaced 3.92 with values obtained from tables of the *t* distribution with degree of freedom equal to the group sample size minus 1.

**Table 1:** Synopsis of phenotypic, genetic, and selection assays for inferring phenotypic responses, presence of suitable genetic variation and a response to selection for resistance to pollution. Methods to find a link between the selection detected and the type of pollution studied are also shown. The numbers in parentheses are used in Supporting Information Table S2 to classify the reviewed articles

Phenotypic assays	Genetic assays		Selection assays	
Phenotype	Quantitative traits	Molecular markers	Quantitative traits	Molecular markers
Survival (1)	Quantitative trait locus (QTL) analyses (7)		*Q*_ST_‐*F*_ST_ comparisons (19)	*F*_ST_‐based outlier tests (23)
Growth traits (2)	Admixture mapping (8)		Trait direction of changes in the wild (20)	Detection of selective sweeps (24)
Physiological traits (3)	Association analysis (9)		Tests on neutrality of rates of evo (21)	Genetic association tests (25)
Developmental traits (4)	Additive genetic variance, heritability (10)	QTL mapping of mRNA expression (14)	Pedigreeing, animal model analysis (22)	Genome scan approaches (26)
Morphological traits (5)	Broad‐sense heritability (11)	QTL mapping of protein expression (15)	**Link of selection to pollution**	
Reproductive traits (6)	Reciprocal transplants (12)	Gene‐specific mRNA expression (16)	Experimental selection (27)	
	Protein level estimates (13)	mRNA expression (17)	Phenotype–environment correlations (28)	Genotype–environment correlations (29)
		Tests on known candidate loci (18)	Phenotype–genotype correlations (30)	
			Other (31)	
**Characteristics**				
Plasticity is not ruled out	Identification of traits and loci to be likely under selection		Investigation of adaptive changes/shifts	
Synchronic and/or allochronic	Genetic versus environmental bases for trait variation		Synchronic and/or allochronic	
Laboratory and/or field	Laboratory and/or field		Laboratory and/or field	
Mainly phenotypic surveys	Can be used to provide info prior to a population becoming subjected to selection		Random genetic drift is ruled out	

We converted all metal concentrations to parts per million (ppm) and created a numerical metric for the analyses: the natural logarithm of the metal concentration divided by the threshold concentration specific for each metal and habitat type determined by the Canadian Council of Ministers of the Environment (Supporting Information Table S3). When data were only provided in a graphical format, we used Getdata Graph Digitizer 2.26 (http://getdata-graph-digitizer.com) to estimate the displayed values. When sample size was given as a range, we calculated the average. The dataset included a large variety of experimental designs that were mainly classified as either “field” or “experiment” (Supporting Information Table S3). In some studies, the organisms were exposed to more than one metal under two scenarios. In the first scenario, populations were naïve to the particular metal tested, but they had been exposed to other stressors in their original habitats, while in the second case, the populations were sampled from sites contaminated by multiple metals and measured directly for their traits (e.g., size). When the contaminated sites contained more than one metal, we considered the metal with the highest concentration relative to the environmental quality guidelines. For studies involving more than one control and/or treatments, we used all possible combinations for the calculations of effect sizes. Calculation of the effect sizes and variances was performed in MetaWin Version 2 (Rosenberg, Adams, & Gurevitch, 2000) using Hedges' d. Effect sizes were then plotted against metal concentrations to assess the distribution of the data. Originally, we had 63 datapoints for weight, 89 for the number of offspring, and 260 for body metal content. However, because most of the studies reported data for more than one treatment (two or more distinct populations impacted by pollution, both sexes, subsequent generations) we had to compute a summary effect for the impact of pollution for all treatments combined. We formed a composite effects size for each study by performing a fixed‐effect meta‐analysis on the subgroups of each study with the following:

$$z=\frac{\text{Effect}\;\text{size}}{\text{Variance}};\;w=\frac{z}{\text{Effect}\;\text{size}};\;\text{Computed}\;\text{mean}=\frac{\text{Sum}\;\text{of}\;z}{\text{Sum}\;\text{of}\;w};\;\text{Variance}=\frac{1}{\text{Sum}\;\text{of}\;w}$$

Studies that had multiple datapoints because different concentrations of metals were tested were kept unchanged. We performed a multivariate mixed‐effect meta‐analysis using the package Metafor (Viechtbauer, 2010) with R 3.5.2. (R Core Team, 2018) to assess the relationship between effect sizes and metal concentrations on 10 studies (20 datapoints) for weight, 12 studies (38 datapoints) for the number of neonates and 17 studies (64 datapoints) for metal content in tissues. We used an information‐theoretic approach to rank statistical models based on Akaike information criteria (AICc; Burnham & Anderson, 2002). For each trait, we explored the null model, the random model, and then models with one, two, and three fixed terms (metal concentration, metal, habitat, phylum, subclass, presence of other metals, and experiment/field factor) for a total of 20 models. Models were ranked according to decreasing values of AICc (Supporting Information Table S4). Study ID was always considered a random term, and the random effects for the different metal concentrations tested within the same study were correlated through a multivariate parameterization. The models with the lowest AICc were visually inspected with residual plots to assess deviations from homoscedasticity and normality. We calculated heterogeneity (Tau‐squared and residual heterogeneity; Deeks, Higgins, & Altman, 2008) and intraclass correlation coefficients (*ρ*) to calculate the variance components for the between‐study and within‐study heterogeneity. We also recorded the number of generations studied in the subset of papers used for the meta‐analysis (*n* = 108). Finally, we mapped all study sites to show where pollutants have been studied around the world. For the meta‐analysis, we used the R packages metaphor (version 2.1; Viechtbauer, 2010), while for the world map, we used the packages rgdal (version 1.3‐6; Bivand, Keitt, & Rowlingson, 2018), rworldmap (version 1.3‐6; South, 2011), and ggplot2 (version 3.0; Wickham, 2016) in R version 3.0.2 (R Core Team, 2014).

## 3 OVERVIEW OF THE STUDIES

### 3.1 Type of pollution and geographic distribution of species

**Figure 2:**
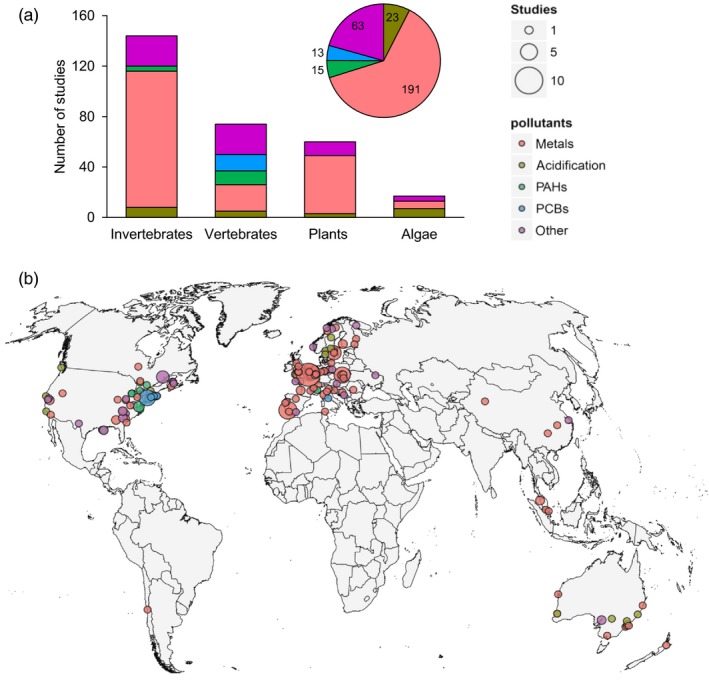
(A) Number of studies sorted by type of pollution and by taxa. (B) World map showing the localization of the contaminated sites from which populations were sampled. Different colors identify different types of pollution. Articles that made use of laboratory cultures were not considered

We found that 63% of the reviewed studies (*n* = 191) focused on metal pollution (Figure 2a) and more than half of metal studies (*n* = 108) were on invertebrates, followed by vertebrates (*n* = 74), plants (*n* = 60) and algae (*n* = 17). Specifically, metals were the most studied pollutants for terrestrial arthropods and to a lesser extent for terrestrial annelids, aquatic arthropods, and mollusks. Plants were also studied primarily in relation to metals. The effect of acidification on algae, invertebrates (Echinodermata), and vertebrates was investigated in 8% of the studies. Polychlorinated biphenyls (PCBs) and polycyclic aromatic hydrocarbons (PAHs) were the least represented (9%) with a dozen studies almost exclusively conducted on fish. The rest of the studies (~20%) focused on pollutants like pesticides, dioxin‐like compounds, radiation, waste heap, tributyltin, and other chemical compounds and were grouped together as “other” (Supporting Information Figure S1). These types of pollutants were studied mostly in aquatic arthropods and mollusks but also in vertebrates and plants.

Most studies were situated in Europe and eastern North America, which historically have been the most industrialized areas of the world (Figure 2b). Australia and central China were also relatively well sampled, while regions in South America, Africa, and South China experiencing high levels of pollution showed a paucity of data. In Europe, the most studied areas included Northern France and Poland but also Portugal and England. The geographic distribution of studies is biased to historical centers of industrial activity but does not reflect the current geographic distribution of contamination.

**Figure 3:**
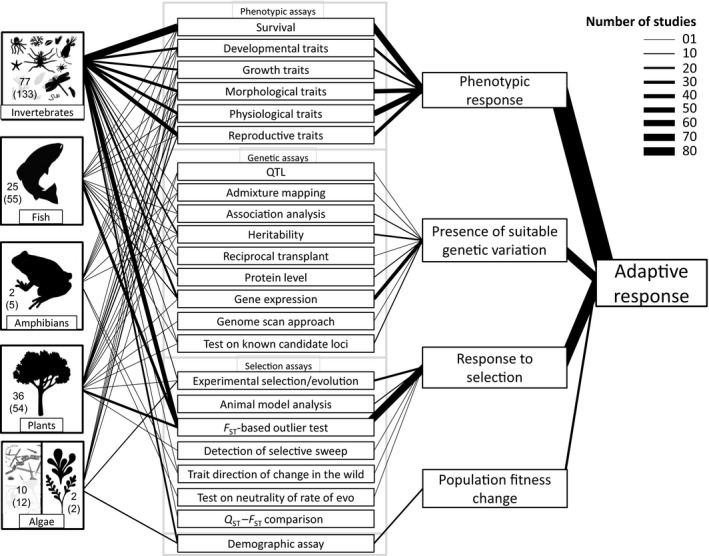
Number of studies on the different taxa that, through different approaches (phenotypic, genetic, selection, and demographic assays), found evidence for an adaptive response. The width of the lines represents the number of studies that belong to each approach. The numbers inside the boxes represent the number of species and, in brackets, the number of papers

Relatively few species were tested within each phylum (Figure 3, Supporting Information Figure S2): 77 species of invertebrates were investigated by 133 articles; 36 species of plant by 54 articles; 25 species of fish by 55 articles, two species of amphibian by five articles, 10 species of microalgae by 12 articles, and two species of macroalgae by two articles. Not surprisingly, the use of model species such as *Fundulus* (fish: Actinopterygii), *Chironomus* (invertebrate: Insect), *Daphnia* (invertebrate: Branchiopoda), *Arabidopsis* (plant: Eudicots), *Orchesella* (invertebrate: Entognatha) was common. These species were so extensively studied that they have been the subject of several important literature reviews. For example, Weis (2002) assessed what is known about tolerance and mechanisms of tolerance to several pollutants (mercury, dioxins, PCBs, and PAH) in different populations of killifish; Whitehead, Clark, Reid, Hahn, and Nacci (2017) reviewed key features of killifish populations and the genetic architecture underlying adaptive responses in these populations. Studies of selection on transcriptional regulation of the collembolan *Orchesella cincta* were reviewed by van Straalen and Roelofs (2005) in relation to cadmium contamination; Pauwels, Willems, Roosens, Frerot, and Saumitou‐Laprade (2008) compared molecular genetic results with other approaches (e.g., QTL analysis) and discussed the nature of the genes potentially involved in the adaptation to zinc‐polluted soils in plants. Studies with model species have built the foundation of our knowledge on micro‐evolutionary responses to pollution. Here, our goal was to synthesize the evidence for adaptive responses across multiple levels (genetic, individual, and population levels), in a range of model and nonmodel taxa and for a number of pollutants.

Phenotypic assays included the study of survival in different concentrations of pollutants (e.g., LC50), changes in development (e.g., hatchability), growth (e.g., growth rate), morphology (e.g., body size, leaf size), physiology (e.g., feeding rate), and reproductive traits (e.g., age at first brood, number of offspring). Survival and reproductive traits were the most studied in invertebrates, while in plants and vertebrates all traits were more or less equally studied (Figure 3). Genetic and selection assays included both quantitative trait studies and molecular genetic studies (Table 1). Genetic assays were encountered in 95 articles and were more prevalent in studies on invertebrates, where they were more common than selection assays and led also to more statistically significant findings than tests for selection. For example, for the invertebrates, there was a widespread use of gene expression techniques followed by heritability estimates. In plants, genetic assays were not very common and these studies were almost absent in algae. Gene expression studies were the most frequent genetic assay (*n* = 34) followed by studies on trait heritability (*n* = 16). Selection assays were the second most abundant assays (*n* = 121) after phenotypic assays. Studies on invertebrates were the most common followed by vertebrates and plants. *F*_ST_‐based outlier tests were the dominant way to find evidence for selection. Together with survival, these tests were the most commonly used to study adaptive responses of organisms in polluted conditions. Among the studies that found evidence of selection, the majority (86.5%) tested also for a link between responses to selection and the environment: 15.7% found evidence for genotype–environment correlations and another 15.7% for phenotype–environment correlations (Supporting Information Table S2). A number of studies (*n* = 31) on invertebrates and microalgae included population growth rate estimates.

### 3.2 EVIDENCE FROM PHENOTYPIC, GENETIC, SELECTION, AND DEMOGRAPHIC ASSAYS

**Figure 4:**
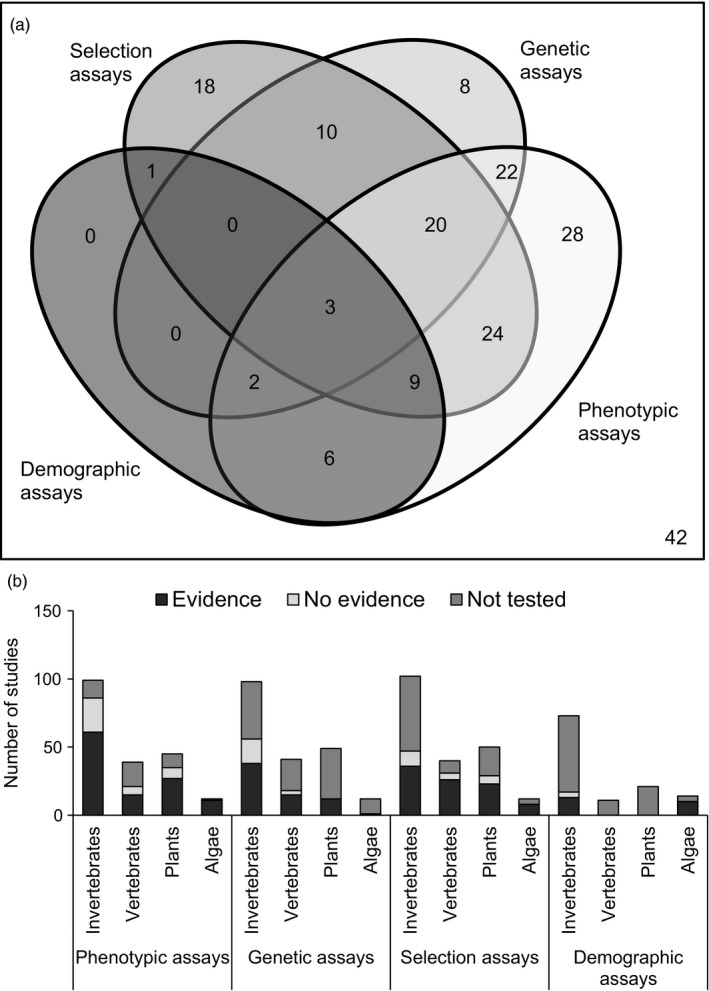
(a) Number of studies using different assays (phenotypic, genetic, selection, and demographic) that found evidence of a phenotypic response, presence of suitable genetic variation, a response to selection and population fitness change. (b) Number of studies on invertebrates, vertebrates, plants, and algae that found statistically significant evidence (or lack of) for a phenotypic response due to pollution (phenotypic assays), presence of genetic variation for resistance (genetic assays), responses to selection (selection assays), and population fitness changes (demographic assays). Number of studies in which these components were not considered are also shown (down right)

After grouping the results from different articles, we obtained 198 studies (Figure 4), 63% of which found significant differences in responses to pollution. Figure 4 shows the breakdown of studies across methodological approaches. Forty‐two studies (21%) did not find statistical differences among treatments, regardless of the approach used. Only three studies (Dutilleul et al., 2013; Dutilleul et al., 2014; Dutilleul, Goussen, B., Bonzom, J.‐M., Galas, S. & Réale, 2015; Kelly, Padilla‐Gamiño, & Hofmann, 2013; Messiaen, De Schamphelaere, Muyssen, & Janssen, 2010; Messiaen, Janssen, Thas, & Schamphelaere, 2012; Messiaen, Janssen, De Meester, & De Schamphelaere, 2013) found evidence of a phenotypic response to pollution with an underlying advantageous genetic basis and also found the presence of a response to selection followed by a demographic response. These studies provided the most thorough insights on the adaptive potential of the studied organisms.

Most of the studies finding adaptive responses came from phenotypic assays in invertebrates, particularly studies on survival (*n* = 47) followed by physiological traits (*n* = 26) and morphological traits (*n* = 20; Figure 4b). The evidence for phenotypic responses to pollution was often accompanied by evidence from molecular genetic approaches, making a total of 130 out of 198 studies (Figure 4a). Twenty studies included data from phenotypic, genetic, and selection assays and found statistical evidence for all three. However, only three studies assessed whether the observed resistance was heritable (Macnair, Smith, & Cumbes, 1993; Shirley & Sibly, 1999; Xie & Klerks, 2003) while only four assessed whether the specific trait studied (e.g., larval size, net reproductive rate) was heritable (Foo, Dworjanyn, Poore, & Byrne, 2012; Kelly et al., 2013; Messiaen et al., 2012; Sunday, Crim, Harley, & Hart, 2011); in all the cases, the traits were heritable. Macnair et al. (1993) studied the heritable variation in the degree of copper tolerance in *Mimulus guttatus* seeds collected from an abandoned copper mine (California). They found that populations from contaminated soil and some populations sampled downstream of the mine had 100% tolerance while populations sampled upstream showed variable tolerance that was related more to geographic location than copper concentration in the soil. Through the study of life‐history traits during experimental selection, they were able to demonstrate that tolerance was heritable and widespread in populations from contaminated soil due to beneficial genetic variation. Shirley and Sibly (1999) conducted a 20‐generation selection experiment using *Drosophila*, where they measured fecundity and many other traits during exposure to cadmium. Individuals from contaminated cultures developed resistance and had a higher fitness than the controls, and the evolution of resistance was due to a single sex‐linked gene. Xie and Klerks (2003) conducted a selection experiment for six generations to investigate the response to selection by cadmium in *Heterandia formosa*. The authors observed an increased resistance in the selection lines and found a heritability of 0.50. By calculating the heritability and testing the survival of six generations of controls and selected individuals, they provided compelling evidence for the evolution of resistance in a vertebrate population.

Three studies provided a complete assessment of the adaptive potential of the aquatic microcrustacean *Daphnia magna* (Messiaen et al., 2010; Messiaen et al., 2012; Messiaen et al., 2013), the free‐living soil nematode *Caenorhabditis elegans* (Dutilleul et al., 2013; Dutilleul et al., 2014; Dutilleul et al.,^2015^), and the sea urchin *Strongylocentrotus purpuratus* (Kelly et al., 2013). Messiaen et al. (2010) used laboratory cultures of *D. magna* to study the response to cadmium and temperature. Through life‐history trait analysis, they found that chemical pollution can affect genetic variation and between‐trait correlations. The response to other stressors (e.g., temperature) was also affected by pollution. Moreover, Messiaen et al. (2012) estimated additive and nonadditive components of the genetic variability of net reproductive rate during cadmium and temperature stress and uncovered a substantial level of stress, which translated into a decrease in the population mean reproductive rate. Broad‐sense heritability and total genetic coefficients of variation suggested a genetic determination of net reproductive rate. Clonal selection on this trait could positively influence population mean fitness. Additionally, they suggested that both asexual and sexual reproduction phases in the life cycle of *Daphnia* could play a role in the long‐term adaptive potential of populations to cadmium stress. Finally, Messiaen et al. (2013) measured reproductive performances of hundreds of clones from naïve populations and compared them with the laboratory cultures used by Messiaen et al. (2010) and Messiaen et al. (2012). They found that although there was no significant difference in the initial tolerance of clones, estimates of broad‐sense heritability of cadmium tolerance suggested great variation ranging from not significantly different from 0 to between 0.48 and 0.81. The authors stated that “it's difficult to predict the long‐term response to chemical pollution of unstudied populations from tolerance data on a sample of other populations,” suggesting that methods for forecasting long‐term responses (e.g., predictive models based on population genomic and tolerance time‐series data) are needed.

Dutilleul et al. (2013), Dutilleul et al. (2014), Dutilleul et al. (2015) conducted a series of studies on laboratory cultures of the nematode *C. elegans*. In their initial study, Dutilleul et al. (2013) studied uranium stress and its effect on phenotypic traits like survival, generation time, brood size, body length, and body bend. They found that at low concentrations of uranium, negative effects were reduced, but at high concentrations, negative effects were amplified across generations. Acclimation was not enough to ensure survival. Subsequently, Dutilleul et al. (2015) studied the genetic basis of survival, fecundity, and growth under uranium and salt stress while also estimating the heritability of these traits. Surprisingly, the most heritable traits in the control environment (fecundity and early growth) had a reduced heritability in the uranium‐contaminated environment. This reduction in heritability, possibly due to differences in gene expression of tolerance genes (e.g., metallothionein), was not proportional to the decrease in population fitness, and this could have impeded selection from acting on phenotypic traits. The authors concluded that by altering the genetic structure of populations, pollution can influence their potential to adapt to other stressors.

Kelly et al.’s (2013) study on the sea urchin *S. purpuratus* was the only individual study that employed all four approaches we advocate for here, albeit indirectly (Figure 4). The effects of acidification were studied using estimates of additive genetic variance for body size under high pCO_2_ across populations. The authors used these data to parameterize a model predicting the rate of evolution under changing pCO_2_ and the effect of evolutionary change on demographic rates. Their model showed that when selection on body size was weak, there was very little evolutionary change, but the impact of genetic variation became stronger with increasing selection intensity. When inclusion of population processes to experimental designs is challenging (e.g., due to long generation time), mathematical models can be crucial for strong inferences about the long‐term effects of pollution on fitness.

## 4 INVERTEBRATES AND METALS: A META‐ANALYSIS

We tested the relationship between metal concentration and effect sizes of weight, number of neonates, and body metal content. We accounted for the phylum and subclass of the studied organism, the habitat, the type of metal, and other factors such as the presence of other metals in the original habitat and the study type (laboratory experiment or field monitoring). We expected that body weight and number of neonates would decrease with increasing metal concentration and that body metal content would increase.

**Figure 5:**
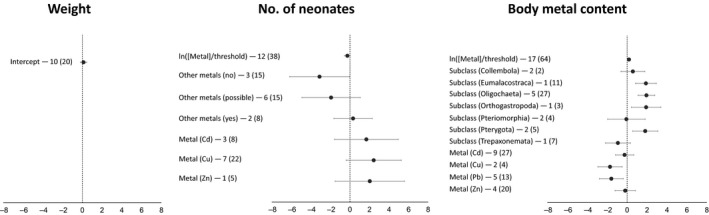
Fixed effects estimates and confidence intervals of AICc‐best models for weight, number of neonates and body metal content. The number of articles is shown beside each term and in brackets there is the number of datapoints

Surprisingly, we did not find a strong effect of metal contamination (Figure 5; Supporting Information Table S5). For body weight, which was the trait with the smallest dataset (10 studies and 20 datapoints), the best‐fit model was the random‐effect model (AICc = 32.41), which provided a small and nonsignificant mean effect size (0.12, *SE* = 0.16; Supporting Information Figure S5). For the number of neonates, the best‐fit model included the metal concentration (ln[ppm]/threshold), the presence of other metals, and the metal in question (AICc = 118.5). Metal concentration effect was weak (Figure 5), but the presence of other metals suggested a negative effect on the number of neonates produced, with lower numbers in the presence of other metals. Metal type (Cd, Cu, and Zn) had also a negative effect with copper being the strongest. For body metal content, the best‐fit model included the metal concentration, the metal type, and the taxonomic subclass (AICc = 175.05), and in this case, the metal concentration effect was slightly statistically significant. However, these results are to be interpreted with caution given the limited size of the datasets. In fact, the interclass correlation coefficients (*ρ*) were quite large for body weight (0.89; Supporting Information Table S5), indicating that the effects within studies (different metal concentrations tested) were highly correlated. Residual variabilities were large too, indicating that other unmeasured factors were contributing to the effect sizes.

The fact that we did not find strong relationships between the response variables and metal concentration suggests several issues. First of all, the power of our analysis was likely small given the limited number of studies and datapoints available. Moreover, the high heterogeneity of methods, factors tested, and types of experiments made comparisons very difficult. This issue was encountered by Oziolor et al. (2016) when attempting a meta‐analysis of evolutionary events in response to PAHs and PCBs. They found “a complexity and diversity in the academic investigations of population‐level ecotoxicological impacts that make it difficult to directly compare across studies” (Oziolor et al., 2016). Moreover, the different bioavailability of metals likely played a role in the heterogeneity of results we observed (De Coninck et al., 2013). Bioavailability of metals depends on a large variety of chemical, environmental, and biological parameters. Factors such as pH and acid‐buffering capacity, temperature, presence of organic matter or minerals, element speciation, concentrations of other substances can all play a role in the availability of a metal. Thus, the processes affecting bioavailability are heavily influenced by the type of habitat and are expected to change over time and among different organisms (John & Leventhal, 1995). Another important issue is that different individuals and cohorts within a population might have distinct strategies for coping with pollutants. The difference in effect size that we found across subclasses can be explained by the fact that traits such as weight and number of offspring may change in opposing directions during stress, depending not only on the intensity of the stress, but also on other biotic and abiotic conditions. For example, Amorim, Pereira, Soares, and Scott‐Fordsmand (2017) measured survival, reproduction, size, and metallothionein gene expression during a 3.5‐year selection experiment with *Folsomia candida *exposed to cadmium. They found body size was smaller in animals exposed to EC10 than EC50 concentrations. Body size is a complex trait that changes as a result of metal toxicity, detoxification costs, and shifts in energy allocation (Grześ, Okrutniak, & Woch, 2015) and is often a compromise of all the above (Kozłowski & Gawelczyk, 2002). The number of neonates is predicted to be low during stress, and it is often linked to large egg size as optimality models of life‐history theory predict (Lloyd, 1987; McGinley, Temme, & Geber, 1987; Sibly & Calow, 1986). Winkler and Wallin (1987) have also demonstrated that these traits are closely correlated. The number of offspring is also an adaptive compromise during stress given that larger eggs ensure a greater chances of survival and faster development (Fox & Czesak, 2000) while numerous small eggs ensure higher fecundity (Bernardo, 1996). As expected from optimality models, we found a general decrease in the number of offspring, although this was not statistically significant. The body metal content response showed a slight but not significant positive correlation with metal concentration. However, an observation of low body metal content as described in other studies (Donker, Raedecker, & Straalen, 1996) might indicate an adaptive response such as increased detoxification ability (Sibly & Calow, 1986) or decreased metal uptake (Harper, Smith, & Macnair, 1997).

Another potential reason for the weak effects we found is that effects of metals might be difficult to disentangle from other factors. For example, Kozlov and Zvereva (2011) conducted a meta‐analysis on primary producers (bryophytes, vascular plants), primary and secondary consumers (arthropods), and decomposers (fungi, arthropods) with the aim of revealing regional and global pattern from small‐scale observational studies. They found that the effect of pollution depended on the pollutant (type, amount, duration of exposure), the organism (life cycle, life history), the level of biotic organization at which the response was measured (organism, population, community), and the environment (biome, climate). Moreover, the effect of one factor was often modified by other factors with many interactions among them. Overall, the magnitude of responses to pollution was weak, and trophic level, type of pollution, and biome explained only 7% of the variation (Kozlov & Zvereva, 2011).

Predicting the outcome of adaptive allele dynamics in a changing environment is generally very challenging given fitness × environment interactions, and variable responses mechanisms and rates across taxa (Milesi, Lenormand, Lagneau, Weill, & Labbé, 2016; Morgan, Kille, & Stürzenbaum, 2007). There is a clear opportunity to improve and build on the dataset we have assembled here. Future meta‐analyses will have the task of accounting for a complex set of predictors and confounding variables.

## 5 SUMMARY

Generally speaking, evidence for adaptive responses to pollution requires the demonstration of increased heritable resistance to relevant environmental pollutants. When assessing both individual and population studies, our review found relatively modest support for long‐term adaptive responses to pollution.

A handful of the reviewed studies demonstrated that including measures of population growth rate often reveals how pollution can negatively affect population trends despite the presence of tolerant phenotypes (Anderson, Kille, Lawlor, & Spurgeon, 2013; Dutilleul et al., 2014; Haimi et al., 2006; Medina, Morandi, & Correa, 2009; Postma & Davids, 1995). Studies that combined several laboratory approaches (demographic assays with quantitative trait methods and molecular genetics) provided clearer evidence for adaptive responses to pollution. These studies also found that a successful adaptive response to pollution can be altered by another stressor like temperature or increased salinity (Dutilleul et al., 2014; Messiaen et al., 2012). They also suggest that it is not generally possible to extrapolate the findings from specific laboratory populations to other populations of the same species in the field.

Once observation of resistance to pollution has been made, we suggest that compelling evidence for adaptive changes in the field requires several additional pieces of information: (a) demonstration that the changes in the trait studied are genetically determined and are subject to natural selection; (b) assessment of potential confounding environmental variables; (c) the demonstration that the increase in adaptive trait value can sustain a positive population growth rate and thus the long‐term persistence of the population (Hansen et al., 2012). Field samples should always be accompanied by a complete ecological analysis of the soil/sediment/water from which organisms are obtained. An extra effort should be made to determine the bioavailability of the pollutant in question (De Coninck et al., 2013). In the case of laboratory studies, repeatable and highly controlled ecotoxicological tests should be accompanied by multi‐generation experiments in which population growth rate is estimated. Additionally, if suitable molecular markers are available, in‐depth assessment of genetic structure and genetic variation for the most advantageous traits should be attempted (Figure 1).

Besides a scarcity of demographic assays, we also found several sources of biases in the literature. These include publication, taxonomic, and methodological biases. The latter includes the lack of standardized methodologies among studies of similar species, studies covering only one generation (Supporting Information Figure S4) and studies focused on only a single life stage of the studied organisms (Table 2).

**Table 2:** The reviewed results might be subject to biases such as publication bias, nonindependence of studies; dominance of laboratory studies; poorly standardized methodologies; few generations covered during experiments; limited and noncomparable life stages investigated

Factor	Bias	Description
Type of results	Publication bias	Positive results tend to be published more than negative ones. Publication bias is a common issue in the scientific literature, and it may lead to distorted findings in systematic reviews and meta‐analyses
Type of study	Only laboratory study	Almost all experiments on adaptive responses to pollution were conducted under laboratory conditions. In some cases, rearing certain species under laboratory conditions was not possible and few studies used microcosms in the original natural habitats (Bahrndorff, Ward, Pettigrove, & Hoffmann, 2006; Piola & Johnston, 2006)
Approach	Lack of standardization of methodologies and parameters within studies of similar species	Studies are characterized by a range of methodologies and different combinations of measurements and observations. Methods are taxon‐specific, and even within the same general methodology, there are major differences in duration of the experiment and concentrations tested among studies
Choice of populations	Comparison of populations already established in the field	Comparing populations from historically known polluted habitats and populations sampled from reference habitats give rise to problems concerning the unknown genetic history of the populations studied, the processes behind it and the fact that sensitive species may just disappear before investigations
Number of generations covered in an experiment	Only one or few generations	Most of the experiments looked at metal effects over few generations (Supporting Information Figure). Fifty studies among 108 remained vague regarding the number of generations covered. A handful of studies covered 8–10 generations (Fisker, Sørensen, Damgaard, Pedersen, & Holmstrup, 2011; Leon Paumen, Steenbergen, Kraak, Straalen, & Gestel, 2008; Postma & Davids, 1995; Vidal & Horne, 2003; Ward & Robinson, 2005), and only two covered more than ten generations (Kafel, Zawisza‐Raszka, & Szulińska, 2012; Shirley & Sibly, 1999)
Age class	Using only one life stage	The susceptibility to toxic substances depends on the life stage of an organism. Initial structure of a population in an experiment influences its susceptibility to pollutants. The exploration of only early life stages excludes the investigation of reproductive traits

## 6 CONCLUSIONS

Despite decades of active research, it is still difficult to make broad statements about how likely, or how common, population adaptation is in the presence of environmental contamination. Given the challenges of predicting the adaptive response of wild, populations based on data from a handful of populations or laboratory cultures with model organisms, we stress the need for: (a) long‐term monitoring programs of populations in polluted habitats that integrate demographic studies with phenotypic, genetic, and selection assays; (b) use of standardized protocols among studies of similar species to make evolutionary toxicology studies more comparable (Oziolor et al., 2016); (c) an effort to deepen our understanding of evolutionary processes and underlying genetic mechanisms of resistance. Such approaches provide a great potential to advance our understanding of evolution in response to pollution in wild populations.

## DATA AVAILABILITY

Data for this study will be available at Fig Share: 10.6084/m9.figshare.7730255.

## CONFLICT OF INTEREST

None declared.
