# Supplementary material for: Mixed evidence for adaptation to environmental pollution
Source: Evol Appl. 2019 Apr 9;12(7):1259–73. doi: 10.1111/eva.12782 (PMC6691217; doi:10.1111/eva.12782)
Supplement: Supplementary file 1 [file EVA-12-1259-s001.docx]

**Mixed evidence for adaptation to environmental pollution**

Alessandra Loria^1^, Melania E. Cristescu^1^, Andrew Gonzalez^1^

*Department of Biology, McGill University, Montreal, QC H3A 1B1, Montreal, Canada*

**Correspondence**: Alessandra Loria

^1^ Department of Biology, McGill University, 1205 Dr Penfield, Stewart Biology Building, Montreal, QC, Canada H3A 1B1; e-mail: alessandra.loria@mail.mcgill.ca

**Supporting information**:

The use of molecular markers

Table S1

Table S2

Table S3

Table S4

Table S5

Fig. S1

Fig. S2

Fig. S3

Fig. S4

**The use of molecular markers**

Molecular markers represent an essential tool in studies on evolutionary change (Klerks et al., 2011). They can be used for the assessment of the genetic diversity and genetic structure at the population level, to evaluate the potential to respond by adaptation, and to study the evolution at specific loci under selection (Hoffmann & Daborn, 2007; Hoffmann & Willi, 2008). The pros and cons of using molecular markers in eco-toxicology have been extensively reviewed (Hoffmann et al., 1995; Belfiore & Anderson, 1998; Belfiore & Anderson, 2001; Hoffmann & Daborn, 2007; Monserrat et al., 2007; Hoffmann & Willi, 2008).

Shifts in allele frequencies at particular loci are one of the most obvious consequences of directional selection (Hoffmann & Daborn, 2007). The assessment of allozyme frequency differences between impacted and non-impacted populations was very common especially in the studies carried out between 1992 and 1997 (Fig. S3). Their use was also common between 1998 and 2003 to follow a sharp decline between 2004 and 2014. Non-specific techniques such as RAPD (random amplified of polymorphic DNA) and RFLP (restriction fragment length polymorphism) surveys were represented especially in the period between 1998 and 2003 with a boom for RAPD studies and 2004-2009 for RFLP. The assessment of genetic diversity through the use of microsatellite markers increased through time thanks to their high levels of polymorphism and ease of application. Early attempts (2004-2009) in identifying genes underlying trait variation were done by the use of molecular tags such as amplified fragment length polymorphisms (AFLP) characterized by a large number of polymorphisms per samples, and single nucleotide polymorphisms (SNPs), distinguished for their high accuracy and large number (2004-2014). The investigation of regulatory changes under control and stressful conditions was another approach used to study adaptive alterations at the level of gene expression. The use of DNA microarrays in relation to pollution prevailed between 2004 and 2009 and the studies were differentiated into the ones that targeted a specific region of the genome for its known role in tolerance and studies that looked at the genome-wide gene expression in order to identify genes involved in responses to pollutants. The majority of the studies that targeted a specific region of the DNA looked at metallothionein genes which encode for cystein-rich proteins involved in the regulation of metal concentrations in the cell playing an important role of detoxification and protection against toxic substances. For example, Sterenborg and Roelofs (2003) and Timmermans et al. (2005) looked at metallothionein expression on the springtail *Orchesella cincta* and found a higher level of expression of these genes in populations from metal contaminated environments. Several studies found evidence of selection on metallothionein genes by looking directly at changes in allele frequencies (Timmermans et al., 2007). Additionally, other studies directly measured metallothionein protein concentrations in different tissues of invertebrates and fish and found that the concentration of these proteins was correlated with contamination levels (Moraga et al., 2002) or higher in the tissue of individuals from contaminated habitats (Ross et al., 2002; Knapen et al., 2004). Similar progress in the understanding of the mechanistic basic of resistance has been made in relation to PCBs and dioxin-like contaminants in fish (Hahn et al., 2004; Wirgin et al., 2011). These types of toxicities are known to be mediated by the aryl hydrocarbon receptor (AHR) which is a ligand-activated transcription factor through which contaminants cause altered gene expression (Hahn et al., 2004). Several studies have compared the structure and expression of these molecules between impacted populations and non-impacted populations reviewed in (Wirgin & Waldman, 2004).

Overall, there was inconsistency among studies that used markers such as allozymes and microsatellites. Despite their relatively high polymorphism levels, they did not always succeed in providing results that reflected predictions (i.e. decreased genetic diversity in impacted populations, shifts in allele frequencies correlated to contamination). Even when predictions were confirmed, links between patterns and causes were not generally easy to demonstrate and remained unknown (Ford, 2002). As argued by Belfiore & Anderson (2001) and Staton et al. (2001) many other factors may contribute to the genetic changes observed. Mutations, gene flow, genetic drift represent other sources of these changes whose interplay influences the phylo-geography of species' distribution. Population declines due to the detrimental effects of contaminants can also play a substantial role in determining the genetic structure of impacted populations leading to a remarkable random genetic drift.

The identification of candidate genes that influence the variation in particular traits and that undergo changes in allele frequency in response of environmental change is indispensable for a deep understanding of the mechanisms of selection and adaptation (Hoffmann & Daborn, 2007; Hoffmann & Willi, 2008) to pollution. Opportunities for the identification of genes under selection increased in the last years due to the recent advances in next generation sequencing techniques. Genomics, transcriptomics, and proteomics technologies will finally revolutionize discovery-based research thanks to their efficiency and relatively low costs (Morozova & Marra, 2008; Davey et al., 2011).

| **Table S1.** Summary table of the studies reviewed, with information about the species tested, the type of pollution studied, the type of the approach or response measured, the major findings, and whether the study took place in the field or under laboratory conditions. The type of pollution list can include metals that have been tested in the lab but also metals present in the sampling site. Field* means that the populations were sampled from the field and samples were sacrificed for molecular analyses. When the source is not specified, the findings refer to the populations from contaminated sites. | | | | | |
| --- | --- | --- | --- | --- | --- |
| **Organism** | **Type of pollution** | **Type of response measured** | **Main findings** | **Lab or field** | **Reference** |
| **PLANTS** |  |  |  |  |  |
| **Angiosperms** |  |  |  |  |  |
| *Acer pseudoplatanus* | Heavy metals (Cu) | Growth of callus tissue | Persistence metal tolerance trait (also after growing in uncontaminated media) | Lab | Turner and Dickinson, 1993 |
| *Acer rubrum* | Heavy metals (Cd, Co, Cu, Ni) and P | Life-history traits: above-ground size, base stem diameter, dry mass, total leaf area, stem diameter, rooting depth, dry mass, leaf dry matter content (LDMC), turgescent fresh mass, specific leaf area (SLA) | Decreased growth in uncontaminated substrates but small difference between controls | Outdoor with experimental sand | Kirkey *et al.,* 2012 |
| *Arabidopsis arenosa* | Heavy metals (Cd, Pb, Zn) | Biometric measurements: # of leaves/plant, length and width of 5 leaves of each plant, height of the plants, # reproductive shoots and flowers/plant, length of 5 siliques/plant, # seed/silique, weight of seeds, root test, cytogenetic analysis | Significant morphological differences, higher tolerance in the pop from contaminated site | Field and lab | Przedpelska and Wierzbicka 2007 |
| *Arabidopsis sp.* | Radiation (Chernobyl), other mutagens | Transient recombination assay, expression of radical scavenging and DNA-repair genes, genome methylation | Lower frequency of extrachromosomal homologous recombination, significant differences in radical scavenging and DNA-repair genes expression, higher level of methylation in the pop from contaminated site | Lab | Kovalchuk *et al.,* 2004 |
| *Arabidopsis halleri* | Heavy metals (Zn) | Transcriptomics, real time RT-PCR | High constitutive expression of metal homeostasis genes in the shoots, differences between root and shoot transcript profile. Transcript abundance higher than *A. thaliana* | Lab | Becher *et al.,* 2004 |
| *Arabidopsis halleri* | Heavy metals (Zn) | Quantitative trait loci (QTL) analysis | High broad sense heritability, 5/70 significant QTLs | Lab | Frérot *et al.,* 2010 |
| *Arabidopsis halleri* | Heavy metals (Cd, Zn) | Sequencing of 13 DNA segments across the HMA4 region, quantitative PCR | Enhanced gene product dosage. Higher transcript level than *A. Thaliana* | Lab | Hanikenne *et al.,* 2013 |
| *Arabidopsis halleri* | Heavy metals (Zn) | RNA interference to downregulate HMA4 (HeavyMETAL ATIPASE4) expression | Zn hyperaccumulation and full hypertolerance to Cd and Zn depends on the metal pump HMA4 (combination of modified cis-regulatory sequences and copy number expansion in comparison to *A. Thaliana)* | Lab | Hanikenne *et al.,* 2008 |
| *Arabidopsis halleri* | Heavy metals (Cd, Pb, Zn) | Molecular markers (AFLP), neutrality tests | Four loci departed from neutrality | Lab and field* | Meyer *et al.,* 2009 |
| *Arabidopsis halleri* | Heavy metals (Zn) | Molecular markers (RFLP on chloroplastDNA) | No founding effects, similarity between close pop instead of adapted or reference | Lab and field* | Pauwels *et al*., 2005 |
| *Arabidopsis halleri* | Heavy metals (Cd, Pb, Zn) | Molecular markers (5 microsats) | High clonal diversity, limited seed dispersal. Clonal spread was more extensive in the lowly polluted zone but no evidence of genetic divergence due to heavy metal heterogeneity | Lab and field* | Van Rossum *et al.,* 2004 |
| *Arabidopsis halleri* | Heavy metals (Zn) | QTL analysis, Effective concentration (EC) 100, molecular markers (65 sequence-based markers and 18 AFLP) | At all QTL positions Zn tolerance was enhanced by *A. halleri* alleles | Lab and greenhouse | Willems *et al.,* 2007 |
| *Arabidopsis thaliana* | Heavy metals (Zn) | Transcriptomics, real time RT-PCR | Transcript abundances of several genes much higher in *A. Halleri* after 4 days of Zn exposure | Lab | Becher *et al.,* 2004 |
| *Arabidopsis thaliana* | Heavy metals (Cs) | Selection exp, life-history traits (root elongation, dry weight), Cs content, molecular markers (nptII gene conferring kanamycin resistance) | Decreased root elongation in the absence of Cs; ability to develop aerial organs in the presence of Cs (control did not) | Lab | Marmiroli *et al.,* 2009 |
| *Betula papyrifera* | Heavy metals (Cd, Co, Cu, Ni) and P | Life-history traits: above-ground size, base stem diameter, dry mass; total leaf area, stem diameter, rooting depth, dry mass, leaf dry matter content (LDMC), turgescent fresh mass, specific leaf area | Decreased growth in uncontaminated substrates | Outdoor with experimental sand | Kirkey *et al.,* 2012 |
| *Betula pubescens* | Heavy metals (Cu, Ni) | Survival (seeds), life-history traits (seedling height, length, chlorophyll fluorescence) | Reduced performance in pristine conditions | Greenhouse | Eranen 2008 |
| *Biscutella laevigata* | Heavy metals (calamine waste heap) | Metal uptake, mineral status, molecular markers (AFLP) | No significant change in metal uptake. Equal genetic variability | Lab and field* | Wasowicz *et al.* 2014 |
| *Biscutella laevigata* | Heavy metals (Pb, Zn) | Life-history traits (leaf length, width and color, degree of coverage of hairs on leaf, thickness of palisade and spongy mesophyl cells), root measurement, tolerance index | Growth was stimulated by Pb and Zn in pop from contaminated site | Lab and field | Wierzbicka and Panufnik 2004 |
| *Calamagrostis epigejos* | Heavy metals (Cd, Cu, Pb, Zn) | Life-history traits (root elongation, biomass of roots, rhizomes, shoots, and tb), tolerance index (TI) | Cu: significant higher TI, higher performance in high Cu in pop from contaminated site Tolerance: Cu > Cd > Zn > Pb | Lab (growth chamber) | Lehmann and Rebele 2004 |
| *Cynodon dactylon* | Heavy metals (Cd) | Molecular markers (67 microsats), turf quality, transpiration rate, chlorophyll content, leaf water content and growth rate | Wide phenotypic variation. The majority of accessions from the same/adjacent region clustered into the same groups | Lab and field* | Xie *et al.* 2014 |
| *Dianthus carthusianorum* | Waste heap (Pb, Zn) | Life-history traits (# of leaves, average leaf length and width, length of inflorescence), accumulation of proline, anthocyanins, and photosynthetic pigments. Molecular markers (17 RAPD and 4 ISSR) | Persistent significant differences in leaf size and shape and genetic differences | Lab and field* | Wójcik *et al.* 2013 |
| *Dianthus carthusianorum* | Heavy metals (Pb, Zn) | Life-history traits (leaf length and width, # of flowers per inflorescence). Root tolerance test | Lower weight of aerial parts, shorter and narrower leaves, smaller # of leaves/plant | Lab | Zalęcka and Wierzbicka 2002 |
| *Elodea nuttallii* | Heavy metals (Cd, Hg) | Gene expression with next generation sequencing (NGS) | Subset of metal responsive genes able to assess metal contamination | Lab | Regier *et al.* 2013 |
| *Elsholtzia haichowensis* | Heavy metals (Cu) | Metal content, chlorophyll content, electrolyte leakage, antioxidant enzyme assay | Chlorophyll content and electrolyte leakage less affected by Cu and lower Cu content in pop from contaminated site. Antioxidant enzyme activity was induced | Lab | Liu and Xiong 2005 |
| *Elymus repens* | Heavy metals (Cd, Cu, Pb, Zn) | Life-history traits (root elongation, biomass of roots, rhizomes and shoots), tolerance index (TI) | Zn: more tolerant than *C. Epigejos* Tolerance: Cu > Cd > Pb > Zn | Lab (growth chamber) | Lehmann and Rebele 2004 |
| *Eucalyptus calophylla* | Metals (Al), acidification | Survival, molecular markers (10 allozymes) | No significant differences in allele frequencies and no differences in survival | Lab and field* | Egerton-Warburton 1995 |
| *Eucalyptus patens* | Metals (Al), acidification | Survival, molecular markers (10 allozymes) | No significant differences in allele frequencies and no differences in survival | Lab and field* | Egerton-Warburton 1995 |
| *Eucalyptus rudis* | Metals (Al), acidification | Survival, molecular markers (11 allozymes) | No significant differences in allele frequencies and no differences in survival | Lab and field* | Egerton-Warburton 1995 |
| *Hordeum vulgare* | Heavy metals (Cd) | Enzyme activity | Induction of antioxidant enzymes and increase in the level of proteins | Lab | Patra and Panda 1998 |
| *Mimulus guttatus* | Heavy metals (Cu) | Life-history traits (length of roots), selection exp (known tolerance gene present) | Variation in root length detected at higher levels of copper is heritable | Lab | Macnair *et al.,* 1993 |
| *Mimulus luteus* | Heavy metals (Cu) | Life-history traits (root # and length) | Root # and length strongly inhibited in pop from reference sites | Lab | Ginocchio *et al.,* 2002 |
| *Plantago arenaria* | Heavy metals (Cd, Cu, Ni, Zn) | Metal content, root bending assay | Presence of constitutive tolerance except for Cu (adaptive) | Lab | Remon *et al.,* 2007 |
| *Poa annua* | Organic chemicals | Molecular markers (5 allozymes) | Higher # of heterozygous in the polluted sites | Lab and field* | Chen *et al.,* 2003 |
| *Prosopis sp.* | Heavy metals (Cu) | Survival, metal uptake, individual growth | Higher survival, faster growth and metal content in pop from contaminated site | Lab | Haque *et al.,* 2009 |
| *Sedum alfredii* | Heavy metals (Cd, Pb, Zn) | Metal accumulation, molecular markers (17 RAPD) | Reduced genetic diversity, high variation in metal accumulation | Lab and field* | Deng *et al.,* 2007 |
| *Silene dioica* | Heavy metals (Cu) | Mineral nutrient concentration, metal content, phenolic metabolism-related parameters, oxidative stress-related proteins, estimation of free aminoacids | Highest tolerance in pop from the locality with the highest soil Cu content (soluble proteins least affected, enhanced enzyme activity) | Lab | Kováčik *et al.,* 2010 |
| *Silene paradoxa* | Heavy metals (Cu) | Molecular markers (5 microsats) | Independent colonization events from serpentine populations, grouping of populations according to geographical location | Lab and field* | Mengoni *et al.,* 2001 |
| *Silene paradoxa* | Heavy metals (Cu, Ni) | Molecular markers (8 RAPD) | Distribution of genetic polymorphism related to the tolerance to Cu and location. Two bands exclusive to Cu-tolerant populations | Lab and field* | Mengoni *et al.,* 2000 |
| *Silene vulgaris* | Heavy metals (Cu) | Mineral nutrient concentration, metal content, phenolic metabolism-related parameters, oxidative stress-related proteins, estimation of free aminoacids | Higher tolerance in pop from contaminated sites | Lab | Kováčik *et al.,* 2010 |
| *Silene vulgaris* | Heavy metals (Cd, Cu, Zn) | Root growth (qualitative), cross tests | Few specific major genes involved | Lab | Schat *et al.,* 1996 |
| *Silene vulgaris* | Calamine waste heap | Life-history traits: leaf width, thickness, palisade and spongy mesophylls measurements, cell surface estimate, size of epidermal cells and # of stomata, individual growth rate. Fresh and dry weight of roots and shoots, metal content, root hairs | Faster growth, thicker and narrower leaves, trailing shoots of small diameters in pop from contaminated site | Lab and field | Wierzbicka and Panufnik 1998 |
| *Taraxacum officinale* | Heavy metals (Cd, Fe, Ni, Pb), airborne particulate matter (PM10) | Molecular markers (variable-number-tandem-repeat loci), metal content | Negative correlation between the # of genotypes at a site and increasing amounts of PM10, concentrations of five soil metals (Cd, Cu, Fe, Ni and Pb), leaf tissue levels of Fe | Lab and field* | Keane *et al.,* 2005 |
| T*hlaspi caerulescens* | Heavy metals (Cd, Zn) | Molecular markers (3CAPS and 7 microsats) | Genetic differentiation linked to heavy metal concentrations at some candidate loci (gene encoding metal transporter) | Lab and field* | Besnard *et al.,* 2009 |
| T*hlaspi caerulescens* | Heavy metals (Zn) | Molecular markers (4 isozymes) | Tolerance might have evolved twice in populations from different areas | Lab and field* | Koch *et al.,* 1998 |
| *Typha latifolia* | Organic and inorganic chemicals | Variable-number-tandem-repeat (VNTR) | Higher genetic diversity in the two most polluted sites | Lab and field* | Keane *et al.,* 1999 |
| *Viola tricolor* | Heavy metals (Cd, Pb, Zn) | ISSR PCR fingerprinting | Higher genetic polymorphism and gene diversity in pop from contaminated sites | Lab and field* | Slomka *et al.,* 2011 |
| **Bryophyta** |  |  |  |  |  |
| *Ceratodon purpureus* | Heavy metals (Cd, Cu, Pb, Zn) | Gametophytic growth, reproductive expression | Higher protonemal growth in pop near a smelter | Lab | Jules and Shaw 1994 |
| **Pynophyta** |  |  |  |  |  |
| *Picea abies* | Heavy metals (Pb, Zn) | Survival, molecular markers (8 isozymes) | Heterozygote frequencies at four loci increased in the surviving seedling | Lab and field | Bergmann and Hosius 1996 |
| *Picea abies* | Sulphur (S) | Molecular markers (10 isoenzymes) | Higher heterozygosity and genotypic polymorphism index in the tolerant pop | Lab and field* | Prus-Glowacki and Godzik 1995 |
| *Picea rubens* | Sulphate pollution (and climate change) | Molecular markers (33 SNPs and 9 microsats) | Three out of seven SNPs strongly associated with pollution class | Lab and field* | Bashalkhanov *et al.,* 2013 |
| *Pinus sylvestris* | Radionuclide | Aberrant cells in the root meristem of germinated seeds, reproductive ability (frequency of abortive seeds) | No consistent difference in reproductive ability | Lab and field* | Gera's kin *et al.,* 2011 |
| *Pinus sylvestris* | Radionuclide | Molecular markers (5 isozymes) | All indices of genetic variability increased with the dose absorbed | Lab and field* | Gera's kin *et al.,* 2010 |
| *Pinus sylvestris* | Fertilizers, organic dyes | Molecular markers (9 allozymes) | Lower genetic diversity close to the source of pollution | Lab and field* | Korshikov *et al.,* 2002 |
| *Pinus sylvestris* | Radiation (Chernobyl) | Molecular markers (222 AFLP) | 6% of loci (15/222) identified as candidates for selective responses | Lab and field* | Kuchma and Finkeldey 2011 |
| *Pinus sylvestris* | Heavy metals (Cd, Pb) | Molecular markers (18 isoenzymes), cytogenetic analysis | Higher # of genotypes in the sensitive pop but lower heterozygosity | Lab and field* | Prus-Glowacki *et al.,*2006 |
| *Pinus sylvestris* | Heavy metals (Cu) | Molecular markers (allozymes), metal content | Higher genetic variation than control pop | Lab and field* | Prus-Glowacki *et al.,*1999 |
| *Pinus sylvestris* | Oxides of Sulphur (S) | Molecular markers (9 isoenzymes) | Populations with a greater heterozygosity were more tolerant to pollution | Lab and field* | Wojnicka-Póltorak 1997 |
| **INVERTEBRATES** |  |  |  |  |  |
| **Annelida** |  |  |  |  |  |
| *Aporectodea caliginosa* | Pesticides | Weight, detoxification enzymes analysis, energy resources analysis | Activities of two enzymes increased with contamination. Pre-exposure accelerated activation of detoxification activities | Lab and field | Givaudan *et al.,* 2014 |
| *Aporectodea chlorotica* | Pesticides | Weight, detoxification enzymes analysis, energy resources analysis | Stress was reflected in depletion of energy reserves. Pre-exposure accelerated activation of the detoxification enzyme sGST towards epoxiconazole | Lab and field | Givaudan *et al.,* 2014 |
| *Aporectodea tuberculata* | Heavy metals (Cu, Zn) | Biomarkers: metallothionein (MT), Cytochrome P4501A (CYP1A) and glutathione-S-transferase (GST) | MT concentration and the other protein activities decreased with increasing distance from the smelter | Lab | Lukkari *et al.,* 2004 |
| *Cognettia sphagnetorum* | Heavy metals (Cu) | Survival, body size at reproduction, # fragments produced, individual growth rate, age of reproduction, age of death), pop growth, CO_2_ production, Molecular markers (9 allozymes) | Slower growth rate, fewer fragments of larger size, slower pop growth rate, reduced genetic diversity in the pop from contaminated site | Lab and field* | Haimi *et al.,* 2006 |
| *Cognettia sphagnetorum* | Heavy metals (Cu) | Survival, segments of worms, body size, density of worms | Higher survival in pop from contaminated site | Lab | Salminen and Haimi 2001 |
| *Dendrobaena octaedra* | Heavy metals (Cu, Zn) | Survival, weight, cocoon production, metallothionein level | No significant differences between contaminated and reference sites | Lab | Bengtsson *et al.,* 1992 |
| *Dendrobaena octaedra* | Heavy metals (Cu) | Gene expression of 6 genes potentially involved in resistance | Up-regulation in pop from contaminated sites | Lab | Fisker *et al.,* 2013 |
| *Dendrobaena octaedra* | Heavy metals (Cu) | Survival, life-history traits (mean weight, mean cocoon production per adult), estimates of growth rate, hatchability | Higher individual growth rate, reduced time to maturity, increased reproduction, and increased mortality of pop from polluted area in both control and polluted environment | Lab | Fisker *et al.,* 2011 |
| *Dendrobaena octaedra* | Heavy metals (Cd) | Hatching rate, Cd content, body mass changes, life-history traits (maturation and 1st repro, cocoon production, cocoon mass), survival analysis (longevity, survival time of F1, hazard rate, median life expectancy) | Heritable higher reproduction and higher survival | Lab | Rozen 2006 |
| *Dendrodrilus rubidus* | Heavy metals (Cu) | Survival, weight, earthworm condition index (Langdon *et al*. 1999; health assessment), metal content | Higher tolerance, higher metal content and less change in weight in pop from contaminated sites | Lab | Arnold *et al.,* 2008 |
| *Dendrodrilus rubidus* | Arsenic (As) | Comet assay (measurement of DNA damage), internal content and speciation | Very high levels of As observed in the treatments | Lab | Button *et al.,* 2012 |
| *Eisenia fetida* | Heavy metals (Cd, Pb, Zn) | Metal content. Gene expression of four genes | Response of Cd-mt gene and accumulation of Cd in worms consistent with [Cd] | Lab | Brulle *et al.,* 2011 |
| *Eisenia fetida* | Heavy metals (Cd, Pb, Zn) | Gene expression | Three candidate genes strongly induced in the treatments | Lab | Brulle *et al.,* 2008 |
| *Hediste diversicolor* | Heavy metals (Cu) | Survival, molecular markers (6 allozymes) | Specific alleles linked to lower mortalities | Lab | Virgilio and Abbiati 2004 |
| *Limnodrilus hoffmeisteri* | Heavy metals (Cd, Co, Ni) | Estimates of extra genetic variance, survival time | Single segregating genetic factor underlies the resistance to heavy metals | Lab | Martinez and Levinton 1996 |
| *Lumbricus castaneous* | Arsenic (As) | Comet assay (measurement of DNA damage), internal content and speciation | Very high levels of As observed in the treatments | Lab | Button *et al.,* 2012 |
| *Lumbricus rubellus* | Arsenic (As) | Survival, weight, food consumption, cocoon production rate, metal content, pop growth rate estimates, molecular marker (COI) | Three clades. Sensitivities changed based on life-history stages but not among clades. Risk of extinction at environmentally relevant concentration | Lab | Anderson *et al.,* 2013 |
| *Lumbricus rubellus* | Heavy metals (Pb, Zn) | Metal content and partitioning profile, molecular markers (COII) | Fewer haplotypes in the lineages from mine sites | Lab and field* | Andre *et al.,* 2010 |
| *Lumbricus rubellus* | Arsenic (As) and heavy metals (Cu) | Molecular markers (COI and AFLP), DNA methylation analysis | The association between metylation sensitive AFLP(me-AFLP) and soil As levels differed in the two lineages. Epigenetic mechanisms in lineage B and genetic processes in lineage A (no strong association between me-AFLP and As levels in soil) | Lab and field* | Kille *et al.,* 2013 |
| *Lumbricus rubellus* | Heavy metals (As) | Survival, cocoon viability, x-ray absorption spectra | Higher resistance and cocoon viability in offspring from As-contaminated sites | Lab | Langdon *et al.,* 2009 |
| *Lumbricus rubellus* | Heavy metals (Zn) | Survival, weight, cocoon production, metal content | No substantial differences | Lab | Spurgeon and Hopkin 1999 |
| *Lumbricus rubellus* | Heavy metals (Cd, Cu, Pb, Zn) | Survival, weight, maturation time, accumulation and excretion of Zn | Mortality of smelter worms was higher than reference strains | Lab | Spurgeon and Hopkin 2000 |
| *Lumbricus rubellus* | Heavy metals (Pb, Zn) | Fingerprinting amplified messenger RNA (mRNA) | DNA fragments specific to the induction of metal-chelating gene products | Lab and field* | Stürzenbaun *et al.,* 1998 |
| *Lumbricus rubellus* | Heavy metals (Cd, Pb, Zn) | Expression profile of TCTP (translationally controlled tumour protein) | Expression of TCTP 14 times higher than control | Lab | Stürzenbaun *et al.,* 1998 |
| *Tubifex tubifex* | Metals (Hg) | Survival, crosses | Worms raised in Hg had a higher LC50 even after 3 generations | Lab | Vidal and Horne 2003 |
| **Arthropoda** |  |  |  |  |  |
| *Agelena labyrinthica* | Heavy metals (Cd, Cu, Pb, Zn) | Size, weight, analysis of detoxifying enzymes, metal content | CarE activity was higher in pop from most contaminated site | Lab and field* | Wilczek *et al.,* 2003 |
| *Amphibalanus variegatus* | Heavy metals (Cu) | Toxicity test (immobilization), metal content, molecular markers (8 AFLP) | Higher tolerance in pop from contaminated sites but no evidence of selection | Lab and field* | Gall *et al.,* 2013 |
| *Anopheles gambiae* | Heavy metals (Cd, Cu, Pb) | Survival, selection exp, life-history traits (eggs viability, larval and pupal survival, adult emergence, fecundity, net reproductive rate | Lower magnitude of egg viability, larval and pupal survivorship, adult emergence, fecundity and net reproductive rate than the control strain | Lab | Mireji *et al.,* 2010 |
| *Attheyella crassa* | Heavy metals (Cu, Hg, Pb, Zn), hydrocarbons, antifouling paint | Molecular markers (AFLP), pop size estimates, RNA content analysis, cephalothorax length | Significant decrease in genetic diversity in the treatment, decrease in total abundance but one recovery in one treatment | Lab | Gardeström *et al.,* 2008 |
| *Balanus glandula* | Heavy metals, pesticides, PAHs | Molecular markers (6 RAPD) | Reduced genetic diversity in impacted sites | Lab and field* | Ma *et al.,* 2000 |
| *Bathycletopsyllus sp.* | Oil-drilling site | Molecular markers (COI) | Genetic diversity was in the range seen for species both from contaminated and uncontaminated sites | Lab and field* | Gregg *et al.,* 2010 |
| *Ceriodaphnia pulchella* | Heavy metals (acid effluent with many metals like Al, Fe, Cu, Zn, etc.) | Survival, life-history traits (# of released neonates) | Strong genetically-determined increase in resistance | Lab | Lopes *et al.,* 2005 |
| *Chironomus februarius* | Heavy metals (Cr, Cu, Ni, Pb, Zn), TPH | Emergence of different species, # individual each species, % total abundance, life-history traits (fecundity) | More flies emerged from the reference site and the reverse pattern occurred at the polluted site | Microcosms in the field | Bahrndorff *et al.,* 2006 |
| *Chironomus riparius* | Heavy metals (Cd) | Effect of crossbreeding, larval survival and growth rate, length of larvaes | High control mortality, lower larval growth in clean conditions, increased EC50 | Lab | Groenendijk et al., 2002 |
| *Chironomus riparius* | Model pollutant tributyltin (TBT) | Larval mortality, mean emergence time, produced egg masses/female, hatchability of egg masses, pop growth rate, molecular markers (5 microsats) | TBT-exposed strains showed increased larval mortality, slightly reduced reproductive output, and delayed larval development. Reduced genetic diversity in treatments | Lab | Nowak *et al.,* 2009 |
| *Chironomus riparius* | Heavy metals (Cd) | Survival, life-history traits (#eggs/female, tot # fertile eggs/initial 3 larvae), emergence time, molecular markers (5 microsats) | Genetic variation inversely proportional to tolerance, directly proportional to fitness in Cd conditions | Lab | Nowak *et al.,* 2008 |
| *Chironomus riparius* | Heavy metals (Cd) | Metal content, Cd excretion, weight | Increased elimination rate | Lab | Postma *et al.,* 1996 |
| *Chironomus riparius* | Heavy metals (Cd) | Survival, # of emerged males and females. Total # males and females, mortality, dry weight, metal content, # of eggs, pop growth rate | High control mortality, increased larval developmental time but no differences in larval mortality | Lab | Postma *et al.,* 1995 |
| *Chironomus riparius* | Heavy metals (Cd, Fe, Zn) | Larval survival, growth rate, body length | Slower growth of larvae from contaminated sites in control conditions, different growth responses except for Zn | Lab | Postma *et al.,* 1995 |
| *Chironomus riparius* | Heavy metals (Cd) | Survival, metal content, life-history traits (mean larval development, # males and females midges in the cage, # deposited egg-ropes, body growth of 1st instar larvae), pop growth rate, emergence time | High mortality in only one generation when NOEC values exceeded | Lab | Postma and Davids 1995 |
| *Chironomus riparius* | Heavy metals (Cu) | Survival, molecular markers (4 microsats) | Twice LC50 value in pop from contaminated sites. Polluted sites in panmixis | Lab and field* | Soeter *et al.,* 2010 |
| *Chironomus riparius* | Model pollutant tributyltin (TBT) | Emergence time, dry weight, sex ratio, life-history traits (#eggs/female, #eggs/eggs mass), hatchability, survival, molecular markers (5 microsats) | Larvae with significant tolerance, reproductive output increased in later generation. Non-random alteration in allele distribution | Lab | Vogt *et al.,* 2007 |
| *Daphnia longispina* | Heavy metals (Cu, Zn), acid mine | Survival, feeding rate | Higher survival but no other differences between populations from contaminated and reference sites | Lab | Agra *et al.,* 2010 |
| *Daphnia longispina* | Heavy metals (Cu), acid mine drainage | Survival, body length, life-history traits (# neonates/female) | Persistence tolerance (acclimated & not acclimated) genetically determined responses converge from lethal to sublethal toxicant exposures | Lab | Lopes *et al.,* 2006 |
| *Daphnia longispina* | Heavy metals (Cu), acid mine drainage | Survival, feeding inhibition, life-history traits (time to 1st brood, # neonates/brood, time inter-broods, body length females after 4th brood) | Disappearance of the most sensitive lineages, no directional change in life-cycle traits | Lab | Lopes *et al.,* 2004 |
| *Daphnia longispina* | Acid mine drainage | Survival, molecular markers (AFLP, contaminant indicative bands) | Significant correlation between individual genetic distance and tolerance | Lab and field* | Martins *et al.,* 2009 |
| *Daphnia longispina* | Heavy metals (Cu), acid mine drainage | Survival, molecular markers (20 allozymes) | Allozymes not associated with increased resistance | Lab | Martins *et al.,* 2007 |
| *Daphnia longispina* | Acid mine drainage (AMD) | Molecular markers (8 microsats) | Generally low diversity but higher diversity in the impacted population | Lab and field* | Silva *et al.,* 2010 |
| *Daphnia magna* | Pesticides | Survival, molecular markers (allozymes) | Differences in susceptibilities and correlation between tolerance levels and land use intensity | Lab and field* | Coors *et al.,* 2009 |
| *Daphnia magna* | Heavy metals (Cd) | Survival, hsp70 expression, Cd accumulation | Highest EC50 in the clone displaying the lowest hsp70 expression | Lab | Haap and Köhler 2009 |
| *Daphnia magna* | Heavy metals (Cd) | EC10, reproductive performance, heritability | Very variable heritability among different clones | Lab | Messiaen *et al.*, 2013 |
| *Daphnia magna* | Heavy metals (Cd) + temperature | Net reproductive rate, animal model (broad and narrow sense heritability, additive genetic variance) | Significant heritability of net reproductive rate under sub-lethal concentration | Lab | Messiaen *et al.,* 2012 |
| *Daphnia magna* | Heavy metals (Cd) | Survival, life-history traits (time to 1st brood, length of parents at first brood, # of offspring at 1st brood, length of parents at day 21 and total reproduction). Pop growth rate estimates | Genetic correlation between traits was affected by Cd | Lab | Messiaen *et al.,* 2010 |
| *Daphnia magna* | Heavy metals (Cd) | Survival, selection exp, life-history traits (day first culture brood, # of young produced in each culture), estimates of mean life span, mean # young/female, intrinsic rate of increase), molecular markers (AFLP). Cu, Pb, phenol tolerance test after selection exp | After selection exp: increase in Cd resistance in few generations but reduced size, lower genetic variability and sensitivity to another toxicant | Lab | Ward and Robinson 2005 |
| *Daphnia pulex* | Heavy metals (Cd) | Microarray, body length, lipid-ovary index, # of clutches, per capita birth rate | Identified genes were associated with Cd-induced phenotypes and pop-level outcomes. Three genes coding for metallothionein | Lab | Shaw *et al.* 2007 |
| *Drosophila melanogaster* | Heavy metals (Cd) | Survival, selection exp, life-history traits (developmental time, fecundity, emergence weight) crosses and backcrosses | Increased fitness in polluted environment. Crosses: evolved resistance due to a single sex-linked gene | Lab | Shirley and Sibly 1999 |
| *Drosophila subobscura* | Heavy metals (Pb) | Fecundity, egg-to-adult viability and developmental time | Higher increase in variation in fecundity and developmental time in pop from uncontaminated site | Lab | Kenig *et al.,* 2014 |
| *Drosophila subobscura* | Heavy metals (Pb) | Fecundity, developmental time, egg-to-adult viability | Higher fecundity, viability, faster egg-to-adult development in pop from most polluted site | Lab | Kenig *et al.,* 2013 |
| *Drosophila subobscura* | Heavy metals (Pb) | Developmental stability, wings size | Genotypes reared on the highest Pb concentration were in developmental homeostasis | Lab | Kurbalija *et al.,* 2010 |
| *Folsomia candida* | Heavy metals (Cd) | EC50, Metallothionein-like gene expression | Expression was induced by Cd exposure but not by an oxidative stress | Lab | Nakamori *et al.,*2010 |
| *Folsomia candida* | Heavy metals (Cd) | Life-history traits (# of juveniles produced), transcriptome analysis | Divergent fitness responses and significant differences between the Cd-affected transcriptomes | Lab | Nota *et al.,* 2013 |
| *Folsomia candida* | Heavy metals (Ba, Cd, Co, Cr, Pb, Zn), phenanthrene | Methallothionein gene expression | Metallothionein expression induced after exposure to all metals (except Cr) | Lab | Nota *et al.,* 2011 |
| *Folsomia candida* | Heavy metals (Ba, Cd, Co, Cr, Pb, Zn) | Gene expression analysis (classifier analysis), life-history traits (# of juveniles), EC10 and EC50, filed soil effect on reproduction | 188 genes could discriminate between 6 different metals (83% of accuracy in predicting the correct classes for samples) | Lab | Nota *et al.,* 2010 |
| *Gammarus fossarum* | Heavy metals (Cd) | Survival, sib analysis | No heritability, no additive genetic components | Lab | Chaumot *et al.,* 2009 |
| *Helisoma trivolvis* | Heavy metals | Morphometric data (body size), molecular markers (5 isozymes) | Body size associated with a particular genotype. Allele selected in the contaminated habitat may be related to contaminant tolerance and body size plasticity | Lab and field* | Benton *et al.,* 1994 |
| *Hyalella azteca* | Heavy metals (Cd, Zn), acidification | Survival, molecular markers (3 allozymes) | Conflicting data, inconsistent results | Lab | Duan *et al.,* 2001 |
| *Hyalella azteca* | PAH, fluoranthene contaminants | Survival, molecular markers (3 allozymes) | Alteration of the frequencies of several genotypes | Lab | Duan *et al.,* 2000 II |
| *Hyalella azteca* | Pesticides | Molecular markers (COI and 28S), sequencing of voltage-gated sodium channel, gene expression analysis | Point mutations were responsible for differences in sensitivities | Lab | Weston *et al.,* 2013 |
| *Isonychia bicolor* | Metals (Hg) | Survival, morphometric data (body length), molecular markers (29 allozymes) | Fitness differences to Hg exposure among allozyme variants | Lab and field* | Snyder and Hendricks 1997 |
| *Isotoma notabilis* | Heavy metals (Cu, Zn) | Survival, growth rate, life-history traits (cumulated # of eggs/replicate, cumulated # of eggs/treatment and mean # of reproductive day), mean maximum length | Produced more juveniles and became more abundant than the reference population in all treatments | Lab | Tranvik *et al.,* 1993 |
| *Kiefferulus intertinctus* | Heavy metals (Cr, Cu, Ni, Pb, Zn), other TPH | Emergence of different species, # individual each species, % total abundance, life-history traits (fecundity) | Absence of significant site by treatment interaction terms for some species | Microcosms in the field | Bahrndorff *et al.,* 2006 |
| *Leander intermedius* | Heavy metals (Cd, Cu, Mn, Pb, Zn) | Survival, molecular markers (45 RAPD), metallothionein level | Reduced genetic diversity. All individuals of the species may possess mechanisms to cope with elevated concentrations of metals in their environment | Lab and field* | Ross *et al.,* 2002 |
| *Leptodiaptomus minutus* | Acidification | Survival, molecular markers (COI) | Reduced variation in pH tolerance compared to the recovery period (strong selection). No variance differences of survival between pre-industrial and acid time period | Lab and field* | Derry *et al.,* 2010 |
| *Microarthridion littorale* | Pesiticides | Survival | The most common haplotype in contaminated sites was the most tolerant in lab | Lab | Schizas *et al.,* 2001 |
| *Nectopsyche albida* | Metals (Hg) | Survival, molecular markers (6 allozymes) | Genotypes showing differential sensitivity | Lab | Benton *et al.,* 1992 |
| *Nitocra lacustris* | Xenobiotics | Adult survival, life-history traits (# of offsprings surviving), molecular markers (RFLP on mtDNA), pop size effect on diversity (on dataset) | Loss of haplotype diversity (when reproductive output decreased) was due to an increase in the most common haplotype | Lab | Street *et al.,* 1998 |
| *Onychiurus armatus* | Heavy metals (Cu, Zn) | Survival, growth rate, reproduction (cumulated # of eggs/replicate, cumulated # of eggs/treatment and mean # of reproductive day), mean maximum length | Exposed population reached reproductive size faster and laid more eggs than the reference population at all treatment | Lab | Tranvik *et al.*, 1993 |
| *Orchesella bifasciata* | Heavy metals (Cu, Zn) | Survival, grazing activity, biomarker (70 kDa stress proteins) | High variability even within replicates | Lab | Köhler *et al.,* 1999 |
| *Orchesella cincta* | Heavy metals (Cd) | Molecular markers (AFLP, microsats, functional MT promoter) | No reduced genetic diversity in impacted sites in neither markers | Lab | Costa *et al.,* 2012 |
| *Orchesella cincta* | Heavy metals (Cd, Zn, Pb, Cu) | Molecular markers (22 allozymes) | No decreased variation, frequency of Got alleles correlated with metal tolerance | Lab and field* | Frati *et al.,* 1992 |
| *Orchesella cincta* | Heavy metals (Cd, Cu, Fe, Ni, Pb, Zn) | Metallothionein alleles | One allele was higher in pop from polluted sites. Association between allele frequencies and specific metals | Lab and field* | Janssens *et al.,* 2008 |
| *Orchesella cincta* | Heavy metals (Cd) | Methallotionein coding region sequencing, oxidative stress inducer, moulting hormone analysis | Deviation from neutral expectation in tolerant pop, promoter allele frequencies differed significantly from reference | Lab and field* | Janssens *et al.,* 2007 |
| *Orchesella cincta* | Heavy metals (Cd) | Excretion efficiency, metal content, gut pellets analysis, body growth rate | Milder growth reduction upon exposure, probably caused by decreased body concentrations of Cd in pop from contaminated sites | Lab | Posthuma *et al.,* 1992 |
| *Orchesella cincta* | Heavy metals (Cd) | Survival, excretion efficiency, offspring-parent regression, half-sib analysis | Offspring-parent regressions showed that additive genetic variation for Cd excretion efficiency was present in the population from the reference site | Lab | Posthuma *et al.,* 1993 |
| *Orchesella cincta* | Heavy metals (Cd) | Survival, life-history traits (body growth, age and weight at 1st reproduction, clutch size) | High control mortality in pop from contaminated site, lower age at 1st reproduction | Lab | Posthuma *et al.*, 1993 |
| *Orchesella cincta* | Heavy metals (Cd) | Transcriptomics: cDNAs gene expression | Reference population showed a strong signature of stress-induced genome-wide perturbation of gene expression while tolerant ones maintained normal gene expression | Lab | Roelofs *et al.,* 2009 |
| *Orchesella cincta* | Heavy metals (Cd) | Metallothionein expression and gene expression pattern | Tolerant animals maintained normal gene expression while reference animals showed stress induced gene expression | Lab | Roelofs *et al.,* 2007 |
| *Orchesella cincta* | Heavy metals (Cd) | Molecular markers (RFLP), mt gene expression (real time RT-PCR) on parents and offspring RNA and subjected to regression analysis | Significant heritability, 8 promoter alleles showed structural variation, 3 alleles showed increased frequencies in families with high mt expression. Another gene involved in stress response | Lab | Roelofs *et al.,* 2006 |
| *Orchesella cincta* | Heavy metals (Cd) | *Mt* mRNA | *Mt* expression levels higher in pop originating in a polluted site compared to reference pop | Lab | Sterenborg and Roelofs 2003 |
| *Orchesella cincta* | Heavy metals | Metallothionein gene, molecular markers (SSCP, RFLP) | Selection on metallothionein gene. Analysis of molecular variance assigned a small, but significant amount of the total variance to differences between metal-stressed and non-stressed populations | Lab and field* | Timmermans *et al.,* 2007 |
| *Orchesella cincta* | Heavy metals (Cd) | Survival, *mt* mRNA expression (RT-PCR) | Five out of eight pop evolved increased Cd tolerance, MT mRNA expression of populations from polluted sites was higher than reference sites but not correlation with [Cd] in the field and with Survival rates (other mechanisms must be involved in prolonged tolerance) | Lab | Timmermans *et al.,* 2005 |
| *Orchesella cincta* | Heavy metals (Cd) | Metallothionein locus genotyping (RFLP–PCR) and metallothionein induction | higher expression of metallothionein gene in pop from contaminated site and large degree of polymorphism of its promoter | Lab and field* | Van Straalen *et al.,* 2011 |
| *Pardosa lugubris* | Heavy metals (Cd, Cu, Pb, Zn) | Size, weight, analysis of detoxifying enzymes, metal content | CarE activity was higher in pop from most contaminated site. Better adaptation than *Agelena labyrinthica* | Lab and field* | Wilczek *et al.,* 2003 |
| *Pardrosa saltans* | Heavy metals (Cd, Pb, Zn) | Metal content, metallothionein level | MT concentration did not increase in exposed pop. Adult size and conditions correlated negatively and egg mass positively with [Cd] | Lab | Eraly *et al.,* 2011 |
| *Pardrosa saltans* | Heavy metals (Cd) | Survival, individual growth rate, metallothionein level, metal content | Increased protein level in both treatments and control but no significant correlation with [Cd] | Lab | Eraly *et al.*, 2010 |
| *Peramphithoe parmerong* | Heavy metals (Cu) | Survival, broad sense heritability of survival and growth, body size, feeding rate, full-sib, split family design | Significant genotype-by-environment interaction in offspring survival between treatments and controls revealed variation in tolerance. Smaller size and lower feeding rate in treatments | Lab | Pease *et al.,* 2010 |
| *Pirata piraticus* | Heavy metals (Cd) | Life-history traits (initial weight, growth rate and egg size), crosses, animal model | Reduced growth rate and increased egg size in the contaminated site, low heritability | Lab | Hendrickx *et al.,* 2008 |
| *Pirata piraticus* | Heavy metals (Cu, Cd, Zn) | Life-history traits (reproductive output, fecundity, egg size) | Reduced reproductive output and fecundity, increased egg size in pop from contaminated sites | Lab | Hendrickx *et al.,* 2003 |
| *Platynympha longicaudata* | Heavy metals (Zn, Pb, Cd, Cu, Mn) | Survival, molecular markers (45 RAPD), metallothionein level | Reduced gen diversity. Lower survival in controls. Clear genetic diversity differences | Lab and field* | Ross *et al.,* 2002 |
| *Porcellio scaber* | Heavy metals (Zn) | Feeding rate, food and Zn assimilation, metal content | Higher growth efficiency, lower increase in Zn body burden | Lab | Donker *et al.,* 1996 |
| *Porcellio scaber* | Heavy metals (Cd, Cu, Fe, Zn) | Life-history traits (body growth, sex ratio, reproduction at 1st and 2nd generations), metal content | Earlier reproduction, increased reproduction allocation, lower weight | Lab | Donker *et al.,* 1993 |
| *Spodoptera exigua* | Heavy metals (Cd, Zn) | Survival rate, metal content, Catalase, Superoxidase dismutase and glutathione transferase activity | Pre-exposure control and Zn- pre-exposed organisms had lower survival than control animals. Metal content increased with concentration and Cd-pre-exposure had a significant effect in metal accumulation in larvae | Lab | Kafel *et al.,* 2014 |
| *Spodoptera exigua* | Heavy metals (Cd) | Glutathione, protein thiols, total anti-oxidant capacity level, glutathione transferase activity. Metal content. Larval survival, larval duration time and last instar body weight | Higher metal content, higher mortality and longer duration of the larval stage in one-generation exposed insects in comparison with those exposed for many generations. Positive relation between higher metal content and glutathione oxidation | Lab | Kafel *et al.,* 2012 |
| *Tetrix tenuicornis* | Heavy metals | Molecular markers (20 RAPD), metal content | Reduced genetic diversity in pop from contaminated sites. Significant changes in elemental concentrations | Lab and field* | Grzywacz *et al.,*2012 |
| *Thamnocephalusplatyurus* | pesticides | Survival | Higher survival in populations from contaminated sites | Lab | Brausch and Smith 2009 |
| *Tigriopus angulatus* | Heavy metals (Cu) | Growth rate, juvenile survival, life-history traits (age specific survival, intrinsic rate of natural increase) | Juvenile survival affected but unaffected intrinsic rate of natural increase | Lab | Medina *et al.,* 2009 |
| **Bryozoa** |  |  |  |  |  |
| *Bugula neritina* | Heavy metals (Cu) | Larval attachment success, post-metamorphic survival and growth of recruits. Field: post-exposure survival, growth of colonies | Resistance to Cu closely related to the relative levels of pollution experienced by the source populations | Lab and field | Piola and Johnston 2006 |
| *Celleporella hyalina* | Acidification | Life-history traits (estimates of specific growth rate, growth efficiency, colony conditions, reproductive investment, gender allocation), SEM analysis | Significant effect of different clone on growth rate, reproductive investment and sex ratio, with clones showing contrasting responses to the various temperature and pH combinations | Lab | Pistevos *et al.,* 2011 |
| *Watersipora subtorquata* | Heavy metals (Cu) | Survival, larval settlement and metamorphosis, full-sib split family design, larval size | No difference in tolerance between sites. Larval size significantly different | Lab | McKenzie *et al.,* 2011 |
| **Chordata** |  |  |  |  |  |
| *Styela plicata* | Heavy metals (Cu) | Hatching success, quantitative genetic breeding design | Difference in genetic basis of resistance between high and low concentration | Lab | Galletly *et al.,* 2007 |
| **Cnidaria** |  |  |  |  |  |
| *Nematostella vectensis* | Heavy metals (Cd, Cu, Hg, Zn) | Transcriptomics (RNA-seq) | Hg greatest impact followed by Cu, Zn and Cd. Co-up-regulation of immediate-early transcription factors such as Egr1, AP1 and NF-jB | Lab | Elran *et al.,* 2014 |
| **Echinodermata** |  |  |  |  |  |
| *Centrostephanus rodgersii* | Acidification | Crosses, life-history traits (fertilization success, cleavage success, sire success, normal gastrulae) | Presence of tolerant genotypes, early development not constrained in adapting | Lab | Foo *et al.,* 2012 |
| *Strongylocentrotus franciscanus* | Acidification | Full-factorial breeding design, larvae size, heritability quantification | Greater levels of phenotypic and genetic variation for larval size in future CO_2_ conditions, greater differential in mean trait values before and after selection |  | Sunday *et al.,* 2011 |
| *Strongylocentrotus purpuratus* | Acidification | Growth rate, estimates of additive genetic variance for larval size with breeding exp | Abundant genetic variation for body size under elevated pCO_2_ | Lab | Kelly *et al.,* 2013 |
| *Strongylocentrotus purpuratus* | Acidification | Molecular markers (SNPs), genomics | Allelic change in 40 functional classes of proteins involving hundreds of loci | Lab | Pespeni *et al.,* 2013 |
| **Mollusca** |  |  |  |  |  |
| *Biomphalaria glabrata* | Heavy metals (Cd) | Shell length, time-to-death, hatching success, # eggs/snail/day, time to maturity, eggs per mass | Higher tolerance to chronic and lethal [Cd] in resistant strain | Lab | Salice *et al.,* 2010 |
| *Cantareus aspersus* | Heavy metals (Cd, Pb, Zn) | Metal content, reciprocal transplant | Heavier shell than *C. Nemoralis*, no significant differences in metal accumulation | Field | Fritsch *et al.,* 2011 |
| *Cassostrea gigas* | Heavy metals (Cd, Cu, Zn), pesticides | Metal content, gene expression | Genetic differentiation in the pop from contaminated site, two specific alleles were associated with metal sensitivity | Lab and field* | David *et al.,* 2012 |
| *Cassostrea gigas* | Mixed chemicals | Microarray | Expression increased with pollution level. Potential selective effect on heterozygote frequency | Lab and field* | David *et al.,* 2007 |
| *Cassostrea gigas* | Tribultyltin (TBT) | Survival, molecular markers (6 allozymes) | Allele frequencies varied significantly between resistant and sensitive pop | Lab | Tanguy *et al.,* 1999 |
| *Cassostrea angulata* | Heavy metals (Cu, Zn) | Enzymatic activities (antioxidant defences, oxidative damage), metal content | More sensitive to pollution than *Oyster,* absent in the most contaminated site | Lab | Funes *et al.,* 2006 |
| *Cepaea nemoralis* | Heavy metals (Cd, Pb, Zn) | Metal content, reciprocal transplant | Greater internal metal concentration than *C. Aspersus,* no significant differences in metal accumulation | Field | Fritsch *et al.,* 2011 |
| *Dreissena polymorpha* | Heavy metals | Molecular markers (COI and 1 microsat), transcriptome analysis | Expression level correlated with variation in fitness and loads of heavy metals | Lab and field* | Navarro *et al.,* 2013 |
| *Lymanea stagnalis* | Pesticides | Life-history traits (individual growth, female reproduction, hatching success), molecular markers (12 microsats) | Pesticide and other human pressures had little correspondence with evolutionary patterns | Lab and field* | Bouétard *et al.,* 2014 |
| *Macoma balthica* | Heavy metals (Cu) | Survival, molecular markers (7 isozymes) | Differences in sensitivity and genetic diversity between pop sampled from the distribution limits of the species | Lab | Hummel *et al.,* 1997 |
| *Macoma balthica* | Heavy metals (Ag, As, Cd, Cu, Mn, Pb, Se, V and Zn) | Metal content, molecular markers (DALP) | Individual with irregular shell shape exhibited higher concentrations of all metals | Lab and field* | Sokolowski *et al.,* 2002 |
| *Mytilus edulis* | Heavy metals (Cu) | Survival, embryo development, crosses | Development less affected in pop from contaminated site, maternal effect revealed by crosses | Lab | Hoare *et al.,* 1995 |
| *Mytilus galloprovincialis* | Heavy metals (Cu, Zn) | Enzymatic activities (antioxidant defences, oxidative damage), metal content | Combined increase of antioxidant defences and metal stabilization by complexation protect them | Lab | Funes *et al.,* 2006 |
| *Mytilus galloprovincialis* | Heavy metals, pesticides, PAHs | Molecular markers (6 RAPD) | Same haplotype shared in individuals at impacted sites | Lab and field* | Ma *et al.,* 2000 |
| *Mytilus galloprovincialis* | Municipal, industrial and shipyard wastewaters | Comet assay, micronucleus test, oxidative stress parameters, molecular markers (8 microsats) | Higher levels of genetic diversity in the pop from contaminated site | Lab and field* | Štambuk *et al.,* 2013 |
| *Mytilus galloprovincialis* | Heavy metals (Cd, Cu, Hg), organic contaminants | Gene expression, shell length | Some gene markers traced organic contaminants more than heavy metals | Lab | Venier *et al.,* 2006 |
| *Mytilus trossulus* | Acidification | Full-factorial breeding design, larvae size, heritability quantification | Less level of phenotypic variation for larval size in future CO_2_ conditions than *Strongylocentrotus franciscanus* | Lab | Sunday *et al.,* 2011 |
| *Nucella lapillus* | Tributyltin (TBT) | Molecular markers (18 allozymes), shell size | No differences in genetic diversity but higher variation in shell size in pop from contaminated site | Lab and field* | Plejdrup *et al.,* 2006 |
| *Perna viridis* | Heavy metals (Cd, Cu, Pb, Zn) | Metal content, shell size, molecular markers (19 microsats) | Two clusters based on location and heavy metal contamination | Lab and field* | Yap *et al.,* 2013 |
| *Perna viridis* | Heavy metals (Cd, Cu, Hg, Pb, Zn) | Metal content, molecular markers (10 allozymes), metallothionein level | Increased genetic variation and altered allozyme frequencies in the pop from contaminated site | Lab and field* | Yap *et al.,* 2004 |
| *Ruditapes decussatus* | Heavy metals | Two molecular markers, protein marker (metallothionein) | Presence of a relationship between metallothionein concentrations and the level of metal pollution. Differential metallothionein induction between species | Lab and field* | Moraga *et al.,* 2002 |
| *Ruditapes philippinarum* | Heavy metals | Two molecular markers, protein marker (metallothionein) | Relationship between metallothionein concentrations and the level of metal pollution. Differential metallothionein induction between species | Lab and field* | Moraga *et al.,* 2002 |
| *Saccostrea glomerata* | Acidification | Oxygen consumption at gravid stage, standard metabolic rate, larval percentage survival, larval life-history traits (mean shell length, stage of development) | Larvae spawned from adults exposed to elevated Pco2 were larger and developed faster, but similar survival compared with larvae spawned from adults exposed to ambient Pco2 | Lab | Parker *et al.,* 2012 |
| *Sphaerium novaezelandiae* | Heavy metals (Zn) | Survival, reburial rate, molecular markers (allozymes) | No differences in mortality. Significant difference in reburial rates | Lab | Phillips and Hickey 2010 |
| **Nematoda** |  |  |  |  |  |
| *Caenorhabditis elegans* | Uranium (U) | Survival, fecundity, early and late growth (body length), heritability | Heritability decreases for fecundity and early growth in polluted environments. Decrease in heritability not proportional to the pop fitness reduction | Lab | Dutilleul *et al.,* 2015 |
| *Caenorhabditis elegans* | Uranium (U) | Life-history traits (brood size, index of fertility, male body length and body bend frequency) | Reduced stability of trait structure and higher capacity to respond by acclimation. Lower evolutionary responses compared to salt treatment. Higher pop rate of increase | Lab | Dutilleul *et al.,* 2014 |
| *Caenorhabditis elegans* | Uranium (U) | Survival, generation time, brood size, body length, body bend | At low concentrations negative effects reduced in the 2nd and 3rd generation (acclimation) while at high negative effects increased across generation | Lab | Dutilleul *et al.,* 2013 |
| **Platyhelminthes** |  |  |  |  |  |
| *Polycelis tenuis* | Heavy metals (Cd) | Survival, EC50, LC50, body size, reproduction, metal content | One of the pop had higher LC50 and eliminated Cd at a higher rate than reference pop | Lab | Indeherberg *et al.,* 1999 |
| **FISH** |  |  |  |  |  |
| **Chordata** |  |  |  |  |  |
| *Ameiurus nebulosus* | Toxic chemicals (PAHs) | Molecular markers (RFLP on mtDNA) | Reduced genetic diversity in impacted sites | Lab and field* | Murdoch and Hebert 1994 |
| *Anguilla anguilla* | Heavy metals (As, Cd, Cr, Cu, Hg, Ni, Pb, Zn and Se) | Metal content, molecular markers (12 allozymes and 8 microsats) | Negative correlation between pollution load and fitness. Reduced genetic variability in strongly polluted eels | Lab and field* | Maes *et al.,* 2005 |
| *Anguilla anguilla* | PCBs | Transcriptomic platform for global gene expression | High expression of detoxification genes, lowered expression of genes involved in metabolism | Lab and field* | Pujolar *et al.,* 2012 |
| *Campostoma anomalum* | Heavy metals, low water quality | Molecular markers (10 allozymes) | Reduced genetic diversity in impacted sites (higher) | Lab and field* | Heithaus and Laushman 1997 |
| *Catostomus occidentalis* | Pesticides | Molecular markers (AFLP and microsats) | No correlation with pesticide exposure history and genetic structure | Lab and field* | Whitehead *et al.,* 2003 |
| *Coregonus lavaretus* | Industrial pollution | Body size and weight, sex, gonad maturity, fatness, stomach fullness | Smaller body size and more variable sexual maturation time, frequency of spawning and life span decreased in treatments | Lab | Moiseenko 2002 |
| *Cyprinodon variegatus* | Heavy metals (Zn), phenantrene | Survival, heritability | Low heritability, negative relationship between heritability of resistance and # of contaminants | Lab | Klerks and Moreau 2001 |
| *Danio rerio* | Heavy metals (Cd, Cu) | Gene expression of ABCB10 gene | ABCB10 gene up-regulated as a result of metallic contamination | Lab | Sabri *et al.,*2012 |
| *Etheostoma blennioides* | Heavy metals, low water quality | Molecular markers (10 allozymes) | Reduced genetic diversity in impacted sites (intermediated) | Lab and field* | Heithaus and Laushman 1997 |
| *Etheostoma caeruleum* | Heavy metals, low water quality | Molecular markers (10 allozymes) | Reduced genetic diversity in impacted sites (lowest) | Lab and field* | Heithaus and Laushman 1997 |
| *Fundulus heteroclitus* | PAHs | Survival (embryos), developmental delays, heart rate, morphology, microarray | Significant differences in each trait except in the microarray | Lab | Bozinovic and Oklesiak 2010 |
| *Fundulus heteroclitus* | PCBs | Hatching success, larval survival, larval growth, CYp1A expression | No induction of CYp1A in fish from contaminated sites | Lab | Elskus *et al.,* 1999 |
| *Fundulus heteroclitus* | PCBs, PAH | cDNA arrays | Lack of gene expression variation (common mechanisms in different pop), 2 genes have a common response | Lab and field* | Fisher and Oklesiak 2007 |
| *Fundulus heteroclitus* | Dioxin | Molecular markers (25 SNPs) | AHR1 locus is highly polymorphic, allele frequencies differ between some dioxin-sensitive and dioxin-resistant populations. But the proteins encoded do not differ functionally | Lab and field* | Hahn *et al.,* 2004 |
| *Fundulus heteroclitus* | Organic chemicals (bleached kraft mill effluent) | Molecular markers (15 allozymes) | Increased temporal variability in the pop closest to the source of pollution | Lab and field* | Kirchhoff *et al.,* 1999 |
| *Fundulus heteroclitus* | PCBs | Molecular markers (232 AFLP) | No differences in genetic diversity | Lab and field* | McMillan *et al.,* 2006 |
| *Fundulus heteroclitus* | PAHs | Aryl hydrocarbon receptor gene expression | Lack of inducibility of genes that are normally inducible by AHR agonists in pop from contaminated site | Lab | Meyer *et al.,* 2003 |
| *Fundulus heteroclitus* | PAHs, menadione, *t*-butyl hydroperoxide | Antioxidant parameters | Upregulated antioxidant defenses | Lab | Meyer *et al.,* 2003 |
| *Fundulus heteroclitus* | Xenobiotics, PAH | Survival, developmental abnormalities, developmental rate, resistance of the larvae to phototoxicity | Increased ability of F1, F2 to develop normally and to survive than reference individuals. Fitness cost | Lab | Meyer and Di Giulio 2003 |
| *Fundulus heteroclitus* | PCBs/dioxin | Egg deposition, hatching success, and larval growth and survival | No site-related differences | Lab | Monosson *et al.,* 1995 |
| *Fundulus heteroclitus* | PAHs | Molecular markers (14 allozymes and one general protein) | Significant correlation between individual genetic distance and differences among site in [PAH] | Lab and field* | Mulvey *et al.,* 2002 |
| *Fundulus heteroclitus* | PCBs | Hatching and survival of embryos, embryonic urinary bladder fluorescence (correlated to EROD), EROD activity. LC20, EC50 | Variation in the magnitude of heritability but similarities among biochemical mechanisms across impacted pop | Lab | Nacci *et al.*, 2010 |
| *Fundulus heteroclitus* | Dioxin-like contaminants | Survival, EROD activity, changes in the activity of cytochrome P450 enzyme | Inherited resistance, lethal to reference individuals | Lab | Nacci *et al.,* 1999 |
| *Fundulus heteroclitus* | Dioxin-like contaminants | Larval survival, incorporation of PCB 126 and radioactive content | Increased tolerance in population from contaminated site | Lab | Nacci *et al.,* 2002 |
| *Fundulus heteroclitus* | PAHs, fluoranthene contaminants | Cardiovascular abnormalities in embryos | Uncontaminated pop higher abnormalities, heritable differences | Lab | Ownby *et al.,* 2002 |
| *Fundulus heteroclitus* | PCB-126 | cDNA arrays, RNA-seq to confirm results | Striking differences between pop. In one pop other genes involved, not only CYP1A | Lab | Oleksiak *et al.,* 2011 |
| *Fundulus heteroclitus* | Dioxin-like contaminants | Molecular markers (98 SNPs), 3 AHR-related loci | Strong pop genetic structure at AHR-related loci, non-neutral change at the AHR2 locus | Lab and field* | Reitzel *et al.,* 2014 |
| *Fundulus heteroclitus* | PCBs | Selection exp, survival, molecular markers (10 allozymes) | Allele frequencies reflect a pattern of isolation by distance but nothing else | Lab and field* | Roark *et al.,* 2005 |
| *Fundulus heteroclitus* | PCB 126 | Survival, developmental effects, transcriptomics | Desensitization of aryl-hydrocarbon receptor-mediated transcriptional activation, which is associated with extreme tolerance | Lab | Whitehead *et al.,* 2012 |
| *Fundulus heteroclitus* | PCBs | Transcriptomics, hatching, survival, developmental abnormalities | Dramatic effects in the sensitive pop (expression associated with toxicity), genome-wide expression was comparatively refractory to PCB induction in the tolerant pop. Global blockade of AHR signalling pathway in tolerant pop (but leaky with extreme concentrations) | Lab | Whitehead *et al.,* 2010 |
| *Fundulus heteroclitus* | Chemical pollutants | Molecular markers (300 AFLP) | 1-6% loci under selection or linked to areas of the genome in polluted pop. Shared loci among polluted sites | Lab and field* | Williams and Oleksiak 2008 |
| *Fundulus heteroclitus* | Not specified | Molecular markers (458 SNPs) | Non-neutral patterns, one SNP in the gene identified as refractory to induction | Lab and field* | Williams and Oleksiak 2011 |
| *Gambusia affinis* | Radionuclide | Molecular markers (RAPD), DNA strand breakage | Specific alleles more abundant in pop from contaminated site, higher DNA integrity | Lab and field* | Theodorakis *et al.,* 1999 |
| *Gambusia affinis* | Radionuclide | Molecular markers (RAPD) | The frequency of three markers was greater in the contaminated than the reference sites. Same pattern of band frequency shift of another species of the genus | Lab | Theodorakis *et al.,* 1998 |
| *Gambusia affinis* | Radionuclide | Molecular markers (allozymes and 40 RAPD), fecundity (brood size/body length) | Allozymes: higher % of polymorphism and heterozygosity in the pop from contaminated site RAPD: increased genetic diversity and 17 out of 142 bands occurred at higher frequency in the pop from contaminated site | Lab and field* | Theodorakis *et al.,* 1997 |
| *Gambusia holbrooki* | Heavy metals | Morphometric data (body size), molecular markers (5 isozymes) | Body size associated with a particular genotype. Genotype associated with small body size favored | Lab and field* | Benton *et al.,* 1994 |
| *Gambusia holbrooki* | Uranium (U) | Survival, molecular markers (8 allozymes) | 2nd-generation fish from polluted sites more tolerant, lower genetic variation | lab and field* | Keklak *et al.,* 1994 |
| *Gambusia holbrooki* | Heavy metals (Hg) | Size-at-age data, otoliths analysis, weight and length, # eggs and developing embryos/gravid female, sex ratio, molecular markers (8 allozymes) | Associations between genotypes (or different metabolism) and responses to stress | Lab | Mulvey *et al.,* 1995 |
| *Gambusia holbrooki* | Radionuclide | Molecular markers (RAPD) | The frequency of three markers was greater in the contaminated than the reference sites. Same pattern of band frequency shift of another species of the genus | Lab and field* | Theodorakis *et al.,* 1998 |
| *Gasterosteus aculeatus* | Pulp mill | Molecular markers (AFLP) | F_ST_-outlier analysis: non-neutral distribution in polluted sites (21 loci) | Lab and field* | Lind and Grahn 2011 |
| *Gillichthys mirabilis* | Mixed chemicals | Individual growth rate, size-distribution, reciprocal transplant | No significant differences in growth | Field | Forrester *et al.,* 2003 |
| *Gobio gobio* | Heavy metals (Cd, Zn) | Molecular markers (11 allozymes, 7 microsats) | Differences at 2 allozyme loci, 2 microsats appeared to be under selection, direct relationship between fish conditions and one allozyme allele which showed a large difference in allele frequency | Lab and field* | Knapen *et al.,* 2009 |
| *Gobio gobio* | Heavy metals (Cd, Zn) | Molecular markers (metallothionein concentrations mRNA levels) | Ratio of the long mRNA variant relative to total MT mRNA was surprisingly constant, independent of exposure history | Lab and field* | Knapen *et al.,* 2007 |
| *Gobio gobio* | Heavy metals (Cd) | Survival, Cd uptake, metallothionein analysis in liver and gill tissues | Higher survival of polluted pop, faster production and higher levels of MTLD | Lab | Knapen *et al.,* 2004 |
| *Gobionellus boleosoma* | PAHs | Survival, molecular markers (13 isozymes) | No differences between polluted and unpolluted sites | Lab and field* | Klerks *et al.,* 1997 |
| *Heterandria formosa* | Heavy metals (Cd) | Molecular markers (7 microsats) | Lower heterozygosity in selection pop | Lab | Athrey *et al.,* 2007 |
| *Heterandria formosa* | Heavy metals (Cd) | Selection exp, survival | Fast response to selection, higher survival than non-selection individuals. Heritability 0.50 | Lab | Xie and Klerks 2003 |
| *Lepomis auritus* | Heavy metals, organic chemicals, ammonia | Molecular markers (13 RAPD) | Polluted pop less genetically distant from each other than they were from each of the reference sites. Frequency of unique genotypes correlated to pollutant gradient | Lab and field* | Nadig *et al.,* 1998 |
| *Leuciscus cephalus* | PAHs, PCBs, benzene, Heavy metals | Molecular markers (28 allozymes), biochemical markers (EROD + DNA damage) | Higher frequency of one particular allele in two contaminated sites. Lower DNA damage level in the impacted pop | Lab and field* | Larno *et al.,* 2001 |
| *Microgadus tomcod* | PCBs | Ligand-binding assay, gene expression assay of AHR | Six-base deletion in AHR2 as the basis of resistance | Lab and field* | Wirgin *et al.,* 2011 |
| *Perca flavescens* | Heavy metals (Cd, Cu) | Transcriptomics, molecular markers (87 SNPs, 454 sequencing of mtDNA) | AA 204 substitution (dissimilar amminoacids around an outlier) involve it in a growth enhancement that would lead to a younger age of reproduction | Lab and field* | Bélanger-Deschênes *et al.,* 2013 |
| *Pimephales promelas* | Heavy metals (Cu) | Survival, molecular markers (5 allozymes), weight | Small size in adapted individuals in which certain alleles associated to high survivorship in Cu | Lab | Schlueter *et al.,* 1995 |
| *Salmo trutta* | Heavy metals (Cu, Zn) | Molecular markers (7 microsats) | No isolation due to metal contamination. High differentiation between two close populations | Lab and field* | Durrant *et al.,* 2011 |
| *Salmo trutta* | Heavy metals (Cd, Zn) | Metallothionein level, hematocrit, condition factors, plasma chloride, molecular markers (26 allozymes) | Negative correlation between MT content and condition factor, lower heterozigosity in population from contaminated site | Lab and field* | Olsvik *et al.,* 2001 |
| *Solea Solea* | Heavy metals, pesticides, organic chemicals | Body size, body mass, molecular markers (15 microsats and MT gene) | Two loci under directional selection, no genetic differentiation in MT gene | Lab and field* | Guinand *et al.,* 2013 |
| *Umbra limi* | Acidification | Survival, molecular markers (16 allozymes) | Stressed site: higher frequencies of one particular allozyme, most tolerant fish were significantly more genetically variable | Lab and field* | Kopp *et al.,* 1992 |
| **AMPHIBIANS** |  |  |  |  |  |
| **Chordata** |  |  |  |  |  |
| *Rana arvalis* | Acidification | Embryonic and larval fitness traits (embryonic survival, larval growth, age and size at metamorphosis) | Higher embryonic and larval acid tolerance, higher larval growth but slower larval development rate and bigger size at metamorphosis | Lab | Hangartner *et al.,* 2011 |
| *Rana arvalis* | Acidification | Survival, developmental anomalies, life-history traits (length of larvae, development rate, egg size, individual growth rate), additive genetic variance | Increased tolerance, strong maternal effect, small heritability, little additive genetic variation | Lab | Merilä *et al.,* 2004 |
| *Rana arvalis* | Acidification | Embryonic survival, life-history traits (hatchling size, age), estimated rates of divergence | Higher survival and less impaired growth performance under acid conditions. High rate of divergence | Lab | Räsänen *et al.,* 2003 |
| *Rana temporaria* | PAHs, benzo[a]pyrene (BaP) | # of micronucleated erythrocytes | No correlation between PAHs level and tolerance in the lab | Lab | Marquis *et al.,* 2009 |
| *Rana temporaria* | Acidification | Survival, body size, body shape, heritability | Low additive genetic variation independent of pH treatment | Lab | Pakkasmaa *et al.,* 2003 |
| **MICROALGAE** |  |  |  |  |  |
| **Chlorophyta** |  |  |  |  |  |
| *Chlamydomonas cf. fonticola* | Acidification | Growth rate, fluctuation analysis, resistant cell count | Large variation in the number of resistant cells observed in the set 1 experiment, in contrast to the low variation in set 2 controls. Sex was crucial | Lab | Garcia-Balboa *et al.,* 2013 |
| *Chlamydomonas reinhardtii* | Acidification | Growth rate, fluctuation analysis, resistant cell count | Large variation in the number of resistant cells observed in the set 1 experiment, in contrast to the low variation in set 2 controls. Sex was crucial | Lab | Garcia-Balboa *et al.,* 2013 |
| *Dictyosphaerium chlorelloides* | Herbicides | Dose-effect response, fluctuation analysis, pop growth rate | Recurrent mutation for resistance but detrimental in terms of fitness in the absence of herbicides | Lab | Costas *et al.,* 2001 |
| *Dictyosphaerium chlorelloides* | Acidification | Growth rate, fluctuation analysis, resistant cell count | Large variation in the # of resistant cells observed in the set 1 experiment, in contrast to the low variation in set 2 controls. Resistant mutant isolated retained resistance through generations | Lab | Garcia-Balboa *et al.,* 2013 |
| *Dictyosphaerium chlorelloides* | Formaldehyde | Growth rate, photosynthetic performance, fluctuation analysis | After 50-d in inhibiting concentration rare formaldehyde-resistant cells occurred. Estimates of the frequency of formaldehyde-resistant alleles in non-extreme environment, (selection-mutation balance) | Lab | Lopez-Rodas *et al.,* 2008 |
| *Dictyosphaerium chlorelloides* | Herbicides | Growth rate, demography, fluctuation analysis | High fluctuation in # of herbicide-resistant cells observed in set 1 cultures, in contrast with low fluctuation of set 2 controls | Lab | Marv *et al.,* 2010 |
| *Scenedesmus intermedius* | Heavy metal (mixture) | Growth rate, fluctuation analysis | Resistant cells driven to extinction in the absence of metals | Lab | Baos *et al.,* 2002 |
| *Scenedesmus intermedius* | Herbicides | Growth rate, demography, fluctuation analysis | High fluctuation in # of herbicide-resistant cells observed in set 1 cultures, in contrast with low fluctuation of set 2 controls | Lab | Marvá *et al.,* 2010 |
| **Cyanobacteria** |  |  |  |  |  |
| *Microcystis aeruginosa* | Heavy metals (Cu) | Fluctuation analysis, resistant cell count, morphometric data (cell size) | After 4-w in Cu: rare Cu-resistant cells recovered. Diminished fitness in the absence of copper sulphate but small size | Lab | Garcia-Villada 2004 |
| *Microcystis aeruginosa* | Heavy metals (Cu, Ni, Zn) | Growth rate, chlorophyll *a*, total carotenoid, phycobiliprotein concentration, cell permeability, toxin concentration, morphological changes | Retention of cell viability, increased toxin concentration | Lab | Polyak *et al.,* 2013 |
| **Dinophyta** |  |  |  |  |  |
| *Alexandrium minutum* | Acidification | Selection exp, growth rate, toxin cell quota | Toxin cell quota pattern attributable to neutral mutations (final variances were significantly higher than those measured at the start of the exp) | Lab | Flores-Moya *et al.,* 2012 |
| **Haptophyta** |  |  |  |  |  |
| *Emiliania huxleyi* | Acidification | Molecular markers (minimum 1 microsat), selection exp, growth rate, cell diameter, PIC, POC/cell (and production rate) | Higher growth rates, in both the single- and multiclone exp, calcification partly restored | Lab | Lohbeck *et al.,* 2012 |
| *Emiliania huxleyi* | Acidification | Selection exp, growth rate, morphometric data (size), calcite and biomass production | Growth rates were up to 16% higher in populations adapted for 1 year to warming when assayed at their upper thermal tolerance limit. Particulate inorganic (PIC) and organic (POC) carbon production was restored to values under present-day ocean conditions (higher than control) | lab | Schlüter *et al.,* 2014 |
| *Gephyrocapsa oceanica* | Acidification | Selection exp, photosynthetic carbon fixation, growth rate, cell size, POC, PON production, C:N ratio | Enhanced growth rate and assimilations of C and N but decreased C:N ratios | Lab | Jin *et al.,* 2013 |
| **Ochrophyta** |  |  |  |  |  |
| *Gomphonema parvulum* | Heavy metals (Cu, Zn) | Algal pigment concentration, photon yeld, metal content, dry weight, % of viable cells, EC50 | EC50 higher in the strain from the polluted site, persistence tolerance to Zn after 2 y | Lab | Ivorra *et al.,* 2002 |
| **MACROALGAE** |  |  |  |  |  |
| **Hetokonta** |  |  |  |  |  |
| *Ectocarpus siliculosus* | Heavy metals (Cu) | Max quantum yield of PSII, loss of chlorophyll autofluorescence, percentage of fluorescent cells, proteomics | Differential soluble proteome profiling: identification of the induction of proteins related to processes such as energy production, glutathione metabolism as well as accumulation of HSPs. Striking expression of a stabilizing protein and a binding protein | Lab | Ritter *et al.,* 2010 |
| *Fucus serratus* | Heavy metals (Cu) | Adult and embryo growth, accumulation of Cu^2+^ by adults | Metal exclusion mechanisms involved, the Cu^2+^ resistance of the photosynthetic apparatus may also be a significant factor | Lab | Nielsen *et al.,* 2003 |

| **Table S2.** Synopsis of studies testing for pollution-driven phenotypic responses, the presence of suitable genetic variation and responses to selection. Also indicated is whether the pollutant studied has been established as the casual factor of the observed selection. The numbers in parentheses refer to methods listed in Table 1. For the source population, "C vs. R" = population from contaminated sites compared to population from reference sites, "N" = population from the wild that do not have a known history of contamination, "Lab pop" = populations grown in the lab for an undetermined amount of time. "Y" (yes) = evidence provided, "N" (no) = evidence not provided, "NA" (not applicable) = not investigated. In some cases, when the results were not easy to interpret, words like "likely" or "contrasting" are also present. Experimental selection (27) is considered both “Response to selection” and “Selection due to pollution” given that, under laboratory conditions, the selection is highly controlled and very likely due to the pollutant used. Data in bold is obtained by the combination of articles focusing on the same population (geographical coordinates are given under Source population). | | | | | | | |
| --- | --- | --- | --- | --- | --- | --- | --- |
|  |  |  |  |  |  |  |  |
| **Species** | **Source population** | **Phenotypic response** | **Presence of genetic variation** | **Response to selection** | **Selection due to pollution** | **Pop fitness measurements** | **Reference** |
| **PLANTS** |  |  |  |  |  |  |  |
| **Angiosperms** |  |  |  |  |  |  |  |
| *Acer pseudoplatanus* | C vs. R | Y (2) | NA | Y (27) | Y (27) | NA | Turner and Dickinson 1993 |
| *Acer rubrum* | C vs. R | Y (3, 5) | NA | NA | NA | NA | Kirkey *et al.,* 2012 |
| *Arabidopsis arenosa* | C vs. R | Y (5) | NA | NA | NA | NA | Przedpelska and Wierzbicka 2007 |
| *Arabidopsis sp.* | C vs. R | Y (2) | Y (16) | NA | NA | NA | Kovalchuk *et al.,* 2004 |
| *Arabidopsis halleri* | Lab pop | Y (bps) | Y (17) | NA | NA | NA | Becher *et al.,* 2004 |
| *Arabidopsis halleri* | C vs. R (*A.lyrata petraea*) | Y (bps) | Y partially (10, 11) | NA | NA | NA | Frérot *et al.,* 2010 |
| *Arabidopsis halleri* | C vs. R | Y (bps) | Y (18) | Y (24) | NA | NA | Hanikenne *et al.,* 2013 |
| *Arabidopsis halleri* | Lab pop | Y (bps) | Y (16) | NA | NA | NA | Hanikenne *et al.,* 2008 |
| *Arabidopsis halleri* | C vs. R | Y (bps) | NA | Y (23, 26, 27) | Y (27) | NA | Meyer *et al.,* 2009 |
| *Arabidopsis halleri* | C vs. R | Y (bps) | NA | N (23) | NA | NA | Pauwels *et al.,* 2005 |
| *Arabidopsis halleri* | C vs c | Y (bps) | NA | N (23) | NA | NA | Van Rossum *et al.,* 2004 |
| *Arabidopsis halleri* | C & R | Y (1) | Y (8) | Y (27) | Y (27) | NA | Willems *et al.,* 2007 |
| ***Arabidopsis halleri*** | **51.92 10.26 (Europe)** | **Y (bps, 1)** | **Y (8,10,11,16, 17, 18)** | **Y (23, 24, 26, 27)** | **Y (27)** | **NA** | **8 studies** |
| *Arabidopsis thaliana* | Lab pop | Y (bps) | Y (17) | NA | NA | NA | Becher *et al.,* 2004 |
| *Arabidopsis thaliana* | Lab pop | Y (2, 3) | Y (18) | NA | NA | NA | Marmiroli *et al.,* 2009 |
| *Betula papyrifera* | C vs. R | Y (3, 5) | NA | NA | NA | NA | Kirkey *et al.,* 2012 |
| *Betula pubescens* | C vs. R | Y (1, 3, 5) | NA | Y (27) | Y (27) | NA | Eranen 2008 |
| *Biscutella laevigata* | C vs. R | N (3) | NA | N (23) | NA | NA | Wasowicz *et al.,* 2014 |
| *Biscutella laevigata* | C vs. R | Y (5) | NA | NA | NA | NA | Wierzbicka and Panufnik 2004 |
| *Calamagrostis epigejos* | C vs. R | N (2) | NA | NA | NA | NA | Lehmann and Rebele 2004 |
| *Cynodon dactylon* | C vs. R | Y (2, 3) | Y (9) | Y (23, 27) | Y (27) | NA | Xie *et al.,* 2014 |
| *Dianthus carthusianorum* | C vs. R | Y (3, 5) | NA | Y (23) | NA | NA | Wójcik *et al.,* 2013 |
| *Dianthus carthusianorum* | C vs. R | Y (5) | NA | NA | NA | NA | Zalęcka and Wierzbicka 2002 |
| *Elodea nuttallii* | N | NA | Y (17) | NA | NA | NA | Regier *et al.,* 2013 |
| *Elsholtzia haichowensis* | C vs. R | Y (3) | NA | NA | NA | NA | Liu and Xiong 2005 |
| *Elymus repens* | C vs. R | N (2) | NA | NA | NA | NA | Lehmann and Rebele 2004 |
| *Eucalyptus calophylla* | C vs. R | N (1) | NA | N (23) | NA | NA | Egerton-Warburton 1995 |
| *Eucalyptus patens* | C vs. R | N (1) | NA | N (23) | NA | NA | Egerton-Warburton 1995 |
| *Eucalyptus rudis* | C vs. R | N (1) | NA | N (23) | NA | NA | Egerton-Warburton 1995 |
| *Hordeum vulgare* | Lab pop | Y (3) | NA | NA | NA | NA | Patra and Panda 1998 |
| *Mimulus guttatus* | C vs. R | Y (1, 5) | Y (10) | Y (27) | Not clear (27, 28) | NA | Macnair *et al.,* 1993 |
| *Mimulus luteus* | C vs. R | Y (5) | NA | NA | NA | NA | Ginocchio *et al.,* 2002 |
| ***Mimulus guttatus*** | **37.94 -120.69 (USA)** | **Y (1, 5)** | **Y (10)** | **Y (27)** | **Y (27, ~28, 29)** | **NA** | **2 studies** |
| *Plantago arenaria* | C vs. R + other species | Y for Cu (2, 3) | NA | NA | NA | NA | Remon *et al.,* 2007 |
| *Poa annua* | C vs. R | NA | NA | Y (23, 27) | Y (27) | NA | Chen *et al.,* 2003 |
| *Prosopis sp.* | C vs. R and vendor seeds | Y (1, 3, 5) | NA | NA | NA | NA | Haque *et al.,* 2009 |
| *Sedum alfredii* | C vs. R | Y (3) | NA | Y (23) | NA | NA | Deng *et al.,* 2007 |
| *Silene dioica* | C vs. R | Y (3) | NA | NA | NA | NA | Kováčik *et al.,* 2010 |
| *Silene paradoxa* | C vs. R | NA | NA | Y (23) | NA | NA | Mengoni *et al.,* 2001 |
| *Silene paradoxa* | C vs. R | NA | NA | Y (23) | Y (29) | NA | Mengoni *et al.,* 2000 |
| ***Silene paradoxa*** | **43.35 9.91 (Europe)** | **NA** | **NA** | **Y (23)** | **Y (29)** | **NA** | **2 studies** |
| *Silene vulgaris* | C vs. R | Y (3) | NA | NA | NA | NA | Kováčik *et al.,* 2010 |
| *Silene vulgaris* | C vs. R | Y (1) | Y (8) | NA | NA | NA | Schat *et al.,* 1996 |
| *Silene vulgaris* | C vs. R | Y (3, 5) | NA | NA | NA | NA | Wierzbicka and Panufnik 1998 |
| *Taraxacum officinale* | C vs. R | NA | NA | Likely (23) | Likely (29) | NA | Keane *et al.,* 2005 |
| T*hlaspi caerulescens* | C vs. R | Y (bps) | NA | Y (23) | Y (29) | NA | Besnard *et al.,* 2009 |
| T*hlaspi caerulescens* | C vs. R | Y (bps) | NA | N (23) | NA | NA | Koch *et al.,* 1998 |
| *Typha latifolia* | C vs. R | NA | NA | Y (23) | Y (29) | NA | Keane *et al.,* 1999 |
| *Viola tricolor* | C vs. R | NA | NA | Y (23, 27) | Y (27) | NA | Slomka *et al.,* 2011 |
| **Bryophyta** |  |  |  |  |  |  |  |
| *Ceratodon purpureus* | C vs. R | Y (2, 6) | NA | NA | NA | NA | Jules and Shaw 1994 |
| **Pynophyta** |  |  |  |  |  |  |  |
| *Picea abies* | C vs. R | Y (1) | NA | Y (23, 27) | Y (27) | NA | Bergmann and Hosius 1996 |
| *Picea abies* | C vs. R | NA | NA | Y (23) | NA | NA | Prus-Glowacki and Godzik 1995 |
| *Picea rubens* | C vs. R | NA | NA | Y (23) | Y (29) | NA | Bashalkhanov *et al.,* 2013 |
| *Pinus sylvestris* | C vs. R | N (6) | NA | NA | NA | NA | Gera's kin *et al.,* 2011 |
| *Pinus sylvestris* | C vs. R | NA | NA | Y (23) | NA | NA | Gera's kin *et al.,* 2010 |
| ***Pinus sylvestris*** | **52.9 33.5 (Europe)** | **N (6)** | **NA** | **Y (23)** | **NA** | **NA** | **2 studies** |
| *Pinus sylvestris* | C vs. R | NA | NA | Y (23) | NA | NA | Korshikov *et al.,* 2002 |
| *Pinus sylvestris* | C vs. R | NA | NA | Y (23, 27) | Y (27) | NA | Kuchma and Finkeldey 2011 |
| *Pinus sylvestris* | C vs. R | NA | NA | Y (23) | NA | NA | Prus-Glowacki *et al.,*2006 |
| *Pinus sylvestris* | C vs. R | NA | NA | Likely (23, 27) | Y (27) | NA | Prus-Glowacki *et al.,* 1999 |
| *Pinus sylvestris* | C vs. R | Y (bps) | NA | Y (23) | NA | NA | Wojnicka-Póltorak 1997 |
| ***Pinus sylvestris*** | **50.49 28.88 (Europe)** | **Y (bps)** | **NA** | **Y (23, 27)** | **Y (27)** | **NA** | **3 studies** |
| **INVERTEBRATES** |  |  |  |  |  |  |  |
| **Anellida** |  |  |  |  |  |  |  |
| *Aporectodea caliginosa* | C vs. R | Y (3,5) | Y (13) | NA | NA | NA | Givaudan *et al.,* 2014 |
| *Aporectodea chlorotica* | C vs. R | N (3) | N (13) | NA | NA | NA | Givaudan *et al.,* 2014 |
| *Aporectodea tuberculata* | C vs. R | NA | Y (13) | NA | NA | NA | Lukkari *et al.,* 2004 |
| *Cognettia sphagnetorum* | C vs. R | Y (1, 2, 5, 6) | NA | Y (23) | N (29) | Y | Haimi *et al.,* 2006 |
| *Cognettia sphagnetorum* | C vs. R | Y (1,5) | NA | NA | NA | Y | Salminen and Haimi 2001 |
| ***Cognettia sphagnetorum*** | **61.31 22.13 (Europe)** | **Y (1, 2, 5, 6)** | **NA** | **Y (23)** | **N (29)** | **Y** | **2 studies** |
| *Dendrobaena octahedra* | C vs. R | N (1, 2, 6) | Y (13) | NA | NA | NA | Bengtsson *et al.,* 1992 |
| *Dendrobaena octahedra* | C vs. R | Y (bps) | Y (16) | NA | NA | NA | Fisker *et al.,* 2013 |
| *Dendrobaena octahedra* | C vs. R | Y (1, 2, 4, 6) | NA | NA | NA | Y | Fisker *et al.,* 2011 |
| ***Dendrobaena octahedra*** | **58.27 16.5 (Europe)** | **Y (bps, 1, 2, 4, 6)** | **Y (16)** | **NA** | **NA** | **Y** | **2 studies** |
| *Dendrobaena octahedra* | C vs. R | Y (1, 2, 3, 6) | NA | NA | NA | NA | Rozen 2006 |
| *Dendrodrilus rubidus* | C vs. R | Y (1, 2, 3, 4, 5, 6) | NA | NA | Y (28) | NA | Arnold *et al.,* 2008 |
| *Dendrodrilus rubidus* | C vs. R | Y (3) | NA | NA | NA | NA | Button *et al.,* 2012 |
| *Eisenia fetida* | Lab pop | N (3) | Y and N (16) | NA | NA | NA | Brulle *et al.,* 2011 |
| *Eisenia fetida* | Lab pop | NA | Y (17) | NA | NA | NA | Brulle *et al.,* 2008 |
| *Eisenia fetida* | C vs. R | N (1, 2, 3) | NA | NA | NA | NA | Spurgeon and Hopkin 2000 |
| *Hediste diversicolor* | C | Y (1) | NA | Y (23, 27) | Y (27) | NA | Virgilio and Abbiati 2004 |
| *Limnodrilus hoffmeisteri* | C vs. R | Y (1) | Y (10) | NA | NA | NA | Martinez and Levinton 1996 |
| *Lumbricus castaneous* | C vs. R | Y (3) | NA | NA | NA | NA | Button *et al.,* 2012 |
| *Lumbricus rubellus* | N | N (1, 2, 3) | NA | Y (23) | NA | Y | Anderson *et al.,* 2013 |
| *Lumbricus rubellus* | C vs. R | Y (3) | NA | Y (23) | NA | NA | Andre *et al.,* 2010 |
| *Lumbricus rubellus* | C vs. R | Y (bps) | NA | Y (23) | Y and N (29) | NA | Kille *et al.,* 2013 |
| *Lumbricus rubellus* | C vs. R | Y (1) | NA | NA | NA | NA | Langdon *et al.,* 2009 |
| *Lumbricus rubellus* | C vs. R | N (1, 2, 3, 6) | NA | NA | NA | NA | Spurgeon and Hopkin 1999 |
| *Lumbricus rubellus* | C vs. R | NA | Y (17) | NA | NA | NA | Stürzenbaun *et al.,* 1998 |
| *Lumbricus rubellus* | C vs. R | NA | Y (16) | NA | NA | NA | Stürzenbaun *et al.,* 1998 |
| ***Lumbricus rubellus*** | **50.8 -3.77 (Europe)** | **NY (bps, 1, 2, 3, 6)** | **Y (16, 17)** | **Y (23)** | **YN (29)** | **Y** | **6 studies** |
| *Tubifex tubifex* | Lab pop | Y (1) | NA | NA | NA | NA | Vidal and Horne 2003 |
| **Arthropoda** |  |  |  |  |  |  |  |
| *Agelena labyrinthica* | Gradient | Y (3) | Y (13) | NA | NA | NA | Wilczek *et al.,* 2003 |
| *Amphibalanus variegatus* | C vs. R | Y (1, 3) | NA | N (23) | NA | NA | Gall *et al.,* 2013 |
| *Anopheles gambiae* | Lab pop | Y (1, 4, 6) | NA | Y (27) | Y (27) | NA | Mireji *et al.,* 2010 |
| *Attheyella crassa* | Lab pop | N (5) | NA | Y (23) | NA | Y | Gardeström *et al.,* 2008 |
| *Balanus glandula* | C vs. R | NA | NA | Y (23, 27) | Y (27) | NA | Ma *et al.,* 2000 |
| *Bathycletopsyllus sp.* | C | NA | NA | N (23) | NA | NA | Gregg *et al.,* 2010 |
| *Ceriodaphnia pulchella* | C vs. R | Y (1, 6) | NA | NA | NA | NA | Lopes *et al.,* 2005 |
| *Chironomus februarius* | C vs. R | Y (6) | N (12) | NA | NA | Y | Bahrndorff *et al.,* 2006 |
| *Chironomus riparius* | Lab pop | N (1, 4, 6) | NA | Y (23, 27) | Y (27) | Y | Nowak *et al.,* 2009 |
| *Chironomus riparius* | Lab pop | Y (1, 6) | NA | Y (23) | NA | N | Vogt *et al.,* 2007 |
| ***Chironomus riparius*** | **Lab pop** | **YN (1, 4, 6)** | **NA** | **Y (23, 27)** | **Y (27)** | **YN** | **2 studies** |
| *Chironomus riparius* | Lab pop | N (1, 4, 6) | Y (9) | Y (23) | NA | NA | Nowak *et al.,* 2008 |
| *Chironomus riparius* | C vs. R | Y (1, 4) | Y (8) | NA | NA | NA | Groenendijk *et al.,* 2002 |
| *Chironomus riparius* | C vs. R | Y (2,3) | NA | NA | NA | NA | Postma *et al.,* 1996 |
| *Chironomus riparius* | C vs. R | Y, N (1, 3, 4, 5, 6) | NA | NA | NA | Y | Postma *et al.,* 1995 |
| *Chironomus riparius* | C vs. R | Y (1, 2, 4, 5) | NA | NA | NA | NA | Postma *et al.,* 1995 |
| *Chironomus riparius* | Not provided | Y (1, 3, 4, 6) | NA | NA | NA | Y | Postma and Davids 1995 |
| ***Chironomus riparius*** | **51.61 5.33 (Europe)** | **Y (1, 2, 3, 4)** | **Y (8)** | **NA** | **NA** | **Y** | **5 studies** |
| *Chironomus riparius* | C vs. R | Y (1) | NA | Y (23, 27) | Y (27) | NA | Soeter *et al.,* 2010 |
| *Daphnia longispina* | C vs. R | Y (1, 3) | Y (11) | NA | NA | NA | Agra *et al.,* 2010 |
| *Daphnia longispina* | C vs. R | Y (1, 5, 6) | NA | NA | NA | NA | Lopes *et al.,* 2006 |
| *Daphnia longispina* | C vs. R | Y (1, 3, 5, 6) | NA | NA | NA | NA | Lopes *et al.,* 2004 |
| *Daphnia longispina* | C vs. R | Y (1) | Y (9) | Likely (23, 27) | Y (27) | NA | Martins *et al.,* 2009 |
| *Daphnia longispina* | C vs. R | Y (1) | N (9) | N (23) | NA | NA | Martins *et al.,* 2007 |
| *Daphnia longispina* | C vs. R | Y (bps) | NA | Y (23, 27) | Y (27) | NA | Silva *et al.,* 2010 |
| ***Daphnia longispina*** | **37.7 -7.50 (Europe)** | **Y (bps, 1, 3, 5, 6)** | **YN (9)** | **YN (23, 27)** | **Y (27)** | **NA** | **5 studies** |
| *Daphnia magna* | Landscape with anthropogenic impact | Y (1) | NA | Y (23) | Y (28) | NA | Coors *et al.,* 2009 |
| *Daphnia magna* | Lab pop | Y (1, 3) | Y (13) | NA | NA | NA | Haap and Köhler 2009 |
| *Daphnia magna* | N vs. lab pop | N (1, 6) | Y (10) | NA | NA | NA | Messiaen *et al.,* 2013 |
| *Daphnia magna* | N | Y (6) | Y (10) | Y (22, 27) | Y (27) | NA | Messiaen *et al.,* 2012 |
| *Daphnia magna* | N | Y (1, 6) | Y (10) | NA | NA | Y | Messiaen *et al.,* 2010 |
| ***Daphnia magna*** | **51.05 2.71 (Europe)** | **YN (1, 6)** | **Y (10)** | **Y (22, 27)** | **Y (27)** | **Y** | **3 studies** |
| *Daphnia magna* | Lab pop | Y (1, 6) | NA | Y (23, 27) | Y (27) | NA | Ward and Robinson 2005 |
| *Daphnia pulex* | Lab pop | Y (3, 5, 6) | Y (16) | NA | NA | NA | Shaw *et al.,* 2007 |
| *Drosophila melanogaster* | Lab pop | Y (1, 4, 5, 6) | Y (8) | Y (27) | Y (27) | NA | Shirley and Sibly 1999 |
| *Drosophila subobscura* | C vs. R | Y (4, 6) | NA | NA | NA | NA | Kenig *et al.,* 2014 |
| *Drosophila subobscura* | C vs. R | Y (4, 6) | NA | NA | NA | NA | Kenig *et al.,* 2013 |
| ***Drosophila subobscura*** | **44.19 18.68 (Europe)** | **Y (4, 6)** | **NA** | **NA** | **NA** | **NA** | **2 studies** |
| *Drosophila subobscura* | N | Y (4, 5) | NA | Y (27) | Y (27) | NA | Kurbalija *et al.,* 2010 |
| *Folsomia candida* | Lab pop | Y (1) | Y (16) | NA | NA | NA | Nakamori *et al.,* 2010 |
| *Folsomia candida* | Lab pop | Y (6) | Y (16) | NA | NA | NA | Nota *et al.,* 2013 |
| *Folsomia candida* | Lab pop | Y (1,6) | Y (17) | NA | NA | NA | Nota *et al.,* 2010 |
| ***Folsomia candida*** | **Lab pop** | **Y (1, 6)** | **Y (16, 17)** | **NA** | **NA** | **NA** | **2 studies** |
| *Folsomia candida* | C vs. R | NA | Y (16) | NA | NA | NA | Nota *et al.,* 2011 |
| *Gammarus fossarum* | N | Y (1) | N (10) | NA | NA | NA | Chaumot *et al.,* 2009 |
| *Hyalella azteca* | Lab pop | Y (1) | Y (9) | Y (23, 27) | Y (27) | NA | Duan *et al.,* 2001 |
| *Hyalella azteca* | Lab pop | Y (1) | Y (9) | Y (23, 27) | Y (27) | NA | Duan *et al.,* 2000 II |
| ***Hyalella azteca*** | **Lab pop** | **Y (1)** | **Y (9)** | **Y (23, 27)** | **Y (27)** | **NA** | **2 studies** |
| *Hyalella azteca* | Lab pop vs. N | NA | Y (17, 18) | NA | NA | NA | Weston *et al.,* 2013 |
| *Isonychia bicolor* | C vs. R | Y (1, 5) | Y (9) | N (23) | NA | NA | Snyder and Hendricks 1997 |
| *Isotoma notabilis* | C vs. R | Contrasting (1, 2, 5, 6) | NA | NA | NA | Y | Tranvik *et al.,*1993 |
| *Kiefferulus intertinctus* | C vs. R | Y (6) | N (12) | NA | NA | Y | Bahrndorff *et al.,* 2006 |
| *Leander intermedius* | C vs. R | N (1) | Y (13) | N (23) | NA | NA | Ross *et al.,* 2002 |
| *Leptodiaptomus minutus* | C vs. R | N (1) | NA | Y (20, 27) | Y (27) | NA | Derry *et al.,* 2010 |
| *Microarthridion littorale* | N | Y (1) | NA | NA | Y (28) | NA | Schizas *et al.,* 2001 |
| *Nectopsyche albida* | N | Y (1) | NA | Y (23, 27) | Y (27) | NA | Benton *et al.,* 1992 |
| *Nitocra lacustris* | N | Not clear (1, 6) | NA | Y (23, 27) | Y (27) | Y | Street *et al.,* 1998 |
| *Onychiurus armatus* | C vs. R | Contrasting (1, 2, 5, 6) | NA | NA | NA | Y | Tranvik *et al.,* 1993 |
| *Orchesella bifasciata* | C vs. R | N (1, 3) | N (13) | NA | NA | NA | Köhler *et al.,* 1999 |
| *Orchesella cincta* | C vs. R | Y (1) | Y (9, 18) | Y (23) | NA | NA | Costa *et al.,* 2012 |
| *Orchesella cincta* | C vs. R | NA | NA | Y (23) | Y for one allele and Cu (29) | NA | Frati *et al.,* 1992 |
| *Orchesella cincta* | C | NA | Y (18) | Y (23) | Y (29) | NA | Janssens *et al.,* 2008 |
| *Orchesella cincta* | C vs. R | Y (bps) | Y (16, 18) | NA | NA | NA | Janssens *et al.,* 2007 |
| *Orchesella cincta* | C vs. R | Y (2, 3) | NA | NA | NA | NA | Posthuma *et al.,* 1992 |
| *Orchesella cincta* | C vs. R | Y (3) | Y (10) | NA | Y (28) | NA | Posthuma *et al.,* 1993 |
| *Orchesella cincta* | C vs. R | Y (1, 2, 3, 5, 6) | NA | NA | NA | NA | Posthuma *et al.,* 1993 |
| *Orchesella cincta* | C vs. lab pop | Y (bps) | Y (17) | NA | NA | NA | Roelofs *et al.,* 2009 |
| *Orchesella cincta* | C vs. R | NA | Y (17) | NA | NA | NA | Roelofs *et al.,* 2007 |
| *Orchesella cincta* | Lab pop | NA | Y (10, 16) | Y (23, 27) | Y (27) | NA | Roelofs *et al.,* 2006 |
| *Orchesella cincta* | C vs. lab pop | Y (bps) | Y (16) | Y (27) | Y (27) | NA | Sterenborg and Roelofs 2003 |
| *Orchesella cincta* | C vs. R | Y (bps) | NA | Y (24, 26) | Y (29) | NA | Timmermans *et al.,* 2007 |
| *Orchesella cincta* | C vs. R | Y (1) | Y (16) | NA | N (29) | NA | Timmermans *et al.,* 2005 |
| *Orchesella cincta* | C vs. R | NA | Y (16, 18) | NA | NA | NA | Van Straalen *et al.,* 2011 |
| ***Orchesella cincta*** | **51.57 10.93 (Europe)** | **Y (bps, 1, 2, 3, 5)** | **Y (9, 10, 16, 17, 18)** | **Y (23, 26, 27)** | **Y (27, 28, 29)** | **NA** | **14 studies** |
| *Pardosa lugubris* | Gradient | Y (3) | Y (13) | NA | NA | NA | Wilczek *et al.,* 2003 |
| *Pardrosa saltans* | C vs. R | N (3, 5, 6) | N (13) | NA | NA | NA | Eraly *et al.,* 2011 |
| *Pardrosa saltans* | C vs. R | N (1, 2, 3, 6) | Y (13) | NA | NA | NA | Eraly *et al.,* 2010 |
| ***Pardrosa saltans*** | **50.63 5.46 (Europe)** | **N (1, 2, 3, 5, 6)** | **YN (13)** | **NA** | **NA** | **NA** | **2 studies** |
| *Peramphithoe parmerong* | C vs. R | Y (1, 3, 5) | Y (8, 11) | NA | NA | NA | Pease *et al.,* 2010 |
| *Pirata piraticus* | C vs. R | Y (5, 6) | Y (8, 10) | NA | NA | NA | Hendrickx *et al.,* 2008 |
| *Pirata piraticus* | C vs. R | Y (6) | NA | NA | NA | NA | Hendrickx *et al.,* 2003 |
| ***Pirata piraticus*** | **50.68 3.77 (Europe)** | **Y (5, 6)** | **Y (8, 10)** | **NA** | **NA** | **NA** | **2 studies** |
| *Platynympha longicaudata* | C vs. R | Y (1) | Y (13) | N (23) | NA | NA | Ross *et al.,* 2002 |
| *Porcellio scaber* | C vs. R | Y (2, 3) | NA | NA | NA | NA | Donker *et al.,* 1996 |
| *Porcellio scaber* | C vs. R | Y (2, 3, 6) | NA | Y (27) | Y (27) | NA | Donker *et al.,* 1993 |
| ***Porcellio scaber*** | **51.27 5.55 (Europe)** | **Y (2, 3, 6)** | **NA** | **Y (27)** | **Y (27)** | **NA** | **2 studies** |
| *Spodoptera exigua* | Lab pop | Y (1,3) | NA | NA | NA | NA | Kafel *et al.,* 2014 |
| *Spodoptera exigua* | Lab pop | Y (1, 3, 4, 5) | NA | NA | NA | NA | Kafel *et al.,* 2012 |
| ***Spodoptera exigua*** | **Lab pop** | **Y (1,3, 4, 5)** | **NA** | **NA** | **NA** | **NA** | **2 studies** |
| *Tetrix tenuicornis* | C vs. R | Y (3) | NA | Y (23) | NA | NA | Grzywacz *et al.,*2012 |
| *Thamnocephalusplatyurus* | C vs. R | Y (1) | NA | NA | NA | NA | Brausch and Smith 2009 |
| *Tigriopus angulatus* | Lab pop | Y and N (1, 6) | NA | Y (27) | Y (27) | Y | Medina *et al.,* 2009 |
| **Bryozoa** |  |  |  |  |  |  |  |
| *Bugula neritina* | C vs. R | Y (1, 4) | Y (12) | NA | NA | Y | Piola and Johnston 2006 |
| *Celleporella hyalina* | N | Y (1, 2, 6) | NA | NA | NA | Y | Pistevos *et al.,* 2011 |
| *Watersipora subtorquata* | C vs. R | N (1, 4) | Y (11) | NA | NA | NA | McKenzie *et al.,* 2011 |
| **Chordata** |  |  |  |  |  |  |  |
| *Styela plicata* | N | Y and N (4) | It depends on the degree of stress (10) | NA | NA | NA | Galletly *et al.,* 2007 |
| **Cnidaria** |  |  |  |  |  |  |  |
| *Nematostella vectensis* | Lab pop | NA | Y (17) | NA | NA | NA | Elran *et al.,* 2014 |
| **Echinodermata** |  |  |  |  |  |  |  |
| *Centrostephanus rodgersii* | N | Y (1, 4, 6) | Y (10) | Y (21) | Y (29) | NA | Foo *et al.,* 2012 |
| *Strongylocentrotus franciscanus* | N | Y (4) | Y (10) | Y (27) | Y (27) | NA | Sunday *et al.,* 2011 |
| *Strongylocentrotus purpuratus* | N | Y (4) | Y (10) | Y (27) | Y (27) | Y | Kelly *et al.,* 2013 |
| *Strongylocentrotus purpuratus* | N | NA | NA | Y (23, 26, 27) | Y (27) | NA | Pespeni *et al.,* 2013 |
| **Mollusca** |  |  |  |  |  |  |  |
| *Biomphalaria glabrata* | Lab pop | Y (1, 4, 6) | NA | NA | NA | N | Salice *et al.,* 2010 |
| *Cantareus aspersus* | C vs. R | Y (3) | N (12) | NA | NA | NA | Fritsch *et al.,* 2011 |
| *Cassostrea gigas* | C vs. R | Y (3) | Y (16) | NA | Y (29) | NA | David *et al.,* 2012 |
| *Cassostrea gigas* | C vs. R | NA | Y (17) | NA | NA | NA | David *et al.,* 2007 |
| *Cassostrea gigas* | N | N (1) | NA | Y (23) | Y (29) | NA | Tanguy *et al.,* 1999 |
| ***Cassostrea gigas*** | **44.88 -1.59 (Europe)** | **YN (1, 3)** | **Y (16, 17)** | **Y (23)** | **Y (29)** | **NA** | **3 studies** |
| *Cassostrea angulata* | C vs. R | Y (3) | Y (13) | Y (27) | Y (27) | NA | Funes *et al.,* 2006 |
| *Cepaea nemoralis* | C vs. R | N (3) | N (12) | NA | NA | NA | Fritsch *et al.,* 2011 |
| *Dreissena polymorpha* | C vs. R | NA | Y (17) | N (23) | Y (28) | NA | Navarro *et al.,* 2013 |
| *Helisoma trivolvis* | C vs. R | Y (5) | Y (9) | Likely (23, 27) | Y (27) | NA | Benton *et al.,* 1994 |
| *Lymanea stagnalis* | C vs. R | N (2, 6) | N (18) | N (27) | N (27) | NA | Bouétard *et al.,* 2014 |
| *Macoma balthica* | N | Y (1) | NA | Y (23) | NA | NA | Hummel *et al.,* 1997 |
| *Macoma balthica* | C vs. R | Y (3, 5) | NA | N (23) | NA | NA | Sokolowski *et al.,* 2002 |
| *Mytilus edulis* | C vs. R | Y (1, 4) | N (8) | NA | NA | NA | Hoare *et al.,* 1995 |
| *Mytilus galloprovincialis* | C vs. R | N (3) | Y (13) | Y (27) | Y (27) | NA | Funes *et al.,* 2006 |
| *Mytilus galloprovincialis* | C vs. R | NA | NA | Y (23, 27) | Y (27) | NA | Ma *et al.,* 2000 |
| *Mytilus galloprovincialis* | C vs. R | NA | NA | Y (23) | N (28) | NA | Štambuk *et al.,* 2013 |
| *Mytilus galloprovincialis* | N | Y (5) | Y (17) | NA | NA | NA | Venier *et al.,* 2006 |
| *Mytilus trossulus* | N | N (4) | N (10) | Y (27) | Y (27) | NA | Sunday *et al.,* 2011 |
| *Nucella lapillus* | C vs. R | Y (5) | NA | N (23) | NA | NA | Plejdrup *et al.,* 2006 |
| *Perna viridis* | C vs. R | Y (3, 5) | NA | Y (23) | Y (28) | NA | Yap *et al.,* 2013 |
| *Perna viridis* | C vs. R | Y (3) | NA | Y (23) | Y (29) | NA | Yap *et al.,* 2004 |
| ***Perna viridis*** | **2.008 102.61 Asia)** | **Y (3, 5)** | **NA** | **Y (23)** | **Y (28, 29)** | **NA** | **2 studies** |
| *Ruditapes decussatus* | C vs. R | NA | Y (13, 18) | Y (23) | Y (28) | NA | Moraga *et al.,* 2002 |
| *Ruditapes philippinarum* | C vs. R | NA | Y (13, 18) | Y (23) | Y (28) | NA | Moraga *et al.,* 2002 |
| *Saccostrea glomerata* | N and lab pop (selection pop) | (1, 2, 4) | NA | NA | NA | NA | Parker *et al.,* 2012 |
| *Sphaerium novaezelandiae* | N | Y and N (1, 3) | NA | N (23) | NA | NA | Phillips and Hickey 2010 |
| **Nematoda** |  |  |  |  |  |  |  |
| *Caenorhabditis elegans* | Lab pop | N (1, 5, 6) | N (10) | N (27) | N (27) | NA | Dutilleul *et al.,* 2015 |
| *Caenorhabditis elegans* | Lab pop | Y (5, 6) | NA | Y (27) | Y (27) | Y | Dutilleul *et al.,* 2014 |
| *Caenorhabditis elegans* | Lab pop | N (1, 5, 6) | NA | N (27) | N (27) | NA | Dutilleul *et al.,* 2013 |
| ***Caenorhabditis elegans*** | **Lab pop** | **N (1, 5, 6)** | **N (10)** | **YN (27)** | **YN (27)** | **Y** | **3 studies** |
| **Platyhelminthes** |  |  |  |  |  |  |  |
| Polycelis tenuis | C vs. R | Y (1, 3, 5, 6) | NA | NA | Y (28) | NA | Indeherberg *et al.,* 1999 |
| **FISH** |  |  |  |  |  |  |  |
| **Chordata** |  |  |  |  |  |  |  |
| *Ameiurus nebulosus* | C vs. R | NA | NA | Maybe (23) | Y (27) | NA | Murdoch and Hebert 1994 |
| *Anguilla anguilla* | N | NA | NA | Y (23) | Y (32) | NA | Maes *et al.,* 2005 |
| *Anguilla anguilla* | Reads | NA | Y (17) | NA | NA | NA | Pujolar *et al.,* 2012 |
| ***Anguilla anguilla*** | **42.02 11.62 (Europe)** | **NA** | **Y (17)** | **Y (23)** | **Y (32)** | **NA** | **2 studies** |
| *Campostoma anomalum* | Varying H_2_O quality | NA | NA | Maybe (23, 27) | Y (27) | NA | Heithaus and Laushman 1997 |
| *Catostomus occidentalis* | C vs. R | NA | NA | N (23) | N (29) | NA | Whitehead *et al.,* 2003 |
| *Coregonus lavaretus* | C vs. R | Y (3, 5, 6) | NA | NA | NA | NA | Moiseenko 2002 |
| *Cyprinodon variegatus* | N | N (1) | N (10) | NA | NA | NA | Klerks and Moreau 2001 |
| *Danio rerio* | Pet farm | NA | Y (18) | NA | NA | NA | Sabri *et al.,* 2012 |
| *Etheostoma blennioides* | Varying H_2_O quality | NA | NA | Maybe (23, 27) | Y (27) | NA | Heithaus and Laushman 1997 |
| *Etheostoma caeruleum* | Varying H_2_O quality | NA | NA | Maybe (23, 27) | Y (27) | NA | Heithaus and Laushman 1997 |
| *Fundulus heteroclitus* | C vs. R | Y (1, 3, 4, 5) | N (17) | NA | NA | NA | Bozinovic and Oleksiak 2010 |
| *Fundulus heteroclitus* | C vs. R | Y (bps) | Y (16) | NA | NA | NA | Fisher and Oklesiak 2007 |
| *Fundulus heteroclitus* | C vs. R | Y (bps) | Y (18) | Y (23, 27) | Y (27) | NA | Hahn *et al.,* 2004 |
| *Fundulus heteroclitus* | C vs. R | NA | NA | Y (23) | NA | NA | Kirchhoff *et al.,* 1999 |
| *Fundulus heteroclitus* | C vs. R | Y (bps) | NA | N (23) | NA | NA | McMillan *et al.,* 2006 |
| *Fundulus heteroclitus* | C vs. R | NA | N (16) | NA | NA | NA | Meyer *et al.,* 2003 |
| *Fundulus heteroclitus* | C vs. R | Y (3) | Y (13) | NA | NA | NA | Meyer *et al.,* 2003 |
| *Fundulus heteroclitus* | C vs. R | Y (1, 4) | NA | NA | NA | NA | Meyer and Di Giulio 2003 |
| *Fundulus heteroclitus* | C vs. R | N (1, 4, 6) | NA | NA | NA | NA | Monosson *et al.,* 1995 |
| *Fundulus heteroclitus* | C vs. R | NA | NA | Y (23) | Y (29) | NA | Mulvey *et al.,* 2002 |
| *Fundulus heteroclitus* | C vs. R | Y (1, 3, 4) | NA | NA | NA | NA | Nacci *et al.,* 2010 |
| *Fundulus heteroclitus* | C vs. R | Y (1) | NA | NA | NA | NA | Nacci *et al.,* 2002 |
| *Fundulus heteroclitus* | C vs. R | Y (1, 3) | NA | NA | NA | NA | Nacci *et al.,* 1999 |
| *Fundulus heteroclitus* | C vs. R | Y (bps) | Y (17) | NA | NA | NA | Oleksiak *et al.,* 2011 |
| *Fundulus heteroclitus* | C vs. R | Y (3) | NA | NA | NA | NA | Ownby *et al.,* 2002 |
| *Fundulus heteroclitus* | C vs. R | Y (bps) | NA | Y (23, 27) | Y (27) | NA | Reitzel *et al.,* 2014 |
| *Fundulus heteroclitus* | C vs R | Y (bps) | NA | N (23, 27) | Y (27) | NA | Roark *et al.,* 2005 |
| *Fundulus heteroclitus* | C vs. R | Y (1) | Y (17) | NA | NA | NA | Whitehead *et al.,* 2012 |
| *Fundulus heteroclitus* | C vs. R | Y (1, 4) | Y (16) | NA | NA | NA | Whitehead *et al.,* 2010 |
| *Fundulus heteroclitus* | C vs. R | NA | NA | Y (23) | Y (29) | NA | Williams and Oleksiak 2011 |
| *Fundulus heteroclitus* | C vs. R | Y (bps) | NA | Y (23, 27) | Y (27) | NA | Williams and Oleksiak 2008 |
| ***Fundulus heteroclitus*** | **36.8 -76.44 (USA)** | **Y (bps, 1, 3, 4, 5, 6)** | **YN (13, 16, 17, 18)** | **YN (23, 27)** | **Y (27, 29)** | **NA** | **21 studies** |
| *Fundulus heteroclitus* | C vs. R | Y (4) | NA | NA | NA | NA | Elskus *et al.,* 1999 |
| *Gambusia affinis* | C vs. R | Y (bps) | NA | Y (23, 27) | Y (27, 30) | NA | Theodorakis *et al.,* 1999 |
| *Gambusia affinis* | C vs. R | NA | NA | Y (23 27) | Y (27) | NA | Theodorakis *et al.,* 1998 |
| *Gambusia affinis* | C vs. R | Y (6) | NA | Y (23) | Y (30) | NA | Theodorakis *et al.,* 1997 |
| *Gambusia affinis* | 35.93 -84.31 (USA) | Y (bps, 6) | NA | Y (**23, 27**) | Y (**27, 30**) | NA | 3 studies |
| *Gambusia holbrooki* | C vs. R | Y (5) | Y (9) | Likely (23) | Y (28) | NA | Benton *et al.,* 1994 |
| *Gambusia holbrooki* | C vs. R | Y (1) | NA | Y (23, 27) | Maybe (27) | NA | Keklak *et al.,* 1994 |
| *Gambusia holbrooki* | N | Y (5, 6) | Y (9) | Y (23, 27) | Y (27) | NA | Mulvey *et al.,* 1995 |
| *Gambusia holbrooki* | C vs. R | NA | NA | Y (23, 27) | Y (27) | NA | Theodorakis *et al.,* 1998 |
| *Gasterosteus aculeatus* | C vs. R | NA | NA | Y (23, 27) | Y (27) | NA | Lind and Grahn 2011 |
| *Gillichthys mirabilis* | C vs. C | NA | N (12) | NA | NA | NA | Forrester *et al.,* 2003 |
| *Gobio gobio* | C vs. R | Y (bps) | NA | Y (23) | N (31) | NA | Knapen *et al.,* 2009 |
| *Gobio gobio* | C vs. R | Y (bps) | Y (13, 16) | NA | NA | NA | Knapen *et al.,* 2007 |
| *Gobio gobio* | C vs. R | Y (1, 3) | Y (13) | NA | NA | NA | Knapen *et al.,* 2004 |
| ***Gobio gobio*** | **51.13 4.59 (Europe)** | **Y (bps, 1, 3)** | **Y (13, 16)** | **Y (23)** | **N (31)** | **NA** | **3 studies** |
| *Gobionellus boleosoma* | C vs. R | N (1) | NA | N (23) | NA | NA | Klerks *et al.,* 1997 |
| *Heterandria formosa* | Selection pop vs. lab pop | NA | NA | Y (23, 27) | Y (27) | NA | Athrey *et al.,* 2007 |
| *Heterandria formosa* | Selection pop | Y (1) | Y (10) | Y (27) | Y (27) | NA | Xie and Klerks 2003 |
| *Lepomis auritus* | C vs. R | NA | NA | Y (23) | Y (29) | NA | Nadig *et al.,* 1998 |
| *Leuciscus cephalus* | C vs. R | NA | NA | Y (23, 27) | Y (27) | NA | Larno *et al.,* 2001 |
| *Microgadus tomcod* | C vs. R | NA | Y (16) | Y (27) | Y (27) | NA | Wirgin *et al.,* 2011 |
| *Perca flavescens* | C vs. R | NA | Y (17) | NA | Y (29) | NA | Bélanger-Deschênes *et al.,* 2013 |
| *Pimephales promelas* | N | Y (1, 5) | Y (9) | Y (23) | Y (28) | NA | Schlueter *et al.,* 1995 |
| *Salmo trutta* | C vs. C-R | NA | NA | Y (23) | NA | NA | Durrant *et al.,* 2011 |
| *Salmo trutta* | C vs. R | NA | NA | Y (23) | NA | NA | Olsvik *et al.,* 2001 |
| *Solea Solea* | C vs. R | Y (5) | N (18) | Y (23) | Y (30) | NA | Guinand *et al.,* 2013 |
| *Umbra limi* | C vs. R | Y (1) | NA | Y (23, 27) | Y (27) | NA | Kopp *et al.,* 1992 |
| **AMPHIBIANS** |  |  |  |  |  |  |  |
| **Chordata** |  |  |  |  |  |  |  |
| *Rana arvalis* | C vs. R | Y (1, 4) | NA | Y (19) | Y, it depends on the trait (28) | NA | Hangartner *et al.,* 2011 |
| *Rana arvalis* | C vs. R | Y (1, 2, 4) | Y (8, 10) | NA | NA | NA | Merilä *et al.,* 2004 |
| *Rana arvalis* | C vs. R | Y (1, 4) | NA | Y (21, 27) | Y (27) | NA | Räsänen *et al.,* 2003 |
| *Rana temporaria* | N | N (1, 5) | Y but low (10) | NA | NA | NA | Pakkasmaa *et al.,* 2003 |
| ***Rana arvalis*** | **59.02 11.91 (Europe)** | **YN (1, 2, 4, 5)** | **Y (8, 10)** | **Y (19, 21, 27)** | **Y (27, 28)** | **NA** | **4 studies** |
| *Rana temporaria* | C vs. R | N (3) | NA | NA | NA | NA | Marquis *et al.,* 2009 |
| **MICROALGAE** |  |  |  |  |  |  |  |
| **Chlorophyta** |  |  |  |  |  |  |  |
| *Chlamydomonas cf. fonticola* | C vs. R | Y (3) | NA | Y (27) | Y (27) | Y | Garcia-Balboa *et al.,* 2013 |
| *Chlamydomonas reinhardtii* | C vs. R | Y (3) | NA | Y (27) | Y (27) | Y | Garcia-Balboa *et al.,* 2013 |
| *Dictyosphaerium chlorelloides* | Lab pop | Y (1) | NA | Y (27) | Y (27) | Y | Costas *et al.,* 2001 |
| *Dictyosphaerium chlorelloides* | C vs. R | Y (3) | NA | Y (27) | Y (27) | Y | Garcia-Balboa *et al.,* 2013 |
| *Dictyosphaerium chlorelloides* | N | Y (3) | NA | Y (27) | Y (27) | Y | Lopez-Rodas *et al.,* 2008 |
| *Dictyosphaerium chlorelloides* | C vs. R | NA | NA | Y (27) | Y (27) | Y | Marvá *et al.,* 2010 |
| *Scenedesmus intermedius* | Lab pop | NA | NA | Y (27) | Y (27) | Y | Baos *et al.,* 2002 |
| ***Rana arvalis*** | **59.02 11.91 (Europe)** | **YN (1, 2, 4, 5)** | **Y (8, 10)** | **Y (19, 21, 27)** | **Y (27, 28)** | **NA** | **5 studies** |
| *Scenedesmus intermedius* | C vs. R | NA | NA | Y (27) | Y (27) | Y | Marvá *et al.,* 2010 |
| **Cyanobacteria** |  |  |  |  |  |  |  |
| *Microcystis aeruginosa* | Lab pop | Y (5) | NA | Y (27) | Y (27) | Y | Garcia-Villada 2004 |
| *Microcystis aeruginosa* | Lab pop | Y (3, 5) | NA | NA | NA | Y | Polyak *et al.,* 2013 |
| **Dinophyta** |  |  |  |  |  |  |  |
| *Alexandrium minutum* | Lab pop | Y (3) | NA | Y (27) | Y (27) | Y | Flores-Moya *et al.,* 2012 |
| **Haptophyta** |  |  |  |  |  |  |  |
| *Emiliania huxleyi* | N | Y (3) | NA | Y (27) | Y (27) | Y | Lohbeck *et al.,* 2012 |
| *Emiliania huxleyi* | Lab pop | Y (3, 5) | NA | Y (27) | Y (27) | Y | Schlüter *et al.,* 2014 |
| *Gephyrocapsa oceanica* | Lab pop | Y (3, 5) | NA | Y (27) | Y (27) | Y | Jin *et al.,* 2013 |
| **Ocrophyta** |  |  |  |  |  |  |  |
| *Gomphonema parvulum* | C vs. R | Y (3) | NA | NA | NA | Y | Ivorra *et al.,* 2002 |
| **MACROALGAE** |  |  |  |  |  |  |  |
| **Hetokonta** |  |  |  |  |  |  |  |
| *Ectocarpus siliculosus* | C vs. R | Y (3) | Y (14) | NA | NA | NA | Ritter *et al.,* 2010 |
| *Fucus serratus* | C vs. R | Y (2, 3, 4) | NA | NA | NA | NA | Nielsen *et al.,* 2003 |

**Table S3.** Response variable and fixed factors included in the mixed-model analysis. The dataset included a large variety of experimental designs that were mainly classified as either "field" and "experiment" studies. The "field" studies involved direct measurements on traits from populations sampled from contaminated or reference sites. In this case we used the metal concentration measured in the contaminated site to calculate the effect sizes and we considered as control the population(s) from the reference site and as treatment the population(s) from contaminated habitats. The experiment studies involved manipulations under laboratory conditions where populations from contaminated and reference sites could be studied for their responses in both pristine and contaminated environments. In this case we used the response at concentration 0 as the control and the response at higher concentrations as the treatments for the calculations of effect sizes.

|  | |
| --- | --- |
| **Response variable** | Hedge's d effect size |
| **Metal concentration (ppm)** | Continuous: [metal]/threshold |
| **Metal** | type of metal (Cd, Cu, Pb, Zn) |
| **Subclass** | subclass name |
| **Phylum** | phylum name |
| **Habitat** | terrestrial-freshwater-marine |
| **Other metals** | yes-possible-no |
| **Exp or field** | exp-field |
| **Exp length** | Number of days |

**Table S4**. Selection table with second-order Akaike information criterion (AICc) and log-likelihoods (LL). Fixed terms: metal concentration (C), experiment or field factor (E), habitat (H), metal (M), subclass (S), phylum (P), presence of other metals (O). Metal concentration is ln([ppm]/threshold specific to habitat) and “na” stands for not applicable. ∆AICc values refer to the AICc-best models (in bold).

|  |  |  |  |  |  | |  | |  |
| --- | --- | --- | --- | --- | --- | --- | --- | --- | --- |
|  | **ID** | **Fixed terms** | **Random terms** | **LL** | | **AICc** | | **∆AICc** |  |
|  | **Weight** |  |  |  | |  | |  |  |
|  | Null model | na | na | -38.22 | | 78.67 | | 46.26 |  |
|  | Random model | na | Study ID | -12.41 | | **32.41** | | 0 |  |
|  | Mixed model (one factor) | C | Study ID | -12.49 | | 32.57 | | 0.16 |  |
|  | Mixed model (2 factors) | C + H | Study ID | -10.91 | | 37.28 | | 4.87 |  |
|  | Mixed model (3 factors) | C + H + E | Study ID | -10.37 | | 42.08 | | 9.67 |  |
|  | **No. of neonates** |  |  |  | |  | |  |  |
|  | Null model | na | na | -211.21 | | 424.54 | | 306.04 |  |
|  | Random model | na | Study ID | -62.52 | | 131.77 | | 13.27 |  |
|  | Mixed model (one factor) | C | Study ID | -60.27 | | 127.27 | | 8.77 |  |
|  | Mixed model (2 factors) | C + O | Study ID | -52.51 | | 120.13 | | 1.63 |  |
|  | Mixed model (3 factors) | C + O + M | Study ID | -45.96 | | **118.50** | | 0 |  |
|  | **Body metal content** |  |  |  | |  | |  |  |
|  | Null model | na | na | -180.25 | | 362.56 | | 187.51 |  |
|  | Random model | na | Study ID | -89.34 | | 185.09 | | 10.04 |  |
|  | Mixed model (one factor) | C | Study ID | -93.61 | | 193.63 | | 18.58 |  |
|  | Mixed model (2 factors) | C + S | Study ID | -74.25 | | 176.65 | | 1.60 |  |
|  | Mixed model (3 factors) | C + S + M | Study ID | -67.85 | | **175.05** | | 0 |  |
|  |  |  |  |  |  | |  | |  |

**Table S5.** Model-averaged coefficients from AICc-best models for weight, number of neonates and body metal content. Heterogeneity (Tau-squared), ICC (rho) and residual heterogeneity are also shown. Study ID was the random term in all models and random effects for the different metal concentrations tested within the same study were correlated through a multivariate parameterization. Fixed terms: metal concentration (C), habitat (H), metal (M), subclass (S), presence of other metals (O).

| **Weight** (Random model) | |  | **No. of neonates** (C+O+M) |  |  | **Body metal content** (C+S+M) | |  |
| --- | --- | --- | --- | --- | --- | --- | --- | --- |
|  | **Estimate** | **SE** | **Fixed terms** | **Estimate** | **SE** | **Fixed terms** | **Estimate** | **SE** |
| Intercept | 0.12 | 0.16 | ln([Metal]/threshold) | -0.26 | 0.15 | ln([Metal]/threshold) | 0.19 | 0.08 |
|  |  |  | Other metals (no) | -3.11 | 1.53 | Subclass (Collembola) | 0.58 | 0.60 |
|  |  |  | Other metals (possible presence) | -1.93 | 1.49 | Subclass (Eumalacostraca) | 1.89 | 0.52 |
|  |  |  | Other metals (yes) | 0.32 | 0.98 | Subclass (Oligochaeta) | 1.94 | 0.42 |
|  |  |  | Metal (Cd) | 1.68 | 1.63 | Subclass (Orthogastropoda) | 1.91 | 0.74 |
|  |  |  | Metal (Cu) | 2.43 | 1.41 | Subclass (Pteriomorphia) | -0.07 | 0.94 |
|  |  |  | Metal (Zn) | 2.03 | 1.77 | Subclass (Pterygota) | 1.82 | 0.63 |
|  |  |  |  |  |  | Subclass (Trepaxonemata) | -0.92 | 0.63 |
|  |  |  |  |  |  | Metal (Cd) | -0.25 | 0.46 |
|  |  |  |  |  |  | Metal (Cu) | -1.71 | 0.61 |
|  |  |  |  |  |  | Metal (Pb) | -1.58 | 0.60 |
|  |  |  |  |  |  | Metal (Zn) | -1.19 | 0.51 |
|  |  |  |  |  |  |  |  |  |
| *ICC* | 0.89 |  | *ICC (rho)* | 0.52 |  | *ICC (rho)* | -0.11 |  |
| *Heterogeneity* | 0.23 |  | *Heterogeneity* | 1.40 |  | *Heterogeneity* | 0.44 |  |
|  |  |  | *Residual heterogeneity* | Qe= 291.15 | Df= 31 | *Residual heterogeneity* | Qe= 168.15 | Df= 52 |

**Figure S1** All the typologies of pollution and their relative prevalence found in the studies and grouped as "other".


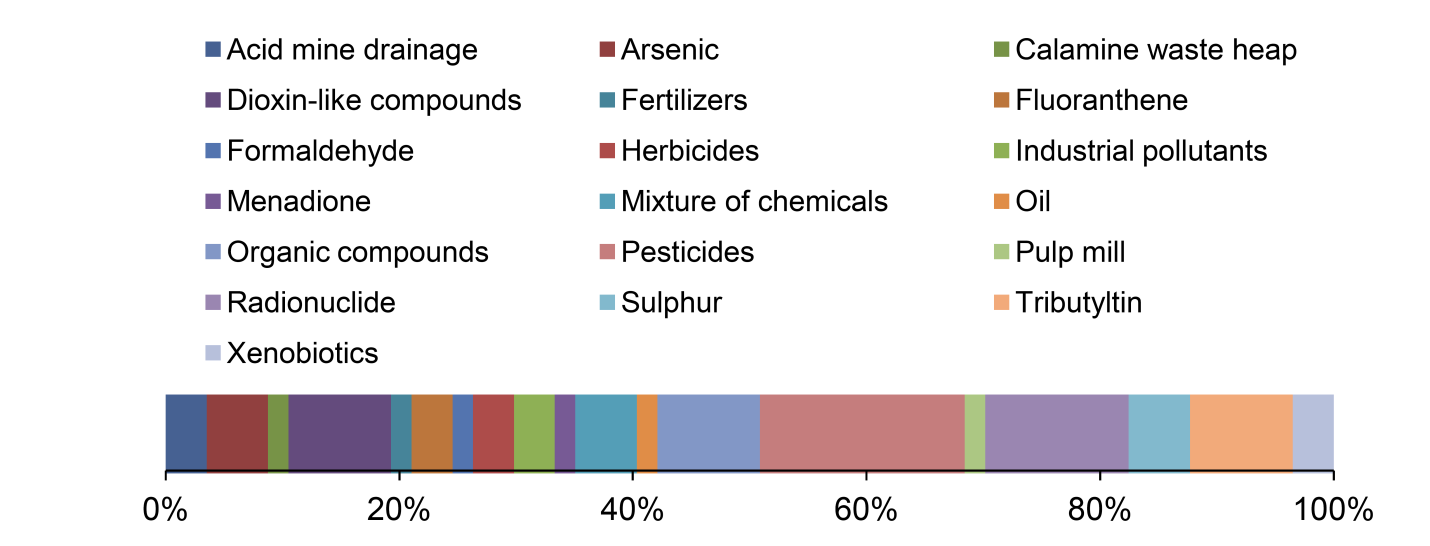


**Figure S2** Number of species and number of articled reviewed per phylum.

**
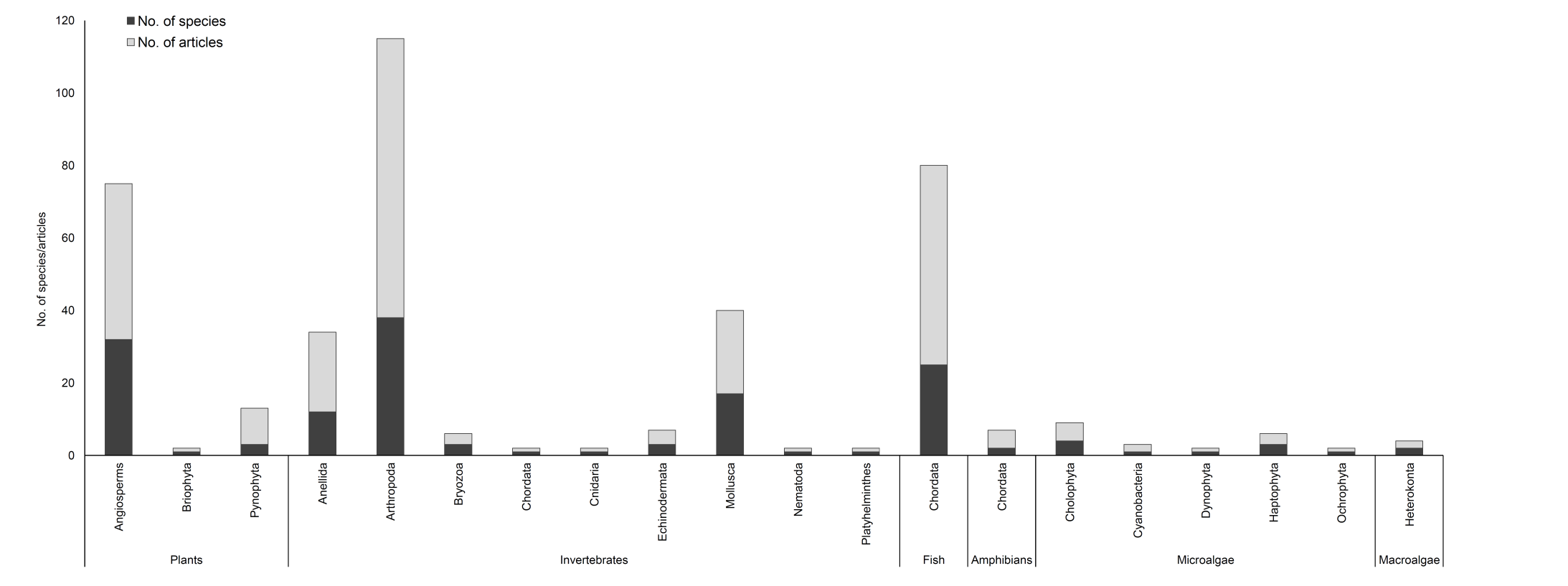
**

**Figure S3** Percentage of studies published between 1992 and 2014 that used molecular markers, genomics, transcriptomics and proteomics approaches to assess population genetic diversity, find evidence of selection and identify candidate functional genes under selection in the presence of pollution.


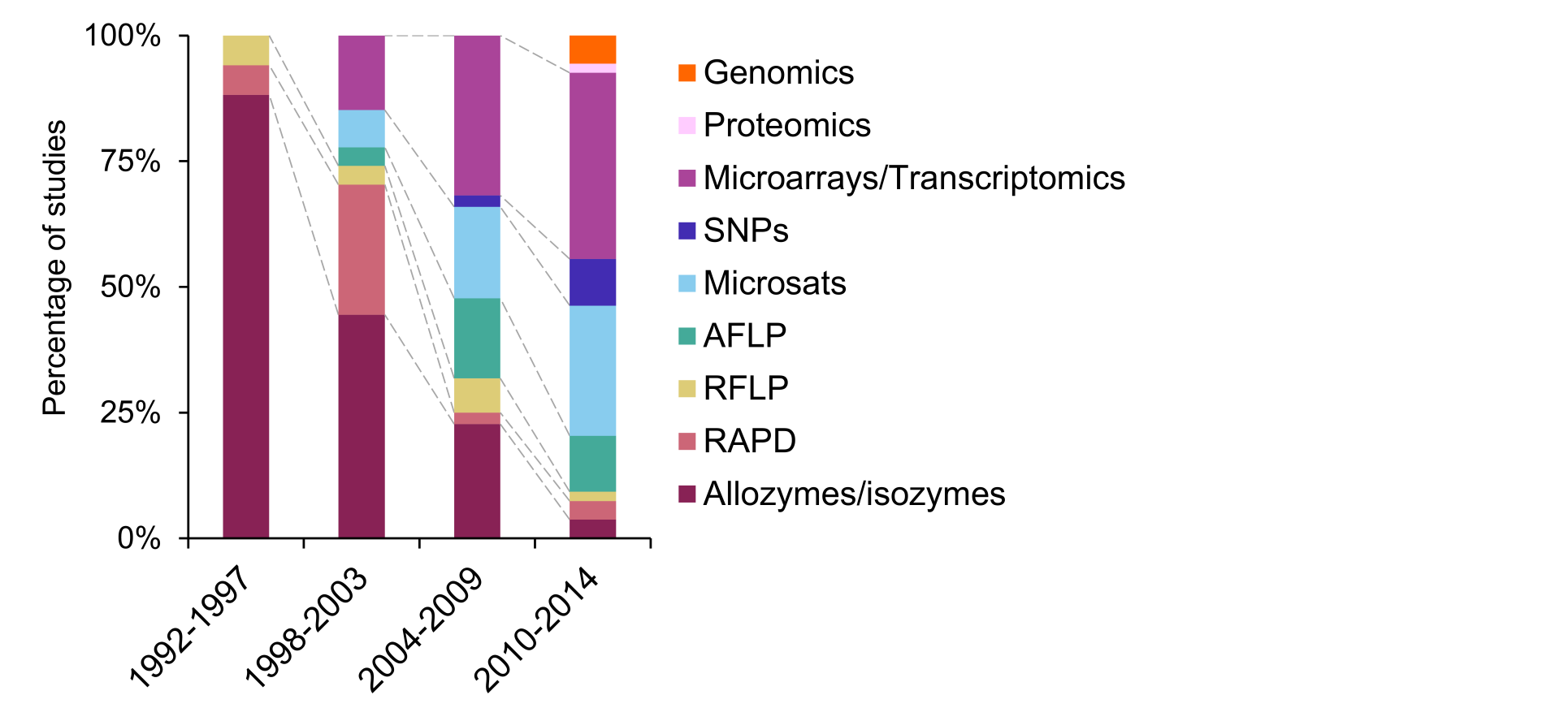


**Figure S4** Number of generations investigated by the reviewed studies.

**
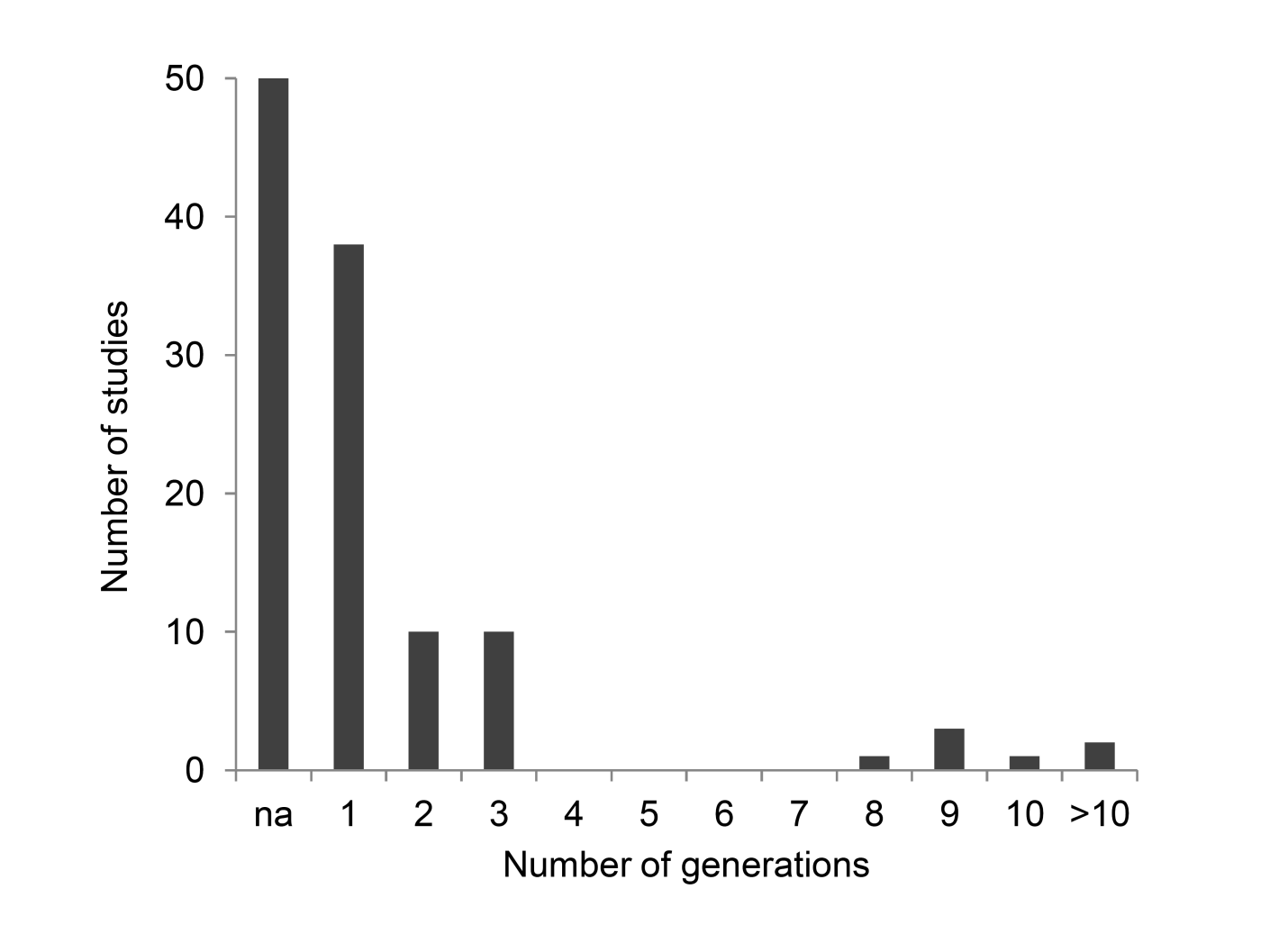
**

**References**

Agra, A.R., Guilhermino, L., Soares, A.M. & Barata, C. (2010). Genetic costs of tolerance to metals in *Daphnia longispina* populations historically exposed to a copper mine drainage. *Environmental Toxicology and Chemistry*, 29, 939-946.

Anderson, C., Kille, P., Lawlor, A. & Spurgeon, D.J. (2013). Life-history effects of arsenic toxicity in clades of the earthworm *Lumbricus rubellus*. *Environmental Pollution*, 172, 200-207.

Andre, J., King, R.A., Stürzenbaum, S., Kille, P., Hodson, M.E. & Morgan, A.J. (2010). Molecular genetic differentiation in earthworms inhabiting a heterogeneous Pb-polluted landscape. *Environmental Pollution*, 158, 883-890.

Arnold, R., Hodson, M. & Langdon, C. (2008). A Cu tolerant population of the earthworm *Dendrodrilus rubidus* (Savigny, 1862) at Coniston copper mines, Cumbria, UK. *Environmental Pollution*, 152, 713-722.

Athrey, N., Leberg, P.L. & Klerks, P.L. (2007). Laboratory culturing and selection for increased resistance to cadmium reduce genetic variation in the least killifish, *Heterandria formosa*. *Environmental Toxicology and Chemistry*, 26, 1916-1921.

Bahrndorff, S., Ward, J., Pettigrove, V. & Hoffmann, A.A. (2006). A microcosm test of adaptation and species specific responses to polluted sediments applicable to indigenous chironomids (Diptera). *Environmental Pollution*, 139, 550-560.

Baos, R., García-Villada, L., Agrelo, M., López-Rodas, V., Hiraldo, F. & Costas, E. (2002). Short-term adaptation of microalgae in highly stressful environments: an experimental model analysing the resistance of *Scenedesmus intermedius* (Chlorophyceae) to the heavy metals mixture from the Aznalcóllar mine spill. *European Journal of Phycology*, 37, 593-600.

Bashalkhanov, S., Eckert, A.J. & Rajora, O.P. (2013). Genetic signatures of natural selection in response to air pollution in red spruce (*Picea rubens*, Pinaceae). *Molecular Ecology*, 22, 5877-5889.

Becher, M., Talke, I.N., Krall, L. & Krämer, U. (2004). Cross‐species microarray transcript profiling reveals high constitutive expression of metal homeostasis genes in shoots of the zinc hyperaccumulator *Arabidopsis halleri*. *The Plant Journal*, 37, 251-268.

Bélanger-Deschênes, S., Couture, P., Campbell, P.G. & Bernatchez, L. (2013). Evolutionary change driven by metal exposure as revealed by coding SNP genome scan in wild yellow perch (*Perca flavescens*). *Ecotoxicology*, 22, 938-957.

Belfiore, N.M. & Anderson, S.L. (1998). Genetic patterns as a tool for monitoring and assessment of environmental impacts: the example of genetic ecotoxicology. *Environmental Monitoring and Assessment*, 51, 465-479.

Belfiore, N.M. & Anderson, S.L. (2001). Effects of contaminants on genetic patterns in aquatic organisms: a review. *Mutation Research/Reviews in Mutation Research*, 489, 97-122.

Bengtsson, G., Ek, H. & Rundgren, S. (1992). Evolutionary response of earthworms to long-term metal exposure. *Oikos*, 289-297.

Benton, M.J., Diamond, S.A. & Guttman, S.I. (1994). A genetic and morphometric comparison of *Helisoma trivolvis* and *Gambusia holbrooki* from clean and contaminated habitats. *Ecotoxicology and Environmental Safety*, 29, 20-37.

Benton, M.J. & Guttman, S.I. (1992). Allozyme genotype and differential resistance to mercury pollution in the caddisfly, *Nectopsyche albida*. I. Single-locus genotypes. *Canadian Journal of Fisheries and Aquatic Sciences*, 49, 142-146.

Bergmann, F. & Hosius, B. (1996). Effects of heavy metal polluted soils on the genetic structure of norway spruce seedling populations. *Water, Air, and Soil Pollution*, 89, 363-373.

Besnard, G., Basic, N., Christin, P.A., Savova‐Bianchi, D. & Galland, N. (2009). *Thlaspi caerulescens* (Brassicaceae) population genetics in western Switzerland: is the genetic structure affected by natural variation of soil heavy metal concentrations? *New Phytologist*, 181, 974-984.

Bouétard, A., Côte, J., Besnard, A.-L., Collinet, M. & Coutellec, M.-A. (2014). Environmental versus Anthropogenic Effects on Population Adaptive Divergence in the Freshwater Snail *Lymnaea stagnalis. Plos One*, 9, e106670.

Bozinovic, G. & Oleksiak, M.F. (2010). Embryonic gene expression among pollutant resistant and sensitive *Fundulus heteroclitus* populations. *Aquatic Toxicology*, 98, 221-229.

Brausch, J.M. & Smith, P.N. (2009). Pesticide resistance from historical agricultural chemical exposure in *Thamnocephalus platyurus* (Crustacea: Anostraca). *Environmental Pollution*, 157, 481-487.

Breckels, R.D. & Neff, B.D. (2010). Pollution-induced behavioural effects in the brown bullhead (*Ameiurus nebulosus*). *Ecotoxicology*, 19, 1337-1346.

Brulle, F., Cocquerelle, C., Mitta, G., Castric, V., Douay, F., Leprêtre, A. *et al.* (2008). Identification and expression profile of gene transcripts differentially expressed during metallic exposure in *Eisenia fetida* coelomocytes. *Developmental & Comparative Immunology*, 32, 1441-1453.

Brulle, F., Lemière, S., Waterlot, C., Douay, F. & Vandenbulcke, F. (2011). Gene expression analysis of 4 biomarker candidates in *Eisenia fetida* exposed to an environmental metallic trace elements gradient: a microcosm study. *Science of the Total Environment*, 409, 5470-5482.

Button, M., Koch, I. & Reimer, K.J. (2012). Arsenic resistance and cycling in earthworms residing at a former gold mine in Canada. *Environmental Pollution*, 169, 74-80.

Chaumot, A., Gos, P., Garric, J. & Geffard, O. (2009). Additive vs non-additive genetic components in lethal cadmium tolerance of *Gammarus* (Crustacea): Novel light on the assessment of the potential for adaptation to contamination. *Aquatic Toxicology*, 94, 294-299.

Chen, X.-y., Li, N., Shen, L. & Li, Y.-y. (2003). Genetic structure along a gaseous organic pollution gradient: a case study with *Poa annua* L. *Environmental Pollution*, 124, 449-455.

Coors, A., Vanoverbeke, J., De Bie, T. & De Meester, L. (2009). Land use, genetic diversity and toxicant tolerance in natural populations of *Daphnia magna*. *Aquatic toxicology*, 95, 71-79.

Costa, D., Mariën, J., Janssens, T.K., van Gestel, C.A., Driessen, G., Sousa, J.P. *et al.* (2012). Influence of adaptive evolution of cadmium tolerance on neutral and functional genetic variation in *Orchesella cincta*. *Ecotoxicology*, 21, 2078-2087.

Costas, E., Carrillo, E., Ferrero, L.M., Agrelo, M., Garcia-Villada, L., Juste, J. *et al.* (2001). Mutation of algae from sensitivity to resistance against environmental selective agents: the ecological genetics of *Dictyosphaerium chlorelloides* (Chlorophyceae) under lethal doses of 3-(3, 4-dichlorophenyl)-1, 1-dimethylurea herbicide. *Phycologia*, 40, 391-398.

Davey, J.W., Hohenlohe, P.A., Etter, P.D., Boone, J.Q., Catchen, J.M. & Blaxter, M.L. (2011). Genome-wide genetic marker discovery and genotyping using next-generation sequencing. *Nature Reviews Genetics*, 12, 499-510.

David, E., Tanguy, A. & Moraga, D. (2007). Peroxiredoxin 6 gene: a new physiological and genetic indicator of multiple environmental stress response in Pacific oyster *Crassostrea gigas*. *Aquatic Toxicology*, 84, 389-398.

David, E., Tanguy, A. & Moraga, D. (2012). Characterisation and genetic polymorphism of metallothionein gene CgMT4 in experimental families of Pacific oyster *Crassostrea gigas* displaying summer mortality. *Biomarkers*, 17, 85-95.

Deng, J., Liao, B., Ye, M., Deng, D., Lan, C. & Shu, W. (2007). The effects of heavy metal pollution on genetic diversity in zinc/cadmium hyperaccumulator *Sedum alfredii* populations. *Plant and Soil*, 297, 83-92.

Derry, A.M., Arnott, S.E. & Boag, P.T. (2010). Evolutionary shifts in copepod acid tolerance in an acid-recovering lake indicated by resurrected resting eggs. *Evolutionary Ecology*, 24, 133-145.

Donker, M., Zonneveld, C. & Van Straalen, N. (1993). Early reproduction and increased reproductive allocation in metal-adapted populations of the terrestrial isopod *Porcellio scaber*. *Oecologia*, 96, 316-323.

Donker, M.H., Raedecker, M.H. & Van Straalen, N.M. (1996). The role of zinc regulation in the zinc tolerance mechanism of the terrestrial isopod *Porcellio scaber*. *Journal of Applied Ecology*, 955-964.

Duan, Y., Guttman, S.I., Oris, J.T. & Bailer, A.J. (2001). Differential survivorship among allozyme genotypes of *Hyalella azteca* exposed to cadmium, zinc or low pH. *Aquatic Toxicology*, 54, 15-28.

Duan, Y., Guttman, S.I., Oris, J.T., Huang, X. & Burton, G.A. (2000). Genotype and toxicity relationships among *Hyalella azteca*: II. Acute exposure to fluoranthene‐contaminated sediment. *Environmental Toxicology and Chemistry*, 19, 1422-1426.

Durrant, C.J., Stevens, J.R., Hogstrand, C. & Bury, N.R. (2011). The effect of metal pollution on the population genetic structure of brown trout (*Salmo trutta* L.) residing in the River Hayle, Cornwall, UK. *Environmental Pollution*, 159, 3595-3603.

Dutilleul, M., Bonzom, J.-M., Lecomte, C., Goussen, B., Daian, F., Galas, S. *et al.* (2014). Rapid evolutionary responses of life history traits to different experimentally-induced pollutions in *Caenorhabditis elegans*. *BMC Evolutionary Biology*, 14, 252.

Dutilleul, M., Goussen, B., Bonzom, J.-M., Galas, S. & Réale, D. (2015). Pollution Breaks Down the Genetic Architecture of Life History Traits in *Caenorhabditis elegans*. *PloS One*, 10, e0116214.

Dutilleul, M., Lemaire, L., Réale, D., Lecomte, C., Galas, S. & Bonzom, J.-M. (2013). Rapid phenotypic changes in *Caenorhabditis elegans* under uranium exposure. *Ecotoxicology*, 22, 862-868.

Egerton-Warburton, L.M. (1995). An absence of ecotype evolution in three *Eucalyptus* species colonizing coal mine soils with low pH and high aluminium content. *Water, Air, and Soil Pollution*, 83, 335-349.

Elran, R., Raam, M., Kraus, R., Brekhman, V., Sher, N., Plaschkes, I. *et al.* (2014). Early and late response of *Nematostella vectensis* transcriptome to heavy metals. *Molecular Ecology*, 23, 4722-4736.

Elskus, A.A., Monosson, E., McElroy, A.E., Stegeman, J.J. & Woltering, D.S. (1999). Altered CYP1A expression in *Fundulus heteroclitus* adults and larvae: a sign of pollutant resistance? *Aquatic Toxicology*, 45, 99-113.

Eraly, D., Hendrickx, F., Backeljau, T., Bervoets, L. & Lens, L. (2011). Direct and indirect effects of metal stress on physiology and life history variation in field populations of a lycosid spider. *Ecotoxicology and Environmental Safety*, 74, 1489-1497.

Eraly, D., Hendrickx, F., Bervoets, L. & Lens, L. (2010). Experimental exposure to cadmium affects metallothionein-like protein levels but not survival and growth in wolf spiders from polluted and reference populations. *Environmental Pollution*, 158, 2124-2131.

Eränen, J. (2008). Rapid evolution towards heavy metal resistance by mountain birch around two subarctic copper–nickel smelters. *Journal of Evolutionary Biology*, 21, 492-501.

Fisher, M.A. & Oleksiak, M.F. (2007). Convergence and divergence in gene expression among natural populations exposed to pollution. *BMC Genomics*, 8, 108.

Fisker, K.V., Holmstrup, M. & Sørensen, J.G. (2013). Variation in metallothionein gene expression is associated with adaptation to copper in the earthworm *Dendrobaena octaedra*. *Comparative Biochemistry and Physiology Part C: Toxicology & Pharmacology*, 157, 220-226.

Fisker, K.V., Sørensen, J.G., Damgaard, C., Pedersen, K.L. & Holmstrup, M. (2011). Genetic adaptation of earthworms to copper pollution: is adaptation associated with fitness costs in *Dendrobaena octaedra*? *Ecotoxicology*, 20, 563-573.

Flores‐Moya, A., Rouco, M., García‐Sánchez, M.J., García‐Balboa, C., González, R., Costas, E. *et al.* (2012). Effects of adaptation, chance, and history on the evolution of the toxic dinoflagellate *Alexandrium minutum* under selection of increased temperature and acidification. *Ecology and Evolution*, 2, 1251-1259.

Foo, S.A., Dworjanyn, S.A., Poore, A.G. & Byrne, M. (2012). Adaptive capacity of the habitat modifying sea urchin *Centrostephanus rodgersii* to ocean warming and ocean acidification: performance of early embryos. *PLoS One*, 7, e42497.

Ford, M.J. (2002). Applications of selective neutrality tests to molecular ecology. *Molecular Ecology*, 11, 1245-1262.

Forrester, G.E., Fredericks, B.I., Gerdeman, D., Evans, B., Steele, M.A., Zayed, K. *et al.* (2003). Growth of estuarine fish is associated with the combined concentration of sediment contaminants and shows no adaptation or acclimation to past conditions. *Marine Environmental Research*, 56, 423-442.

Frati, F., Fanciulli, P.P. & Posthuma, L. (1992). Allozyme variation in reference and metal-exposed natural populations of *Orchesella cincta* (Insecta: Collembola). *Biochemical Systematics and Ecology*, 20, 297-310.

Frérot, H., Faucon, M.P., Willems, G., Godé, C., Courseaux, A., Darracq, A. *et al.* (2010). Genetic architecture of zinc hyperaccumulation in *Arabidopsis halleri*: the essential role of QTL× environment interactions. *New Phytologist*, 187, 355-367.

Fritsch, C., Coeurdassier, M., Gimbert, F., Crini, N., Scheifler, R. & De Vaufleury, A. (2011). Investigations of responses to metal pollution in land snail populations (*Cantareus aspersus* and *Cepaea nemoralis*) from a smelter-impacted area. *Ecotoxicology*, 20, 739-759.

Funes, V., Alhama, J., Navas, J., López-Barea, J. & Peinado, J. (2006). Ecotoxicological effects of metal pollution in two mollusc species from the Spanish South Atlantic littoral. *Environmental Pollution*, 139, 214-223.

Gall, M.L., Holmes, S.P., Dafforn, K.A. & Johnston, E.L. (2013). Differential tolerance to copper, but no evidence of population-level genetic differences in a widely-dispersing native barnacle. *Ecotoxicology*, 22, 929-937.

Galletly, B.C., Blows, M.W. & Marshall, D.J. (2007). Genetic mechanisms of pollution resistance in a marine invertebrate. *Ecological Applications*, 17, 2290-2297.

García-Balboa, C., Baselga-Cervera, B., García-Sanchez, A., Igual, J.M., Lopez-Rodas, V. & Costas, E. (2013). Rapid adaptation of microalgae to bodies of water with extreme pollution from uranium mining: An explanation of how mesophilic organisms can rapidly colonise extremely toxic environments. *Aquatic Toxicology*, 144, 116-123.

Garcı́a-Villada, L., Rico, M., Altamirano, M.a., Sánchez-Martı́n, L., López-Rodas, V. & Costas, E. (2004). Occurrence of copper resistant mutants in the toxic cyanobacteria *Microcystis aeruginosa*: characterisation and future implications in the use of copper sulphate as algaecide. *Water Research*, 38, 2207-2213.

Gardeström, J., Dahl, U., Kotsalainen, O., Maxson, A., Elfwing, T., Grahn, M. *et al.* (2008). Evidence of population genetic effects of long-term exposure to contaminated sediments—a multi-endpoint study with copepods. *Aquatic Toxicology*, 86, 426-436.

Geras’kin, S., Oudalova, A., Dikareva, N., Spiridonov, S., Hinton, T., Chernonog, E. *et al.* (2011). Effects of radioactive contamination on Scots pines in the remote period after the Chernobyl accident. *Ecotoxicology*, 20, 1195-1208.

Geras’kin, S., Vanina, J., Dikarev, V., Novikova, T., Oudalova, A. & Spiridonov, S. (2010). Genetic variability in Scotch pine populations of the Bryansk region radioactively contaminated in the Chernobyl accident. *Biophysics*, 55, 324-331.

Ginocchio, R., Toro, I., Schnepf, D. & Macnair, M. (2002). Copper tolerance testing in populations of *Mimulus luteus* var. variegatus exposed and non-exposed to copper mine pollution. *Geochemistry: Exploration, Environment, Analysis*, 2, 151-156.

Givaudan, N., Binet, F., Le Bot, B. & Wiegand, C. (2014). Earthworm tolerance to residual agricultural pesticide contamination: Field and experimental assessment of detoxification capabilities. *Environmental Pollution*, 192, 9-18.

Gregg, C.S., Foltz, D.W. & Fleeger, J.W. (2010). Genetic diversity in a deep-sea harpacticoid copepod found near two oil-drilling sites in the Gulf of Mexico. *Journal of Crustacean Biology*, 30, 651-657.

Groenendijk, D., Lücker, S.M., Plans, M., Kraak, M.H. & Admiraal, W. (2002). Dynamics of metal adaptation in riverine chironomids. *Environmental Pollution*, 117, 101-109.

Grzywacz, B., Warchałowska-Śliwa, E., Banach, Z. & Pyza, E. (2012). Genetic variability and changes of elemental concentrations in cells of *Tetrix tenuicornis* (Orthoptera: Tetrigidae) from polluted and unpolluted areas. *Folia biologica*, 60, 17-25.

Guinand, B., Fustier, M., Labonne, M., Jourdain, E., Calves, I., Quiniou, L. *et al.* (2013). Genetic structure and heterozygosity–fitness correlation in young-of-the-year sole (*Solea solea* L.) inhabiting three contaminated West-European estuaries. *Journal of Sea Research*, 80, 35-49.

Haap, T. & Köhler, H.-R. (2009). Cadmium tolerance in seven *Daphnia magna* clones is associated with reduced hsp70 baseline levels and induction. *Aquatic Toxicology*, 94, 131-137.

Hahn, M.E., Karchner, S.I., Franks, D.G. & Merson, R.R. (2004). Aryl hydrocarbon receptor polymorphisms and dioxin resistance in Atlantic killifish (*Fundulus heteroclitus*). *Pharmacogenetics and Genomics*, 14, 131-143.

Haimi, J., Knott, K.E., Selonen, S. & Laurikainen, M. (2006). Has long-term metal exposure induced changes in life history traits and genetic diversity of the enchytraeid worm *Cognettia sphagnetorum* (Vejd.). *Environmental Pollution*, 140, 463-470.

Hangartner, S., Laurila, A. & Räsänen, K. (2011). Adaptive divergence of the moor frog (*Rana arvalis*) along an acidification gradient. *BMC Evolutionary Biology*, 11, 366.

Hanikenne, M., Kroymann, J., Trampczynska, A., Bernal, M., Motte, P., Clemens, S. *et al.* (2013). Hard selective sweep and ectopic gene conversion in a gene cluster affording environmental adaptation. *PLoS Genetics*, 9, e1003707.

Hanikenne, M., Talke, I.N., Haydon, M.J., Lanz, C., Nolte, A., Motte, P. *et al.* (2008). Evolution of metal hyperaccumulation required cis-regulatory changes and triplication of HMA4. *Nature*, 453, 391-395.

Haque, N., Peralta-Videa, J., Duarte-Gardea, M. & Gardea-Torresdey, J. (2009). Differential effect of metals/metalloids on the growth and element uptake of mesquite plants obtained from plants grown at a copper mine tailing and commercial seeds. *Bioresource Technology*, 100, 6177-6182.

Heithaus, M.R. & Laushman, R.H. (1997). Genetic variation and conservation of stream fishes: influence of ecology, life history, and water quality. *Canadian Journal of Fisheries and Aquatic Sciences*, 54, 1822-1836.

Hendrickx, F., Maelfait, J.-P., Speelmans, M. & Van Straalen, N.M. (2003). Adaptive reproductive variation along a pollution gradient in a wolf spider. *Oecologia*, 134, 189-194.

Hendrickx, F., MAELFAIT, J.P. & Lens, L. (2008). Effect of metal stress on life history divergence and quantitative genetic architecture in a wolf spider. *Journal of Evolutionary Biology*, 21, 183-193.

Hoare, K., Beaumont, A. & Davenport, J. (1995). Variation among populations in the resistance of *Mytilus edulis* embryos to copper: adaptation to pollution? *Marine Ecology Progress Series. Oldendorf*, 120, 155-161.

Hoffmann, A.A. & Daborn, P.J. (2007). Towards genetic markers in animal populations as biomonitors for human‐induced environmental change. *Ecology Letters*, 10, 63-76.

Hoffmann, A.A., Sgro, C. & Lawler, S. (1995). Ecological population genetics: the interface between genes and the environment. *Annual Review of Genetics*, 29, 349-370.

Hoffmann, A.A. & Willi, Y. (2008). Detecting genetic responses to environmental change. *Nature Reviews Genetics*, 9, 421-432.

Hummel, H., Bogaards, R., Bek, T., Polishchuk, L., Amiard-Triquet, C., Bachelet, G. *et al.* (1997). Sensitivity to stress in the bivalve *Macoma balthica* from the most northern (Arctic) to the most southern (French) populations: low sensitivity in Arctic populations because of genetic adaptations? *Hydrobiologia*, 355, 127-138.

Indeherberg, M., Van Straalen, N. & Schockaert, E. (1999). Combining life-history and toxicokinetic parameters to interpret differences in sensitivity to cadmium between populations of *Polycelis tenuis* (Platyhelminthes). *Ecotoxicology and Environmental Safety*, 44, 1-11.

Ivorra, N., Barranguet, C., Jonker, M., Kraak, M.H. & Admiraal, W. (2002). Metal-induced tolerance in the freshwater microbenthic diatom *Gomphonema parvulum*. *Environmental Pollution*, 116, 147-157.

Janssens, T.K., Lopéz, R.d.R., Mariën, J., Timmermans, M.J., Montagne-Wajer, K., Van Straalen, N.M. *et al.* (2008). Comparative population analysis of metallothionein promoter alleles suggests stress-induced microevolution in the field. *Environmental Science & Technology*, 42, 3873-3878.

Janssens, T.K., Mariën, J., Cenijn, P., Legler, J., van Straalen, N.M. & Roelofs, D. (2007). Recombinational micro-evolution of functionally different metallothionein promoter alleles from *Orchesella cincta*. *BMC Evolutionary Biology*, 7, 88.

Jin, P., Gao, K. & Beardall, J. (2013). Evolutionary responses of a coccolithophorid *Gephyrocapsa oceanica* to ocean acidification. *Evolution*, 67, 1869-1878.

Jules, E.S. & Shaw, A.J. (1994). Adaptation to metal-contaminated soils in populations of the moss, *Ceratodon purpureus*: vegetative growth and reproductive expression. *American Journal of Botany*, 791-797.

Kafel, A., Rozpędek, K., Szulińska, E., Zawisza-Raszka, A. & Migula, P. (2014). The effects of cadmium or zinc multigenerational exposure on metal tolerance of *Spodoptera exigua* (Lepidoptera: Noctuidae). *Environmental Science and Pollution Research International*, 21, 4705.

Kafel, A., Zawisza-Raszka, A. & Szulińska, E. (2012). Effects of multigenerational cadmium exposure of insects (*Spodoptera exigua* larvae) on anti-oxidant response in haemolymph and developmental parameters. *Environmental Pollution*, 162, 8-14.

Keane, B., Collier, M.H. & Rogstad, S.H. (2005). Pollution and genetic structure of North American populations of the common dandelion (*Taraxacum officinale*). *Environmental Monitoring and Assessment*, 105, 341-357.

Keane, B., Pelikan, S., Toth, G.P., Smith, M.K. & Rogstad, S.H. (1999). Genetic diversity of *Typha latifolia* (Typhaceae) and the impact of pollutants examined with tandem-repetitive DNA probes. *American Journal of Botany*, 86, 1226-1238.

Keklak, M., Newman, M. & Mulvey, M. (1994). Enhanced uranium tolerance of an exposed population of the eastern mosquitofish (*Gambusia holbrooki* Girard 1859). *Archives of Environmental Contamination and Toxicology*, 27, 20-24.

Kelly, M.W., Padilla‐Gamiño, J.L. & Hofmann, G.E. (2013). Natural variation and the capacity to adapt to ocean acidification in the keystone sea urchin *Strongylocentrotus purpuratus*. *Global Change Biology*, 19, 2536-2546.

Kenig, B., Patenković, A., Anđelković, M. & Stamenković-Rada, M. (2014). Life-history variation of *Drosophila subobscura* under lead pollution depends on population history. *Genetika*, 46, 693-703.

Kenig, B., Stamenković‐Radak, M. & Andelković, M. (2013). Population specific fitness response of *Drosophila subobscura* to lead pollution. *Insect Science*, 20, 245-253.

Kille, P., Andre, J., Anderson, C., Ang, H.N., Bruford, M.W., Bundy, J.G. *et al.* (2013). DNA sequence variation and methylation in an arsenic tolerant earthworm population. *Soil Biology and Biochemistry*, 57, 524-532.

Kirchhoff, S., Sevigny, J. & Couillard, C. (1999). Genetic and meristic variations in the mummichog *Fundulus heteroclitus*, living in polluted and reference estuaries. *Marine Environmental Research*, 47, 261-283.

Kirkey, F.M., Matthews, J. & Ryser, P. (2012). Metal resistance in populations of red maple (Acer rubrum L.) and white birch (*Betula papyrifera* Marsh.) from a metal-contaminated region and neighbouring non-contaminated regions. *Environmental Pollution*, 164, 53-58.

Klerks, P., Leberg, P., Lance, R., McMillin, D. & Means, J. (1997). Lack of development of pollutant-resistance or genetic differentiation in darter gobies (*Gobionellus boleosoma*) inhabiting a produced-water discharge site. *Marine Environmental Research*, 44, 377-395.

Klerks, P.L. & Moreau, C.J. (2001). Heritability of resistance to individual contaminants and to contaminant mixtures in the sheepshead minnow (*Cyprinodon variegatus*). *Environmental Toxicology and Chemistry*, 20, 1746-1751.

Klerks, P.L., Xie, L. & Levinton, J.S. (2011). Quantitative genetics approaches to study evolutionary processes in ecotoxicology; a perspective from research on the evolution of resistance. *Ecotoxicology*, 20, 513-523.

Knapen, D., Bervoets, L., Verheyen, E. & Blust, R. (2004). Resistance to water pollution in natural gudgeon (*Gobio gobio*) populations may be due to genetic adaptation. *Aquatic Toxicology*, 67, 155-165.

Knapen, D., De Wolf, H., Knaepkens, G., Bervoets, L., Eens, M., Blust, R. *et al.* (2009). Historical metal pollution in natural gudgeon populations: inferences from allozyme, microsatellite and condition factor analysis. *Aquatic Toxicology*, 95, 17-26.

Knapen, D., Reynders, H., Bervoets, L., Verheyen, E. & Blust, R. (2007). Metallothionein gene and protein expression as a biomarker for metal pollution in natural gudgeon populations. *Aquatic Toxicology*, 82, 163-172.

Koch, M., Mummenhoff, K. & Hurka, H. (1998). Systematics and evolutionary history of heavy metal tolerant *Thlaspi caerulescens* in Western Europe: evidence from genetic studies based on isozyme analysis. *Biochemical Systematics and Ecology*, 26, 823-838.

Köhler, H.-R., Eckwert, H., Triebskorn, R. & Bengtsson, G. (1999). Interaction between tolerance and 70kDa stress protein (hsp70) induction in collembolan populations exposed to long-term metal pollution. *Applied Soil Ecology*, 11, 43-52.

Kopp, R.L., Guttman, S.I. & Wissing, T.E. (1992). Genetic indicators of environmental stress in central mudminnow (*Umbra limi*) populations exposed to acid deposition in the Adirondack Mountains. *Environmental Toxicology and Chemistry*, 11, 665-676.

Korshikov, I., Velikoridko, T. & Butilskaya, L. (2002). Genetic structure and variation in *Pinus sylvestris* L. populations degrading due to pollution-induced injury. *Silvae Genetica*, 51, 45-48.

Kováčik, J., Klejdus, B., Hedbavny, J. & Bačkor, M. (2010). Tolerance of *Silene vulgaris* to copper: Population‐related comparison of selected physiological parameters. *Environmental Toxicology*, 25, 581-592.

Kovalchuk, I., Abramov, V., Pogribny, I. & Kovalchuk, O. (2004). Molecular aspects of plant adaptation to life in the Chernobyl zone. *Plant Physiology*, 135, 357-363.

Kuchma, O. & Finkeldey, R. (2011). Evidence for selection in response to radiation exposure: *Pinus sylvestris* in the Chernobyl exclusion zone. *Environmental Pollution*, 159, 1606-1612.

Kurbalija, Z., Kenig, B., Plavša, J., Stamenković-Radak, M. & Anđelković, M. (2010). The effect of lead on the developmental stability of *Drosophila subobscura* through selection in laboratory conditions. *Archives of Biological Sciences*, 62, 83-91.

Langdon, C., Morgan, A., Charnock, J., Semple, K.T. & Lowe, C. (2009). As-resistance in laboratory-reared F1, F2 and F3 generation offspring of the earthworm Lumbricus rubellus inhabiting an As-contaminated mine soil. *Environmental Pollution*, 157, 3114-3119.

Larno, V., Laroche, J., Launey, S., Flammarion, P. & Devaux, A. (2001). Responses of chub (*Leuciscus cephalus*) populations to chemical stress, assessed by genetic markers, DNA damage and cytochrome P4501A induction. *Ecotoxicology*, 10, 145-158.

Lehmann, C. & Rebele, F. (2004). Evaluation of heavy metal tolerance in *Calamagrostis epigejos* and Elymus repens revealed copper tolerance in a copper smelter population of C. epigejos. *Environmental and Experimental Botany*, 51, 199-213.

Lind, E.E. & Grahn, M. (2011). Directional genetic selection by pulp mill effluent on multiple natural populations of three-spined stickleback (*Gasterosteus aculeatus*). *Ecotoxicology*, 20, 503-512.

Liu, J. & Xiong, Z. (2005). Differences in Accumulation and Physiological Response to Copper Stress in three Populations of *Elsholtzia haichowensis* S. *Water, Air, and Soil Pollution*, 168, 5-16.

Lock, K. & Janssen, C. (2002). Multi-generation toxicity of zinc, cadmium, copper and lead to the potworm *Enchytraeus albidus*. *Environmental Pollution*, 117, 89-92.

Lohbeck, K.T., Riebesell, U. & Reusch, T.B. (2012). Adaptive evolution of a key phytoplankton species to ocean acidification. *Nature Geoscience*, 5, 346-351.

Lopes, I., Baird, D. & Ribeiro, R. (2004). Genetic determination of tolerance to lethal and sublethal copper concentrations in field populations of *Daphnia longispina*. *Archives of Environmental Contamination and Toxicology*, 46, 43-51.

Lopes, I., Baird, D.J. & Ribeiro, R. (2005). Resistance to metal contamination by historically-stressed populations of *Ceriodaphnia pulchella*: Environmental influence versus genetic determination. *Chemosphere*, 61, 1189-1197.

Lopes, I., Baird, D.J. & Ribeiro, R. (2006). Genetic adaptation to metal stress by natural populations of *Daphnia longispina*. *Ecotoxicology and Environmental Safety*, 63, 275-285.

López-Rodas, V., Perdigones, N., Marvá, F., Rouco, M. & García-Cabrera, J.A. (2008). Adaptation of phytoplankton to novel residual materials of water pollution: an experimental model analysing the evolution of an experimental microalgal population under formaldehyde contamination. *Bulletin of Environmental Contamination and Toxicology*, 80, 158-162.

Lukkari, T., Taavitsainen, M., Soimasuo, M., Oikari, A. & Haimi, J. (2004). Biomarker responses of the earthworm *Aporrectodea tuberculata* to copper and zinc exposure: differences between populations with and without earlier metal exposure. *Environmental Pollution*, 129, 377-386.

Ma, X.L., Cowles, D. & Carter, R. (2000). Effect of pollution on genetic diversity in the bay mussel Mytilus galloprovincialis and the acorn barnacle *Balanus glandula*. *Marine Environmental Research*, 50, 559-563.

MacNair, M.R., Smith, S.E. & Cumbes, Q.J. (1993). Heritability and distribution of variation in degree of copper tolerance in *Mimulus guttatus* at Copperopolis, California. *Heredity*, 71, 445-445.

Maes, G., Raeymaekers, J., Pampoulie, C., Seynaeve, A., Goemans, G., Belpaire, C. *et al.* (2005). The catadromous European eel *Anguilla anguilla* (L.) as a model for freshwater evolutionary ecotoxicology: relationship between heavy metal bioaccumulation, condition and genetic variability. *Aquatic Toxicology*, 73, 99-114.

Marmiroli, M., Visioli, G., Antonioli, G., Maestri, E. & Marmiroli, N. (2009). Integration of XAS techniques and genetic methodologies to explore Cs-tolerance in *Arabidopsis*. *Biochimie*, 91, 180-191.

Marquis, O., Miaud, C., Ficetola, G.F., Bocher, A., Mouchet, F., Guittonneau, S. *et al.* (2009). Variation in genotoxic stress tolerance among frog populations exposed to UV and pollutant gradients. *Aquatic Toxicology*, 95, 152-161.

Martinez, D.E. & Levinton, J. (1996). Adaptation to heavy metals in the aquatic oligochaete *Limnodrilus hoffmeisteri*: evidence for control by one gene. *Evolution*, 1339-1343.

Martins, N., Bollinger, C., Harper, R.M. & Ribeiro, R. (2009). Effects of acid mine drainage on the genetic diversity and structure of a natural population of *Daphnia longispina*. *Aquatic Toxicology*, 92, 104-112.

Martins, N., Lopes, I., Harper, R.M., Ross, P. & Ribeiro, R. (2007). Differential resistance to copper and mine drainage in *Daphnia longispina*: relationship with allozyme genotypes. *Environmental Toxicology and Chemistry*, 26, 1904-1909.

Marvá, F., López-Rodas, V., Rouco, M., Navarro, M., Toro, F.J., Costas, E. *et al.* (2010). Adaptation of green microalgae to the herbicides simazine and diquat as result of pre-selective mutations. *Aquatic Toxicology*, 96, 130-134.

McKenzie, L.A., Brooks, R. & Johnston, E.L. (2011). Heritable pollution tolerance in a marine invader. *Environmental Research*, 111, 926-932.

McMillan, A.M., Bagley, M.J., Jackson, S.A. & Nacci, D.E. (2006). Genetic diversity and structure of an estuarine fish (*Fundulus heteroclitus*) indigenous to sites associated with a highly contaminated urban harbor. *Ecotoxicology*, 15, 539-548.

Medina, M., Morandi, B. & Correa, J. (2009). Copper effects in the copepod *Tigriopus angulatus* Lang, 1933: natural broad tolerance allows maintenance of food webs in copper-enriched coastal areas. *Marine and Freshwater Research*, 59, 1061-1066.

Menezes-Oliveira, V., Scott-Fordsmand, J., Rocco, A., Soares, A. & Amorim, M. (2011). Interaction between density and Cu toxicity for Enchytraeus crypticus and *Eisenia fetida* reflecting field scenarios. *Science of The Total Environment*, 409, 3370-3374.

Mengoni, A., Barabesi, C., Gonnelli, C., Galardi, F., Gabbrielli, R. & Bazzicalupo, M. (2001). Genetic diversity of heavy metal‐tolerant populations in *Silene paradoxa* L.(Caryophyllaceae): a chloroplast microsatellite analysis. *Molecular Ecology*, 10, 1909-1916.

Mengoni, A., Gonnelli, C., Galardi, F., Gabbrielli, R. & Bazzicalupo, M. (2000). Genetic diversity and heavy metal tolerance in populations of *Silene paradoxa* L.(Caryophyllaceae): a random amplified polymorphic DNA analysis. *Molecular Ecology*, 9, 1319-1324.

Merilä, J., Söderman, F., O'Hara, R., Räsänen, K. & Laurila, A. (2004). Local adaptation and genetics of acid-stress tolerance in the moor frog, *Rana arvalis*. *Conservation Genetics*, 5, 513-527.

Messiaen, M., De Schamphelaere, K.A., Muyssen, B.T. & Janssen, C.R. (2010). The micro-evolutionary potential of *Daphnia magna* population exposed to temperature and cadmium stress. *Ecotoxicology and Environmental Safety*, 73, 1114-1122.

Messiaen, M., Janssen, C., Thas, O. & De Schamphelaere, K. (2012). The potential for adaptation in a natural *Daphnia magna* population: broad and narrow-sense heritability of net reproductive rate under Cd stress at two temperatures. *Ecotoxicology*, 21, 1899-1910.

Messiaen, M., Janssen, C.R., De Meester, L. & De Schamphelaere, K.A.C. (2013). The initial tolerance to sub-lethal Cd exposure is the same among ten naïve pond populations of *Daphnia magna*, but their micro-evolutionary potential to develop resistance is very different. *Aquatic Toxicology*, 144, 322-331.

Meyer, C.L., Vitalis, R., Saumitou‐Laprade, P. & Castric, V. (2009). Genomic pattern of adaptive divergence in *Arabidopsis halleri*, a model species for tolerance to heavy metal. *Molecular Ecology*, 18, 2050-2062.

Meyer, J.N. & Di Giulio, R.T. (2003). Heritable adaptation and fitness costs in killifish (*Fundulus heteroclitus*) inhabiting a polluted estuary. *Ecological Applications*, 13, 490-503.

Meyer, J.N., Smith, J.D., Winston, G.W. & Di Giulio, R.T. (2003a). Antioxidant defenses in killifish (*Fundulus heteroclitus*) exposed to contaminated sediments and model prooxidants: short-term and heritable responses. *Aquatic Toxicology*, 65, 377-395.

Meyer, J.N., Wassenberg, D.M., Karchner, S.I., Hahn, M.E. & DiGiulio, R.T. (2003b). Expression and inducibility of aryl hydrocarbon receptor pathway genes in wild‐caught killifish (*Fundulus heteroclitus*) with different contaminant‐exposure histories. *Environmental Toxicology and Chemistry*, 22, 2337-2343.

Mireji, P., Keating, J., Hassanali, A., Mbogo, C., Muturi, M., Githure, J. *et al.* (2010). Biological cost of tolerance to heavy metals in the mosquito *Anopheles gambiae*. *Medical and Veterinary Entomology*, 24, 101-107.

Moiseenko, T. (2002). Change in the life cycle strategy of fish under the effect of chronic water pollution. *Russian Journal of Ecology*, 33, 45-55.

Monosson, E., Elskus, A., Sharpe, D. & McElroy, A. (1995). How do we study reproduction in contaminated populations: addressing the potential for adaptation to pollution. Society of Environmental Toxicology and Chemistry, Pensacola, FL (United States).

Monserrat, J.M., Martínez, P.E., Geracitano, L.A., Amado, L.L., Martins, C.M.G., Pinho, G.L.L. *et al.* (2007). Pollution biomarkers in estuarine animals: critical review and new perspectives. *Comparative Biochemistry and Physiology Part C: Toxicology & Pharmacology*, 146, 221-234.

Moraga, D., Mdelgi-Lasram, E., Romdhane, M., El Abed, A., Boutet, I., Tanguy, A. *et al.* (2002). Genetic responses to metal contamination in two clams: Ruditapes decussatus and *Ruditapes philippinarum*. *Marine Environmental Research*, 54, 521-525.

Morozova, O. & Marra, M.A. (2008). Applications of next-generation sequencing technologies in functional genomics. *Genomics*, 92, 255-264.

Mulvey, M., Newman, M.C., Chazal, A., Keklak, M.M., Heagler, M.G. & Hales, L.S. (1995). Genetic and demographic responses of mosquitofish (*Gambusia holbrooki* Girard 1859) populations stressed by mercury. *Environmental Toxicology and Chemistry*, 14, 1411-1418.

Mulvey, M., Newman, M.C., Vogelbein, W. & Unger, M.A. (2002). Genetic structure of *Fundulus heteroclitus* from PAH-contaminated and neighboring sites in the Elizabeth and York Rivers. *Aquatic Toxicology*, 61, 195-209.

Murdoch, M.H. & Hebert, P.D. (1994). Mitochondrial DNA diversity of brown bullhead from contaminated and relatively pristine sites in the Great Lakes. *Environmental Toxicology and Chemistry*, 13, 1281-1289.

Nacci, D., Coiro, L., Champlin, D., Jayaraman, S., McKinney, R., Gleason, T. *et al.* (1999). Adaptations of wild populations of the estuarine fish *Fundulus heteroclitus* to persistent environmental contaminants. *Marine Biology*, 134, 9-17.

Nacci, D.E., Champlin, D., Coiro, L., McKinney, R. & Jayaraman, S. (2002). Predicting the occurrence of genetic adaptation to dioxinlike compounds in populations of the estuarine fish *Fundulus heteroclitus*. *Environmental Toxicology and Chemistry*, 21, 1525-1532.

Nacci, D.E., Champlin, D. & Jayaraman, S. (2010). Adaptation of the estuarine fish *Fundulus heteroclitus* (Atlantic killifish) to polychlorinated biphenyls (PCBs). *Estuaries and Coasts*, 33, 853-864.

Nadig, S.G., Lee, K. & Adams, S. (1998). Evaluating alterations of genetic diversity in sunfish populations exposed to contaminants using RAPD assay. *Aquatic Toxicology*, 43, 163-178.

Nakamori, T., Fujimori, A., Kinoshita, K., Ban-nai, T., Kubota, Y. & Yoshida, S. (2010). mRNA expression of a cadmium-responsive gene is a sensitive biomarker of cadmium exposure in the soil collembolan *Folsomia candida*. *Environmental Pollution*, 158, 1689-1695.

Navarro, A., Sánchez-Fontenla, J., Cordero, D., Faria, M., Pena, J.B., Saavedra, C. *et al.* (2013). Genetic and phenoptypic differentiation of zebra mussel populations colonizing Spanish river basins. *Ecotoxicology*, 22, 915-928.

Nielsen, H.D., Brownlee, C., Coelho, S.M. & Brown, M.T. (2003). Inter‐population differences in inherited copper tolerance involve photosynthetic adaptation and exclusion mechanisms in *Fucus serratus*. *New Phytologist*, 160, 157-165.

Nota, B., de Korte, M., Ylstra, B., van Straalen, N.M. & Roelofs, D. (2013). Genetic variation in parthenogenetic collembolans is associated with differences in fitness and cadmium-induced transcriptome responses. *Environmental Science & Technology*, 47, 1155-1162.

Nota, B., Verweij, R.A., Molenaar, D., Ylstra, B., van Straalen, N.M. & Roelofs, D. (2010). Gene expression analysis reveals a gene set discriminatory to different metals in soil. *Toxicological Sciences*, kfq043.

Nota, B., Vooijs, R., van Straalen, N.M. & Roelofs, D. (2011). Expression of mtc in *Folsomia candida* indicative of metal pollution in soil. *Environmental Pollution*, 159, 1343-1347.

Nowak, C., Czeikowitz, A., Vogt, C., Oetken, M., Streit, B. & Schwenk, K. (2008). Variation in sensitivity to cadmium among genetically characterized laboratory strains of the midge *Chironomus riparius*. *Chemosphere*, 71, 1950-1956.

Nowak, C., Vogt, C., Pfenninger, M., Schwenk, K., Oehlmann, J., Streit, B. *et al.* (2009). Rapid genetic erosion in pollutant-exposed experimental chironomid populations. *Environmental Pollution*, 157, 881-886.

Oleksiak, M.F., Karchner, S.I., Jenny, M.J., Franks, D.G., Welch, D.B.M. & Hahn, M.E. (2011). Transcriptomic assessment of resistance to effects of an aryl hydrocarbon receptor (AHR) agonist in embryos of Atlantic killifish (*Fundulus heteroclitus*) from a marine Superfund site. *BMC Genomics*, 12, 263.

Olsvik, P.A., Hindar, K., Zachariassen, K.E. & Andersen, R.A. (2001). Brown trout (*Salmo trutta*) metallothioneins as biomarkers for metal exposure in two Norwegian rivers. *Biomarkers*, 6, 274-288.

Ownby, D.R., Newman, M.C., Mulvey, M., Vogelbein, W.K., Unger, M.A. & Arzayus, L.F. (2002). Fish (*Fundulus heteroclitus*) populations with different exposure histories differ in tolerance of creosote‐contaminated sediments. *Environmental Toxicology and Chemistry*, 21, 1897-1902.

Pakkasmaa, S., Merilä, J. & O'Hara, R. (2003). Genetic and maternal effect influences on viability of common frog tadpoles under different environmental conditions. *Heredity*, 91, 117-124.

Parker, L.M., Ross, P.M., O'Connor, W.A., Borysko, L., Raftos, D.A. & Pörtner, H.O. (2012). Adult exposure influences offspring response to ocean acidification in oysters. *Global Change Biology*, 18, 82-92.

Patra, J. & Panda, B.B. (1998). A comparison of biochemical responses to oxidative and metal stress in seedlings of barley, *Hordeum vulgare* L. *Environmental Pollution*, 101, 99-105.

Pauwels, M., Saumitou‐Laprade, P., Holl, A.C., Petit, D. & Bonnin, I. (2005). Multiple origin of metallicolous populations of the pseudometallophyte *Arabidopsis halleri* (Brassicaceae) in central Europe: the cpDNA testimony. *Molecular Ecology*, 14, 4403-4414.

Pease, C.J., Johnston, E.L. & Poore, A.G. (2010). Genetic variability in tolerance to copper contamination in a herbivorous marine invertebrate. *Aquatic Toxicology*, 99, 10-16.

Pespeni, M.H., Sanford, E., Gaylord, B., Hill, T.M., Hosfelt, J.D., Jaris, H.K. *et al.* (2013). Evolutionary change during experimental ocean acidification. *Proceedings of the National Academy of Sciences*, 110, 6937-6942.

Phillips, N. & Hickey, C. (2010). Genotype-dependent recovery from acute exposure to heavy metal contamination in the freshwater clam *Sphaerium novaezelandiae*. *Aquatic Toxicology*, 99, 507-513.

Piola, R.F. & Johnston, E.L. (2006). Differential tolerance to metals among populations of the introduced bryozoan *Bugula neritina*. *Marine Biology*, 148, 997-1010.

Pistevos, J.C., Calosi, P., Widdicombe, S. & Bishop, J.D. (2011). Will variation among genetic individuals influence species responses to global climate change? *Oikos*, 120, 675-689.

Plejdrup, J., Simonsen, V., Pertoldi, C., Schøyen, M. & Bayley, M. (2006). Genetic and morphological diversity in populations of *Nucella lapillus* (L.; neogastropoda) in response to tributyltin contamination. *Ecotoxicology and Environmental Safety*, 64, 146-154.

Polyak, Y., Zaytseva, T. & Medvedeva, N. (2013). Response of toxic cyanobacterium *Microcystis aeruginosa* to environmental pollution. *Water, Air, & Soil Pollution*, 224, 1-14.

Posthuma, L., Hogervorst, R.F., Joosse, E.N. & Van Straalen, N.M. (1993a). Genetic variation and covariation for characteristics associated with cadmium tolerance in natural populations of the springtail *Orchesella cincta* (L.). *Evolution*, 619-631.

Posthuma, L., Hogervorst, R.F. & Van Straalen, N.M. (1992). Adaptation to soil pollution by cadmium excretion in natural populations of *Orchesella cincta* (L.)(Collembola). *Archives of Environmental Contamination and Toxicology*, 22, 146-156.

Posthuma, L., Verweij, R.A., Widianarko, B. & Zonneveld, C. (1993b). Life-history patterns in metal-adapted Collembola. *Oikos*, 235-249.

Postma, J.F. & Davids, C. (1995). Tolerance induction and life cycle changes in cadmium-exposed *Chironomus riparius* (Diptera) during consecutive generations. *Ecotoxicology and Environmental Safety*, 30, 195-202.

Postma, J.F., Kyed, M. & Admiraal, W. (1995a). Alterations in life-history traits of *Chironomus riparius* (Diptera) obtained from metal contaminated rivers. *Hydrobiologia*, 315, 159-165.

Postma, J.F., Kyed, M. & Admiraal, W. (1995b). Site specific differentiation in metal tolerance in the midge *Chironomus riparius* (Diptera, Chironomidae). *Hydrobiologia*, 315, 159-165.

Postma, J.F., VanNugteren, P. & De Jong, M.B.B. (1996). Increased cadmium excretion in metal‐adapted populations of the midge *Chironomus riparius* (diptera). *Environmental Toxicology and Chemistry*, 15, 332-339.

Prus-Głowacki, W., Chudzińska, E., Wojnicka-Półtorak, A., Kozacki, L. & Fagiewicz, K. (2006). Effects of heavy metal pollution on genetic variation and cytological disturbances in the *Pinus sylvestris* L. population. *Journal of Applied Genetics*, 47, 99-108.

Prus-Glowacki, W. & Godzik, S. (1995). Genetic structure of *Picea abies* trees tolerant and sensitive to industrial pollution. *Silvae Genetica*, 44, 62-65.

Prus-Glowacki, W., Wojnicka-Poltorak, A., Oleksyn, J. & Reich, P. (1999). Industrial pollutants tend to increase genetic diversity: evidence from field-grown European Scots pine populations. *Water, Air, and Soil Pollution*, 116, 395-402.

Przedpełska, E. & Wierzbicka, M. (2007). *Arabidopsis arenosa* (Brassicaceae) from a lead–zinc waste heap in southern Poland–a plant with high tolerance to heavy metals. *Plant and Soil*, 299, 43-53.

Pujolar, J.M., Marino, I.A., Milan, M., Coppe, A., Maes, G.E., Capoccioni, F. et al. (2012). Surviving in a toxic world: transcriptomics and gene expression profiling in response to environmental pollution in the critically endangered European eel. *BMC Genomics*, 13, 507.

Räsänen, K.R., Laurila, A. & Merilä, J. (2003). Geographic variation in acid stress tolerance of the moor frog, *Rana arvalis*. I. Local adaptation. *Evolution*, 57, 352-362.

Regier, N., Baerlocher, L., Münsterkötter, M., Farinelli, L. & Cosio, C. (2013). Analysis of the *Elodea nuttallii* transcriptome in response to mercury and cadmium pollution: development of sensitive tools for rapid ecotoxicological testing. *Environmental Science & Technology*, 47, 8825-8834.

Reitzel, A.M., Karchner, S.I., Franks, D.G., Evans, B.R., Nacci, D., Champlin, D. *et al.* (2014). Genetic variation at aryl hydrocarbon receptor (AHR) loci in populations of Atlantic killifish (*Fundulus heteroclitus*) inhabiting polluted and reference habitats. *BMC Evolutionary Biology*, 14, 6.

Remon, E., Bouchardon, J.-L. & Faure, O. (2007). Multi-tolerance to heavy metals in *Plantago arenaria* Waldst. & Kit.: adaptative versus constitutive characters. *Chemosphere*, 69, 41-47.

Ritter, A., Ubertini, M., Romac, S., Gaillard, F., Delage, L., Mann, A. *et al.* (2010). Copper stress proteomics highlights local adaptation of two strains of the model brown alga *Ectocarpus siliculosus*. *Proteomics*, 10, 2074-2088.

Roark, S.A., Nacci, D., Coiro, L., Champlin, D. & Guttman, S.I. (2005). Population genetic structure of a nonmigratory estuarine fish (*Fundulus heteroclitus*) across a strong gradient of polychlorinated biphenyl contamination. *Environmental Toxicology and Chemistry*, 24, 717-725.

Roelofs, D., Janssens, T.K., Timmermans, M.J., Nota, B., Marien, J., Bochdanovits, Z. *et al.* (2009). Adaptive differences in gene expression associated with heavy metal tolerance in the soil arthropod *Orchesella cincta*. *Molecular Ecology*, 18, 3227-3239.

Roelofs, D., Marien, J. & van Straalen, N.M. (2007). Differential gene expression profiles associated with heavy metal tolerance in the soil insect *Orchesella cincta*. *Insect Biochemistry and Molecular Biology*, 37, 287-295.

Roelofs, D., Overhein, L., De Boer, M., Janssens, T. & Van Straalen, N. (2006). Additive genetic variation of transcriptional regulation: metallothionein expression in the soil insect *Orchesella cincta*. *Heredity*, 96, 85-92.

Ross, K., Cooper, N., Bidwell, J.R. & Elder, J. (2002). Genetic diversity and metal tolerance of two marine species: a comparison between populations from contaminated and reference sites. *Marine Pollution Bulletin*, 44, 671-679.

Rożen, A. (2006). Effect of cadmium on life-history parameters in Dendrobaena octaedra (Lumbricidae: Oligochaeta) populations originating from forests differently polluted with heavy metals. *Soil Biology and Biochemistry*, 38, 489-503.

Sabri, D.M., Rabie, T., Ahmed, A.I., Zakaria, S., Bourdineaud, J.P. (2012). Heavy Metals-Induced Expression of ABCB10 Gene in Zebrafish *Danio rerio*. *Physiology and Molecular Biology*,4, 97-106.

Salice, C.J., Anderson, T.A. & Roesijadi, G. (2010). Adaptive responses and latent costs of multigeneration cadmium exposure in parasite resistant and susceptible strains of a freshwater snail. *Ecotoxicology*, 19, 1466-1475.

Salminen, J. & Haimi, J. (2001). The asexual enchytraeid worm *Cognettia sphagnetorum* (Oligochaeta) has increased Cu resistance in polluted soil. *Environmental Pollution*, 113, 221-224.

Schat, H., Vooijs, R. & Kuiper, E. (1996). Identical major gene loci for heavy metal tolerances that have independently evolved in different local populations and subspecies of *Silene vulgaris*. *Evolution*, 1888-1895.

Schizas, N., Chandler, G., Coull, B., Klosterhaus, S. & Quattro, J. (2001). Differential survival of three mitochondrial lineages of a marine benthic copepod exposed to a pesticide mixture. *Environmental Science & Technology*, 35, 535-538.

Schlueter, M.A., Guttman, S.I., Oris, J.T. & Bailer, A.J. (1995). Survival of copper‐exposed juvenile fathead minnows (*Pimephales promelas*) differs among allozyme genotypes. *Environmental Toxicology and Chemistry*, 14, 1727-1734.

Schlüter, L., Lohbeck, K.T., Gutowska, M.A., Gröger, J.P., Riebesell, U. & Reusch, T.B. (2014). Adaptation of a globally important coccolithophore to ocean warming and acidification. *Nature Climate Change*, 4, 1024-1030.

Shaw, J.R., Colbourne, J.K., Davey, J.C., Glaholt, S.P., Hampton, T.H., Chen, C.Y. *et al.* (2007). Gene response profiles for *Daphnia pulex* exposed to the environmental stressor cadmium reveals novel crustacean metallothioneins. *BMC Genomics*, 8, 477.

Shirley, M.D. & Sibly, R.M. (1999). Genetic basis of a between-environment trade-off involving resistance to cadmium in *Drosophila melanogaster*. *Evolution*, 826-836.

Silva, R.M., Pereira, F., Carneiro, J., Sobral, O., Ribeiro, R., Amorim, A. *et al.* (2010). Microevolution in a natural population of *Daphnia longispina* exposed to acid mine drainage. *Interdisciplinary Studies on Environmental Chemistry—Biological Responses to Contaminants, Eds., N.. Hamamura, S.. Suzuki, S.. Mendo, CM. Barroso, H.. Iwata and S.. Tanabe*, 213-218.

Słomka, A., Sutkowska, A., Szczepaniak, M., Malec, P., Mitka, J. & Kuta, E. (2011). Increased genetic diversity of *Viola tricolor* L.(Violaceae) in metal-polluted environments. *Chemosphere*, 83, 435-442.

Snyder, C. & Hendricks, A. (1997). Genetic responses of *Isonychia bicolor* (Ephemeroptera: Isonychiidae) to chronic mercury pollution. *Journal of the North American Benthological Society*, 651-663.

Soeter, A., Bakker, F., Velthuis, M., Verweij, R., Hoitinga, L., Marinkovic, M. *et al.* (2010). The selective environment: genetic adaptation of the midge *Chironomus riparius* to metal pollution. *Proceedings of the Netherlands Entomological Society Meeting*, 21, 85-94.

Sokolowski, A., Fichet, D., Garcia-Meunier, P., Radenac, G., Wolowicz, M. & Blanchard, G. (2002). The relationship between metal concentrations and phenotypes in the Baltic clam *Macoma balthica* (L.) from the Gulf of Gdansk, southern Baltic. *Chemosphere*, 47, 475-484.

Spurgeon, D. & Hopkin, S. (1999). Tolerance to zinc in populations of the earthworm *Lumbricus rubellus* from uncontaminated and metal-contaminated ecosystems. *Archives of Environmental Contamination and Toxicology*, 37, 332-337.

Spurgeon, D. & Hopkin, S. (2000). The development of genetically inherited resistance to zinc in laboratory-selected generations of the earthworm *Eisenia fetida*. *Environmental Pollution*, 109, 193-201.

Štambuk, A., Šrut, M., Šatović, Z., Tkalec, M. & Klobučar, G.I. (2013). Gene flow vs. pollution pressure: Genetic diversity of *Mytilus galloprovincialis* in eastern Adriatic. *Aquatic Toxicology*, 136, 22-31.

Staton, J., Schizas, N., Chandler, G., Coull, B. & Quattro, J. (2001). Ecotoxicology and population genetics: the emergence of ‘phylogeographic and evolutionary ecotoxicology’. *Ecotoxicology*, 10, 217-222.

Sterenborg, I. & Roelofs, D. (2003). Field-selected cadmium tolerance in the springtail *Orchesella cincta* is correlated with increased metallothionein mRNA expression. *Insect Biochemistry and Molecular Biology*, 33, 741-747.

Street, G., Lotufo, G., Montagna, P. & Fleeger, J. (1998). Reduced genetic diversity in a meiobenthic copepod exposed to a xenobiotic. *Journal of Experimental Marine Biology and Ecology*, 222, 93-111.

Stürzenbaum, S., Kille, P. & Morgan, A. (1998a). Heavy metal-induced molecular responses in the earthworm, *Lumbricus rubellus* genetic fingerprinting by directed differential display. *Applied Soil Ecology*, 9, 495-500.

Stürzenbaum, S., Kille, P. & Morgan, A. (1998b). Identification of heavy metal induced changes in the expression patterns of the translationally controlled tumour protein (TCTP) in the earthworm *Lumbricus rubellus*. *Biochimica et Biophysica Acta (BBA)-Gene Structure and Expression*, 1398, 294-304.

Sunday, J.M., Crim, R.N., Harley, C.D. & Hart, M.W. (2011). Quantifying rates of evolutionary adaptation in response to ocean acidification. *PLoS One*, 6, e22881.

Tanguy, A., Castro, N.F., Marhic, A. & Moraga, D. (1999). Effects of an organic pollutant (tributyltin) on genetic structure in the Pacific oyster *Crassostrea gigas*. *Marine Pollution Bulletin*, 38, 550-559.

Theodorakis, C.W., Bickham, J.W., Elbl, T., Shugart, L.R. & Chesser, R.K. (1998). Genetics of radionuclide‐contaminated mosquitofish populations and homology between *Gambusia affinis* and *G. holbrooki*. *Environmental Toxicology and Chemistry*, 17, 1992-1998.

Theodorakis, C.W., Elbl, T. & Shugart, L.R. (1999). Genetic ecotoxicology IV: survival and DNA strand breakage is dependent on genotype in radionuclide-exposed mosquitofish. *Aquatic Toxicology*, 45, 279-291.

Theodorakis, C.W. & Shugart, L.R. (1997). Genetic ecotoxicology II: population genetic structure in mosquitofish exposed in situ to radionuclides. *Ecotoxicology*, 6, 335-354.

Timmermans, M., Ellers, J. & Van Straalen, N. (2007). Allelic diversity of metallothionein in *Orchesella cincta* (L.): traces of natural selection by environmental pollution. *Heredity*, 98, 311-319.

Timmermans, M.J., Ellers, J., Roelofs, D. & van Straalen, N.M. (2005). Metallothionein mRNA expression and cadmium tolerance in metal-stressed and reference populations of the springtail *Orchesella cincta*. *Ecotoxicology*, 14, 727-739.

Tranvik, L., Bengtsson, G. & Rundgren, S. (1993). Relative abundance and resistance traits of two Collembola species under metal stress. *Journal of Applied Ecology*, 43-52.

Turner, A.P. & Dickinson, N.M. (1993). Copper tolerance of *Acer pseudoplatanus* L.(sycamore) in tissue culture. *New Phytologist*, 123, 523-530.

Van Rossum, F., Bonnin, I., Fenart, S., Pauwels, M., Petit, D. & Saumitou-Lapadre, P. (2004). Spatial genetic structure within a metallicolous population of *Arabidopsis halleri*, a clonal, self‐incompatible and heavy‐metal‐tolerant species. *Molecular Ecology*, 13, 2959-2967.

Van Straalen, N.M., Janssens, T.K. & Roelofs, D. (2011). Micro-evolution of toxicant tolerance: from single genes to the genome’s tangled bank. *Ecotoxicology*, 20, 574-579.

Venier, P., De Pittà, C., Pallavicini, A., Marsano, F., Varotto, L., Romualdi, C. *et al.* (2006). Development of mussel mRNA profiling: can gene expression trends reveal coastal water pollution? *Mutation Research/Fundamental and Molecular Mechanisms of Mutagenesis*, 602, 121-134.

Vidal, D.E. & Horne, A.J. (2003). Inheritance of mercury tolerance in the aquatic oligochaete *Tubifex tubifex*. *Environmental Toxicology and Chemistry*, 22, 2130-2135.

Virgilio, M. & Abbiati, M. (2004). Allozyme genotypes and tolerance to copper stress in *Hediste diversicolor* (Polychaeta: Nereididae). *Marine Pollution Bulletin*, 49, 978-985.

Vogt, C., Nowak, C., Diogo, J.B., Oetken, M., Schwenk, K. & Oehlmann, J. (2007). Multi-generation studies with *Chironomus riparius*–effects of low tributyltin concentrations on life history parameters and genetic diversity. *Chemosphere*, 67, 2192-2200.

Ward, T.J. & Robinson, W.E. (2005). Evolution of cadmium resistance in *Daphnia magna*. *Environmental Toxicology and Chemistry*, 24, 2341-2349.

Wasowicz, P., Pielichowska, M., Przedpelska-Wasowicz, E.M., Bednarek, P., Szarek-Lukaszewska, G., Abratowska, A. *et al.* (2014). Physiological and genetic differentiation between metallicolous and non-metallicolous diploid populations of alpine *Biscutella laevigata* (Brassicacae) in the Tatra Mountains and the northern Carpathian foreland. In: *Annales Botanici Fennici*, 51, 227-239.

Weston, D.P., Poynton, H.C., Wellborn, G.A., Lydy, M.J., Blalock, B.J., Sepulveda, M.S. *et al.* (2013). Multiple origins of pyrethroid insecticide resistance across the species complex of a nontarget aquatic crustacean, *Hyalella azteca*. *Proceedings of the National Academy of Sciences*, 110, 16532-16537.

Whitehead, A., Anderson, S.L., Kuivila, K.M., L Roach, J. & May, B. (2003). Genetic variation among interconnected populations of *Catostomus occidentalis*: implications for distinguishing impacts of contaminants from biogeographical structuring. *Molecular Ecology*, 12, 2817-2833.

Whitehead, A., Pilcher, W., Champlin, D. & Nacci, D. (2012). Common mechanism underlies repeated evolution of extreme pollution tolerance. *Proceedings of the Royal Society B: Biological Sciences*, 279, 427-433.

Whitehead, A., Triant, D., Champlin, D. & Nacci, D. (2010). Comparative transcriptomics implicates mechanisms of evolved pollution tolerance in a killifish population. *Molecular Ecology*, 19, 5186-5203.

Wierzbicka, M. & Panufnik, D. (1998). The adaptation of *Silene vulgaris* to growth on a calamine waste heap (S. Poland). *Environmental Pollution*, 101, 415-426.

Wierzbicka, M. & Pielichowska, M. (2004). Adaptation of *Biscutella laevigata* L, a metal hyperaccumulator, to growth on a zinc–lead waste heap in southern Poland: I: Differences between waste-heap and mountain populations. *Chemosphere*, 54, 1663-1674.

Wilczek, G., Babczynska, A., Migula, P. & Wencelis, B. (2003). Activity of esterases as biomarkers of metal exposure in spiders from the metal pollution gradient. *Polish Journal of Environmental Studies*, 12, 765-772.

Willems, G., Dräger, D.B., Courbot, M., Godé, C., Verbruggen, N. & Saumitou-Laprade, P. (2007). The genetic basis of zinc tolerance in the metallophyte *Arabidopsis halleri* ssp. halleri (Brassicaceae): an analysis of quantitative trait loci. *Genetics*, 176, 659-674.

Williams, L.M. & Oleksiak, M.F. (2008). Signatures of selection in natural populations adapted to chronic pollution. *BMC Evolutionary Biology*, 8, 282.

Williams, L.M. & Oleksiak, M.F. (2011). Ecologically and evolutionarily important SNPs identified in natural populations. *Molecular Biology and Evolution*, 28, 1817-1826.

Wirgin, I., Roy, N.K., Loftus, M., Chambers, R.C., Franks, D.G. & Hahn, M.E. (2011). Mechanistic basis of resistance to PCBs in Atlantic tomcod from the Hudson River. *Science*, 331, 1322-1325.

Wirgin, I. & Waldman, J.R. (2004). Resistance to contaminants in North American fish populations. *Mutation Research/Fundamental and Molecular Mechanisms of Mutagenesis*, 552, 73-100.

Wójcik, M., Dresler, S., Jawor, E., Kowalczyk, K. & Tukiendorf, A. (2013). Morphological, physiological, and genetic variation between metallicolous and nonmetallicolous populations of *Dianthus carthusianorum*. *Chemosphere*, 90, 1249-1257.

Wojnicka-Półtorak, A. (1997). Changes of genetic structure of *Pinus sylvestris* L. populations exposed to industrial pollution. *Acta Societatis Botanicorum Poloniae*, 66, 73-78.

Xie, L. & Klerks, P.L. (2003). Responses to selection for cadmium resistance in the least killifish, *Heterandria formosa*. *Environmental Toxicology and Chemistry*, 22, 313-320.

Xie, Y., Luo, H., Hu, L., Sun, X., Lou, Y. & Fu, J. (2014). Classification of genetic variation for cadmium tolerance in Bermudagrass [Cynodon dactylon (L.) Pers.] using physiological traits and molecular markers. *Ecotoxicology*, 23, 1030-1043.

Yap, C., Cheng, W., Ong, C. & Tan, S. (2013). Heavy Metal Contamination and Physical Barrier are Main Causal Agents for the Genetic Differentiation of Perna viridis Populations in Peninsular Malaysia. *Sains Malaysiana*, 42, 1557-1564.

Yap, C.K., Tan, S.G., Ismail, A. & Omar, H. (2004). Allozyme polymorphisms and heavy metal levels in the green-lipped mussel *Perna viridis* (Linnaeus) collected from contaminated and uncontaminated sites in Malaysia. *Environment International*, 30, 39-46.

Załecka, R. & Wierzbicka, M. (2002). The adaptation of *Dianthus carthusianorum* L. (Caryophyllaceae) to growth on a zinc–lead heap in southern Poland. *Plant and soil*, 246, 249-257.
